# Hysteresis in cavitation emissions during a ramped-then-deramped amplitude sonication: A theoretical and experimental investigation

**DOI:** 10.1007/s11071-026-12462-3

**Published:** 2026-04-21

**Authors:** Yikai Zhang, Shida Li, Paul Prentice, Andrea Cammarano

**Affiliations:** 1 Cavitation Laboratory, Centre for Medical and Industrial Ultrasonics, University of Glasgow, Glasgow, UK; 2 Department of Aeronautics and Astronautics, University of Southampton, Southampton, UK

**Keywords:** Acoustic cavitation, Hysteresis, Spectrogram, Period-doubling bifurcation, Basins of attraction, Bubble dynamics, Nonlinear dynamics

## Abstract

Dynamic hysteresis in cavitation emissions is investigated experimentally and numerically using a continuous ramped-then-deramped acoustic excitation. Hydrophone spectrograms reveal clear asymmetries between the ramped and deramped phases at identical excitation amplitudes, including differences in harmonic, subharmonic, and broadband components. These observations show that the acoustic state of the bubble population depends on the excitation history. A fully coupled 12-bubble model, incorporating spatially resolved interaction forces, reproduces all salient experimental features, including the subharmonic route to chaos, broadband emission, and broadband clearing. Poincaré mapping and continuation analysis reveal multiple coexisting attractors, distinguished by oscillation amplitude and period, delimited by saddle-node and period-doubling bifurcations. The hysteresis observed under continuous excitation arises from history-dependent convergence to these attractors. Basin-of-attraction computations demonstrate that attractor selection depends jointly on initial conditions and bubble-bubble interaction, enabling different bubbles to converge to distinct attractors under identical excitation amplitudes. Together, these results establish a physically interpretable nonlinear-dynamical mechanism underlying hysteresis in cavitation emission.

**Supplementary Information:**

The online version contains supplementary material available at 10.1007/s11071-026-12462-3.

## Introduction

Hysteresis is a dynamical phenomenon in which the response of a nonlinear system depends not only on its current input but also on the history of previous inputs. Therefore, when a change in a parameter alters the response state, reversing that change does not restore the system to its original state [1]. Such behavior is widely observed across physical and biological systems [2–4]. In magnetism, for example, all ferromagnetic materials display hysteresis in the relationship between magnetic flux density and applied magnetic field strength [5, 6]. In materials science, hysteresis is evident in the stress–strain behavior of solids undergoing elastic and plastic deformation, particularly when energy is dissipated or permanent deformation occurs [7]. Phase transitions also exhibit hysteresis: materials with thermal hysteresis solidify at a temperature lower than their melting point [8]. In biological neural networks, certain neurons can exhibit two stable states, such as bursting or tonic firing, and the stimuli for transitioning between these states differ depending on whether the stimulus is increasing or decreasing, demonstrating hysteresis [9].

Hysteresis can be classified into two types: static and dynamic [10]. Static hysteresis typically involves the convergence of response to a fixed point and arises from multi-stability within the system. Dynamic hysteresis, by contrast, involves the evolution of the system towards a limit cycle and results from the coexistence of multiple attractors – the steady-state responses in the system’s phase space toward which trajectories converge for a wide range of initial conditions. Sinusoidal excitation is a common driver of dynamic hysteresis, as it can induce periodic responses in nonlinear systems. For instance, Gao et al. [11] demonstrated that magnetic hysteresis can be static under non-sinusoidal excitation, but becomes dynamic when sinusoidal excitation is applied. In the present study of an acoustic cavitation system, the presence of dynamic hysteresis is shown through the analysis of the steady-state responses under sinusoidal excitation.

Acoustic cavitation refers to bubble activity in liquids exposed to the pressure fluctuations of an ultrasonic or acoustic waveform [12]. Bubble oscillations can be highly energetic and nonlinear, particularly around the collapse phases when high temperatures and pressures can generate free radical species and picosecond flashes of light, known as sonoluminescence [13]. Extreme mechanical effects include shockwave emissions and the formation of high-speed liquid jets, due to asymmetric collapse in proximity to a boundary [14], or due to the action of a pressure gradient within the ultrasonic waveform [15]. The phenomenon has a number of well-established medical and industrial applications, with many more at various stages of development. Cavitation is known to play a pivotal role in stone breaking during lithotripsy [16] and more recently, drug delivery to the brain using transcranial focused ultrasound and contrast agent microbubbles [17], with in-human clinical trials currently underway [18, 19]. Acoustic cleaning is the best-known industrial application [20], while a sizeable literature on sonochemistry and sonoprocessing continues to grow [21].

For many applications, particularly those for which cavitation damage through overexposure is of concern, detection of the cavitation emission signal offers a convenient method through which to monitor and regulate bubble activity. As with the activity itself, the emission signal can be highly nonlinear, including harmonic, sub- and ultra-harmonic components of the excitation frequency, as well as broadband noise emissions [22]. It is generally well recognized that the intensity of the excitation is primarily responsible for the emission components generated, with higher intensities required for subharmonics and broadband noise. Monitoring for the emergence of subharmonic components during focused ultrasound exposure of the brain, for example, is undertaken for safety purposes and avoidance of long-term tissue damage [23–25].

Although bubble-based mechanistic sources for many of the nonlinear components of the cavitation emission signal have now been established [26–29], a number of ‘higher-order’ effects have been described in the literature, but remain under-investigated and poorly understood. For example, a seminal work by Lauterborn and Cramer [30] reported the *subharmonic route to acoustic chaos*, for the emission signal generated during a continuously ramped amplitude sonication, within a radially oscillating tube transducer. This demonstrated the progression through harmonic, sub- and ultra-harmonics of increasing order and the emergence of broadband noise, as the amplitude increased. The spectrogram of the signal collected, however, also revealed unexpected regions of broadband noise clearing, referred to as ‘reverse bifurcations’ in the original paper. We have recently demonstrated, with a similar experimental configuration, that phase synchronization of the bubble oscillations is responsible for sudden reductions in the noise, which itself is generated by asynchronous collapse timings from across the cavitating population [31].

Another factor that can influence the emission signal generated by a given sonication is the recent ‘sonication history’ of the host medium. There are two previous experimental reports describing such hysteretic effects, insofar as we are aware; Frohly, et al. [32] and Seya, et al. [33]. In both, spectra are presented for cavitation emission signals collected during a series of sonications, generated in quick succession, with first increasing and then decreasing intensities. It was shown that for a sonication at any given intensity value, the spectral features generated are sensitive to the intensity of the previous sonications. Specifically, if a sonication at a given intensity was part of a series of decreasing intensities, then the spectrum was found to contain more inertial features, such as subharmonics and broadband noise at higher magnitudes, than for a sonication at the same intensity that was part of a series of increasing intensities. Both studies confirmed this trend using the mean magnitude of the acoustic noise spectra to represent the degree of inertial cavitation activity. The resulting hysteresis loops illustrated the differences between sonications at the same intensity, but part of an increasing or decreasing intensity series.

By way of theoretical validation, Seya, et al. [33] adopted the model of Yasui, et al. [34], using a Rayleigh-Plesset equation to simulate a population of *N* bubbles. Both studies applied a coupling strength, functioning as a simplified proxy for interaction effects. The coupling strength was averaged over a number of uniformly distributed bubbles, with a random perturbation included to account for changes in bubble number, due to fragmentation. Employing the averaged coupling strength, both models substantially simplify the problem by reducing the multi-bubble system to a single bubble. In addition to the model of Yasui, et al. [34], Seya et al. [33] introduced rectified diffusion; the gradual increase in the equilibrium radius due to nonlinear oscillations, which leads to a net influx of dissolved gas [12]. This was not, however, modeled directly in the Rayleigh-Plesset equation. Instead, its influence was approximated by manually increasing the equilibrium radius after each sonication period. Additionally, the coupling strength was neglected. It was introduced to the Rayleigh-Plesset equation only afterwards, for the purpose of estimating the acoustic noise. Buoyancy and dissolution effects between successive sonications were implemented by removing bubbles whose radii exceeded preset thresholds – large enough to rise under buoyancy or small enough to dissolve into the liquid.

In this paper, we extend our recent work *revisiting the subharmonic route to chaos* [31], which employed a continuously ramped amplitude sonication, to include a symmetrical deramped phase, for investigating hysteresis within the emission signal. The fully coupled multi-bubble model with which we probed the synchronization phenomenon is also used to interrogate the memory-dependent nonlinear dynamics, under a ramped-then-deramped excitation. In this framework, bubble-bubble interactions evolve continuously over time, influencing the dynamics throughout the sonication without averaging or stochastic treatments.

## Experimental observations

**Fig. 1:**
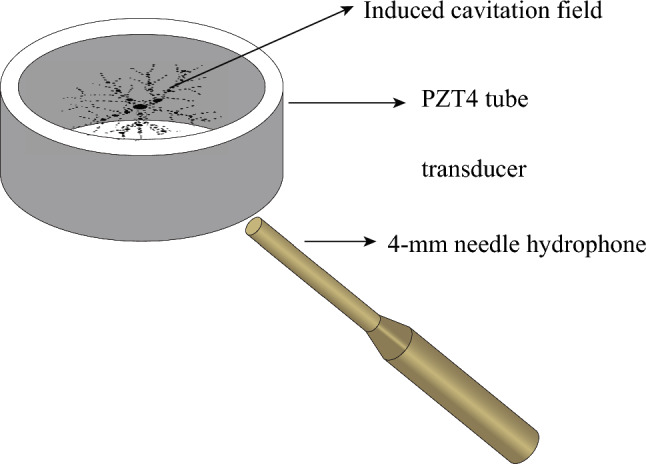
**a** A schematic representation of the key components of the experimental set-up; the tube transducer which generates and drives the cavitation under ramped-then-deramped excitation, and the needle hydrophone which collects the cavitation emission signal. The internal diameter of the tube transducer is 40 mm

**Fig. 2:**
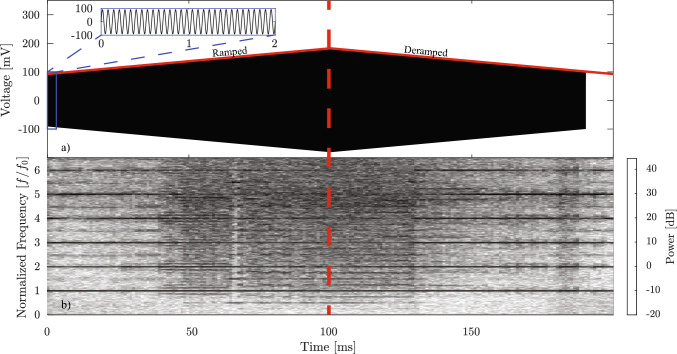
**a** The transducer excitation signal over a total duration of 200 ms. The inset shows a zoom on 0-2 ms. The red lines show the amplitude ramp from 90 mV to 180 mV, and symmetric deramp. The vertical red dashed line is the symmetrical fold between ramp and deramp. **b** Experimental spectrogram of the cavitation emission signal collected during a ramped-then-deramped sonication

The experimental arrangement used for this work is described in detail in [31]. Briefly, a tube transducer (CTS Ferroperm) with an internal diameter of 40 mm, a 4 mm wall thickness and a length of 25 mm, Fig. 1, was submerged within a tank of water. In our experiments, the water temperature was maintained at 22 $^\circ \textrm{C}$, and the dissolved oxygen (DO) content of the deionized water was approximately 8.2 mg/L (DO9100, Tilswall, UK), consistent with air-saturated conditions at standard atmospheric pressure [35]. Under these conditions, cavitation is expected to be air-dominated, which supports the use of a polytropic exponent representative of air in the present model (see Sect. 3). It was excited at its radial resonance by a voltage waveform of frequency $f_0$ = 15.5 kHz, from a signal generator (AFG31052, Tektronix) passed through a 55 dB power amplifier (1040 L, Electronics & Innovation). The pre-amplification peak-positive amplitude of the waveform increased from 90-180 mV over 100 ms, and for this work included a symmetrical deramped phase, Fig. 2a. This continuous ramped-then-deramped excitation, unlike prior hysteresis studies that used discrete series of intensities with off-intervals between fixed-amplitude sonications [32, 33], preserves the previous cavitation field as the amplitude evolves.

The emission signal from the cavitation generated within the bore of the tube was detected by a needle hydrophone (4 mm diameter tip, Precision Acoustics Ltd) with sensitivity from 10 kHz to 8 MHz, Fig. 1, and recorded on an oscilloscope (MSO56, Tektronix) at a sampling frequency of 125 MHz. A time-frequency spectrogram was generated from the hydrophone output, Fig. 2b, using 200 short-time Fourier transforms with 65% overlap and a Hamming window, to suppress discontinuities.

The spectrogram of Fig. 2b indicates that cavitation is induced within the bore of the tube transducer, from the start of the sonication. For the first $\sim$40 ms, over which the amplitude increases from 90-126 mV, strong harmonic lines at integer multiples of the excitation frequency $nf_0$, along with subharmonic components at $nf_0/2$, are observed. Low-magnitude features below $f_0$ are due to reduced hydrophone sensitivity at these frequencies. As the excitation amplitude increases further, broadband noise, attributable to increasingly asynchronous bubble collapse phases across the cavitating population [29, 31, 36], emerges from 40-66 ms. Here, synchronous oscillations denote bubbles collapsing in phase with one another, whereas asynchronous oscillations occur when their collapse timings differ. At approximately 66 ms (149.4 mV), a sudden reduction in broadband noise occurs, accompanied by a strengthening of the $nf_0/2$ lines and a bifurcation – a qualitative change in the system’s long-term behavior (such as the appearance of new attractors or changes in stability) – to include $nf_0/4$ emissions. This broadband clearing indicates an occurrence of synchronization for the bubble oscillations, as has been described for an equivalent ramp-only sonication [31]. Noise re-emerges as oscillations dephase again with further increases in amplitude, up to the maximum of 180 mV at 100 ms, marking the end of the ramp-up and start of the deramped phase.

For the deramped phase, the broadband emissions with prominent lines at $nf_0/4$, including subharmonics and harmonics at $nf_0/2$ and $nf_0$, persist until approximately 131 ms (152 mV). Continuing in the deramped phase, the clearing of broadband noise, indicating synchronous bubble oscillations (underpinned by the numerical result, Fig. 5b,f,h), is maintained for $\sim$ 50 ms. During 180-186 ms, increased broadband noise can be observed, while after 186 ms, the frequency of the emission signal localizes around the fundamental frequency and its harmonics.

A comparison of different time points in the spectrogram at identical excitation amplitudes confirms the presence of hysteresis. For example, at 135 mV, the emission during the ramp (50 ms) exhibits strong broadband noise, whereas during the deramp (150 ms) it is dominated by periodic harmonic lines. Similarly, at 108 mV, the ramped phase (20 ms) shows harmonic and subharmonic components, while the corresponding deramp (180 ms) displays increased broadband noise. These asymmetric spectral responses at the same excitation amplitudes in Fig. 2b (and the spectrograms in Online Resource 6) demonstrate that once a change in excitation alters the acoustic state, simply reversing the change does not restore the original condition – providing direct experimental evidence of dynamic hysteresis in cavitation emissions. We note that the main features of hysteresis discussed in this study remain consistent across all measurements, with a selection presented in Online Resource 6. Nonetheless, there are some variations in the feature details for each spectrogram obtained from each experimental run. We attribute these variations to the lack of control of the initial condition of cavitation within the tube transducer bore. This can influence how the cavitation field develops during each sonication, and the variation it results in is especially visible in the vicinity of bifurcation points, which is further explained in Sect. 4.3.

Notably, the behavior at 135 mV (50 and 150 ms) differs from the findings of Frohly et al. and Seya et al. [32, 33], who reported broadband features as more prominent during a series of sonications at decreasing intensities, than for an increasing intensity series. For a direct comparison with these works, we have also gathered data from our experiment for fixed-amplitude sonications, as part of increasing and decreasing intensity series, Online Resource 1. These results broadly agree with those reported by Seya et al. and Frohly et al.. The modeling in the following sections focuses on simulating the evolution of the intricate spectral features of the spectrogram (Fig. 2) recorded from cavitation under the continuous ramped-then-deramped sonication.

## Theoretical methods

In 2014, Hegedűs presented a theoretical demonstration of hysteresis in one-bubble dynamics [37], showing that under an increasing intensity series, bubble responses were attracted to a low-amplitude solution, whereas under a decreasing intensity series, they were attracted to a high-amplitude solution. As described in *Introduction* (Sect. 1), Seya et al. also numerically demonstrated hysteresis by reducing the multi-bubble system to a single bubble [33].

The present work extends these investigations in several directions. First, a fully coupled multi-bubble model with experimentally informed spatial distribution (see also Online Resource 2), is employed to capture bubble-bubble interactions. This is important because both experimental and numerical results in [31] indicate that individual, independent bubble oscillations are crucial to accurately reproduce the cavitation noise spectrogram. Second, spectrogram analysis is performed to characterize the temporal evolution of acoustic emissions. Third, continuous ramped-then-deramped excitation is introduced, allowing comparison of hysteresis behavior from the model with experimental observations.

**Fig. 3:**
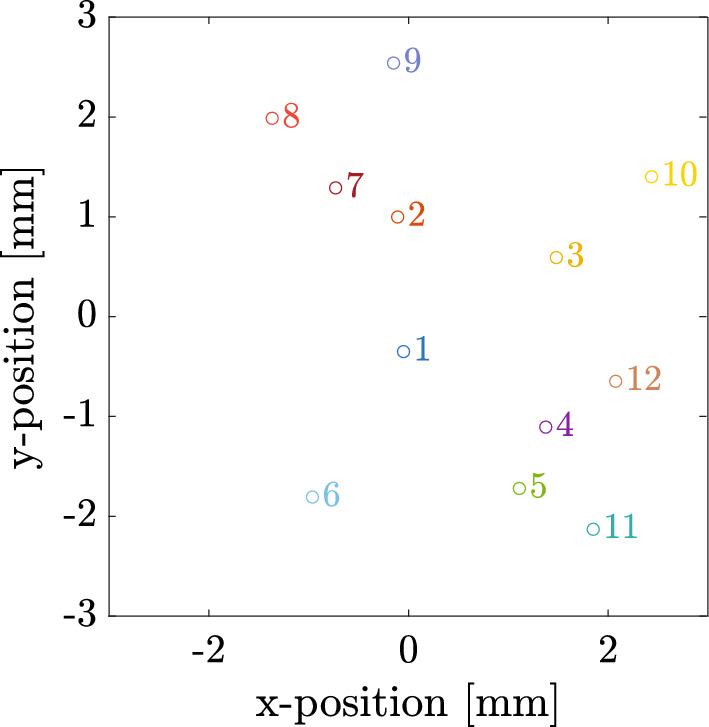
The bubble location setup of the 12-bubble system

In the present study, a 12-bubble model, shown in Fig. 3, is employed to investigate hysteresis behavior in spectral features. Originally introduced in [31], this model has been demonstrated to provide an accurate representation of the local bubble structure and to reproduce key spectral features observed in experiments, including subharmonic responses and broadband noise induced by asynchronous oscillations. For completeness, a concise overview of the governing equations is provided below, while a detailed derivation and parameter selection of the modeling approach is provided in [31] and conference proceeding [38]. Validation against experimentally detected emissions and an assessment of generality across alternative spatial distributions are presented later (Sect. 4.1 and Online Resource 2).

Equation (1) describes the dependence of each bubble’s radial oscillation on the local pressure field it experiences. Since 12 bubbles are modeled, 12 s-order ordinary differential equations (ODEs) are solved simultaneously:

1 $$\begin{aligned} R_i \ddot{R}_i + \frac{3}{2} \dot{R}_i^2  &   = \left( 1 + \frac{R_i}{c} \frac{d}{dt} \right) \left( \frac{P_{Li} - P_{\text {amb}} - P_A}{\rho } \right) \nonumber \\  &   \quad - \frac{1}{\rho } \sum _{j=1, \, j \ne i}^{N_b} P_{bj} \end{aligned}$$

Here, the subscript *i* (or *j*) denotes the *i*-th (or *j*-th) bubble in the multi-bubble system, *R* is bubble radius, $\rho$ = 998 kg/m$^3$ is the water density, $P_L$ is the liquid pressure acting on the bubble wall by the surrounding liquid outside the bubble, $P_{amb}$ = 101 kPa is the ambient pressure, *c* = 1484 m/s is the speed of sound in water. $P_b=\rho (2R{\dot{R}}^2+\ddot{R}R^2)/(C_pr)$ is the pressure contribution caused by the radial oscillations of all other bubbles, where $C_p$ is determined by a linear regression of Kirkwood-Bethe model, and *r* is the distance from the bubble center. $P_A$ is the acoustic excitation pressure, which is given by (2):

2 $$\begin{aligned} P_A = -p_{\text {amp}} \sin (2\pi f_0 t) \end{aligned}$$

where *t* is time, $f_0$ =15.5 kHz is the excitation frequency in the experiment and $p_{\text {amp}}$ is the excitation pressure amplitude. The liquid pressure at the bubble wall $P_L$ can be written as:

3 $$\begin{aligned} p_{L}=p_{g}-\frac{2\sigma }{R}-\frac{4\mu \dot{R}}{R} \end{aligned}$$

where $\sigma$ = 0.072 N/m is the surface tension coefficient, $\mu$ = 0.001 Pa$\cdot$s is the dynamic viscosity of water and $p_g$ is the gas pressure inside the bubble, which can be expressed by (4) with adiabatic law:

4 $$\begin{aligned} p_{g}=\left( p_{\text {amb}}+\frac{2\sigma }{R_{0}}\right) \left( \frac{R_{0}^3}{R^3}\right) ^{\kappa } \end{aligned}$$

where $R_0$ = 53.25 $\upmu$m is the equilibrium bubble radius and $\kappa$ = 4/3 is the polytropic exponent of air. This selection of equilibrium bubble radius results in maximum radii of the same order as those observed experimentally according to [31].

**Fig. 4:**
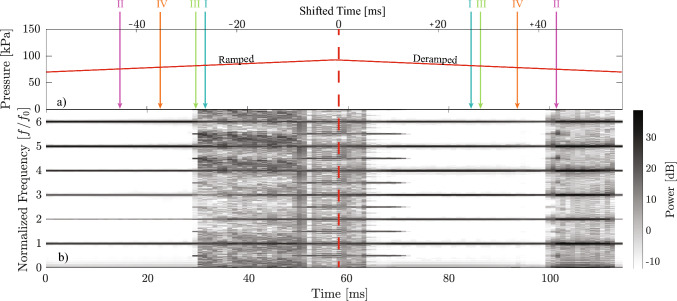
**a** The acoustic excitation pressure amplitude in a duration of 114.4 ms. The bold red lines represent the amplitude ramp from 70 kPa to 93 kPa and the symmetric deramp. The vertical red dashed line, i.e., the symmetry fold of the excitation amplitude, denotes $t_s = 0$ in the shifted time scale $t_s = t - 57.2$ ms, where negative and positive values indicate the ramped and deramped phases, respectively. Case I-IV are marked with cyan, purple, light green, and orange arrows. **b** Numerical spectrogram of the 12-bubble system, with the red dashed line again marking $t_s = 0$

## Numerical results

The temporal state of each bubble is characterized by the bubble radius and wall velocity. Therefore, 24 degrees of freedom are included in equation (1), which was solved numerically in MATLAB 2023a using the Runge–Kutta Dormand-Prince 45 method (ode45), with absolute and relative error tolerances set to 10$^{-9}$. All other integrator parameters were maintained at their default MATLAB values. The computations were conducted on a dual-socket AMD EPYC 7352 system with 96 logical processors at 2.3 GHz, supported by 503 GiB of RAM, of which 486 GiB was available during the simulations. The workload was GPU-accelerated using an NVIDIA Quadro RTX 4000 with 8 GiB of memory. The NUMA architecture of the system consists of two nodes, with 96 logical processors distributed. The simulation under the ramped-then-deramped excitation amplitude, Sect. 4.1, required approximately 1 CPU hour to complete, and the basin of attraction analysis in Sect. 4.4 required a total computational time of approximately 64512 CPU hours for 12 bubbles in a single scenario.

### Ramped-then-deramped excitation amplitude

The present study invesigated hysteresis under a ramped-then-deramped excitation amplitude. Specifically, the excitation pressure applied to the 12-bubble system is linearly increased from 70 kPa to 93 kPa and then symmetrically decreased, as shown in Fig. 4a. Like Fig. 2a, the amplitude envelope is shown in bold red, and the midpoint of the excitation cycle is indicated by a vertical red dashed line. Points equidistant from this dashed line correspond to the same instantaneous amplitude, enabling direct comparison of responses on the ramp and deramp. To formalize this symmetry, we introduce the shifted time variable $t_s = t - 57.2$ ms, such that the red dashed line corresponds to $t_s = 0$ on the upper axis of Fig. 4a. A schematic representation of the protocol is embedded in Fig. 4a: the excitation envelope rises to a peak value and then decreases symmetrically, clarifying how responses at equal amplitudes can be compared using the folded time axis. In this notation, negative values of $t_s$ correspond to the ramped phase, and positive values correspond to the deramped phase.

The corresponding numerical spectrogram is presented in Fig. 4b, where the red dashed line, extended from Fig. 4a, indicates the transition from the ramped to the deramped phase. The numerical spectrogram, Fig. 4b, shows remarkable qualitative agreement with the experimental observation in Fig. 2b, particularly in the occurrence of broadband emissions and the subsequent clearing. During the initial 29 ms, the system exhibits strong harmonic and subharmonic emission lines at integer multiples, *n*, of the fundamental frequency $f_0$ = 15.5 kHz, indicating a stable and periodic oscillatory regime. Here, *stable* denotes dynamical stability of attractors, distinct from the term *stable cavitation* used to describe non-inertial bubble oscillations. Between 29 and 51 ms, a broadband noise region is preceded by $nf_0/2$ spectral lines, in agreement with the subharmonic route to acoustic chaos [30]. At approximately $t_s = -6.2$ ms, clearing of the broadband noise appears, accompanied by the appearance of subharmonic features at $nf_0/2$ and $nf_0/4$. After this clearing of broadband emission, noise re-emerges at around $t_s = -4.2$ ms. The initial harmonics, the subharmonic route to acoustic chaos, the reverse bifurcation, and the re-emergence of broadband noise confirm our experimental observations in Fig. 2b. It is worth noting that the numerical spectrogram in Fig. 4b spans 57.2 ms of the ramped phase, compared to 100 ms in Fig. 2b. A longer ramp starting from $t_s = -100$ ms (corresponding to a pressure amplitude of 52.8 kPa) leads to a spectrogram dominated by $nf_0$ emissions at excitation amplitudes below 78 kPa. These are not the main focus for this study; therefore, 70 kPa is selected as the starting pressure amplitude, to skip most of this regime and focus on the more interesting dynamics such as the route to chaos and broadband noise clearing. Similarly, to capture the key spectral features and optimize computational resources, the excitation ramp is stopped at 93 kPa after observing the re-emergence of broadband noise, as seen experimentally, in Fig. 2b.

**Fig. 5:**
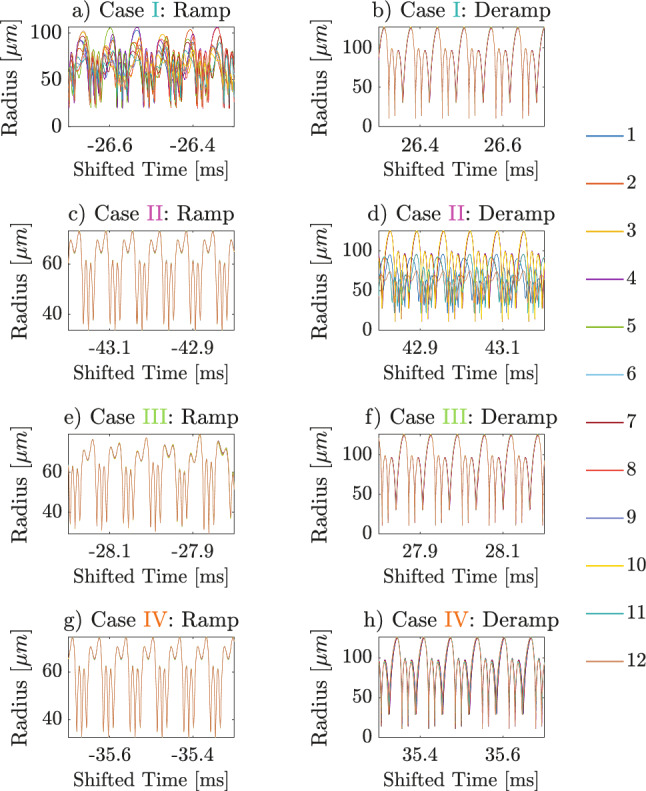
Radial oscillations of the 12 bubbles during eight 0.4 ms time windows in four hysteresis cases under the shifted time scale. The left column shows the ramped phase, while the right shows the deramped phase. The corresponding frequency contents of the cases are indicated by the colored arrows in Fig. 4

**Fig. 6:**
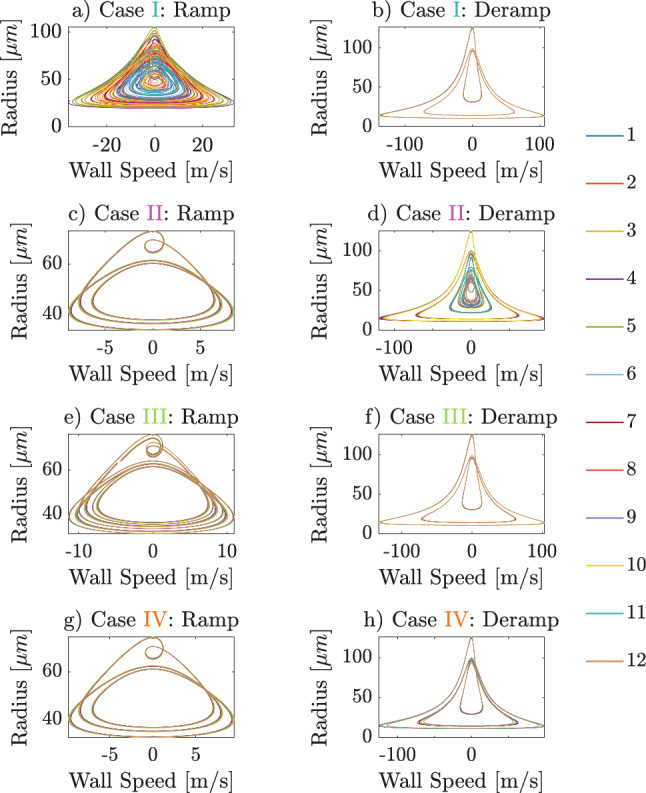
Phase trajectories in the same four hysteresis cases as Figs. 4 and 5

**Fig. 7:**
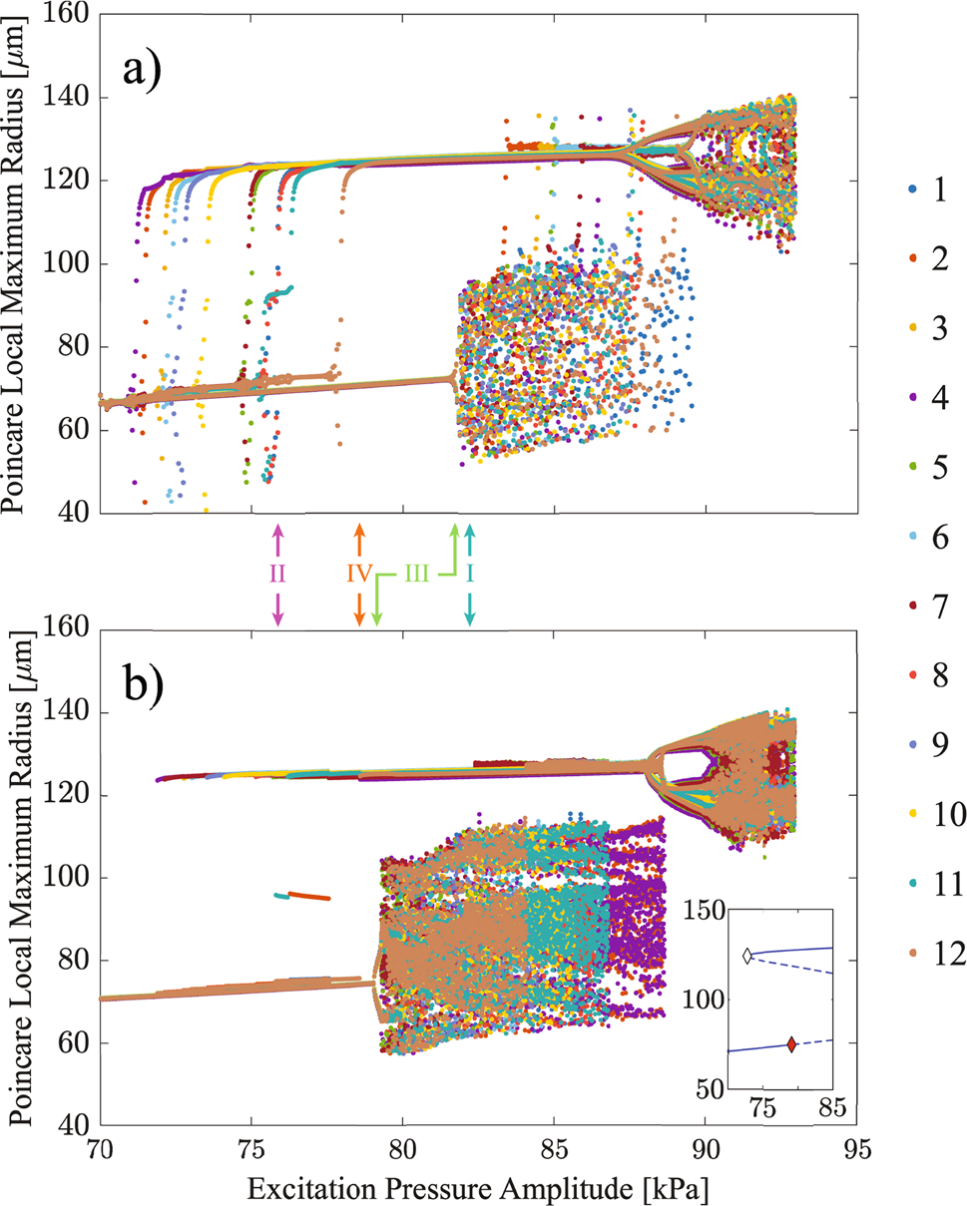
**a** Poincaré mapping of the system under ramped-then-deramped excitation amplitude **b** Bifurcation diagram generated from increased-then-decreased series of 1758 excitation amplitudes. The four hysteresis cases illustrated in Figs. 5 and 6 lie within the excitation range of 70-85 kPa, indicated by the arrow-headed line along the x-axis. Insert: Bifurcation diagram obtained via boundary value problem using the COCO continuation software. The x- and y-axes share the same labels and units as Fig. 7b. The solid and dashed curves are the stable and unstable solutions, respectively. Saddle-node and period-doubling bifurcations are marked by white and red diamonds, respectively. The legend on the right denotes 12 bubbles, and the points represent sampled bubble radii on the Poincaré sections

**Fig. 8:**
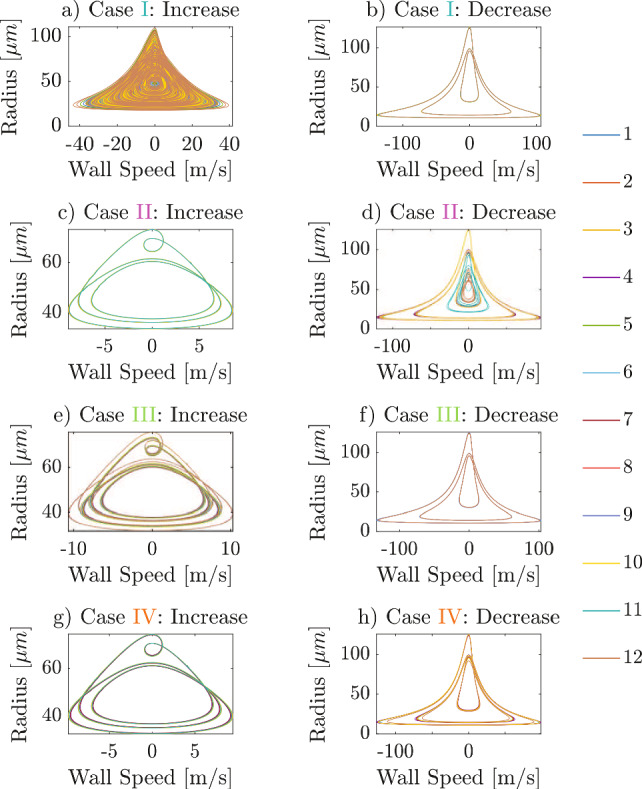
Steady-state trajectories of the 12 bubbles for the identical four hysteresis cases in Fig. 7b

In the deramped phase, the spectral features clearly demonstrate hysteresis, specifically, four representative cases illustrate this behavior. From the full simulation time of 114.4 ms, the cases are trimmed into time windows of 0.4 ms to investigate the local frequency content (here) and temporal response (in the next section). In Case I, the spectrogram shows broadband noise at $t_s = -\,26.5$ ms (ramped phase), whereas at $t_s = +26.5$ ms (deramped phase) $nf_0$ spectral lines are shown. In Case II, the emission remains at $nf_0$ at $t_s = -43$ ms during the ramped phase but shifts to broadband noise at $t_s = +43$ ms. In Case III, $nf_0/2$ spectral lines are observed at $t_s = -27$ ms, while at $t_s = +27$ ms the emission remains at $nf_0$ in the deramped phase. Case I-III in Fig. 4b show that the system exhibits different frequency contents at the same excitation amplitude, depending on whether the excitation is ramped or deramped. As will be shown in the next section, this difference in frequency contents highlights more profound differences in the system response. In Case IV, the frequency content at $t_s = \pm 35.5$ ms appears to be dominated by identical $nf_0$ components, nonetheless, the amplitude of the responses differs as further described in the next section. In Fig. 4, the four cases are highlighted in cyan, purple, light green, and orange, and the same color scheme is applied to the case numbers in Table 1 and Figs. 4, 5, 6, 7 and 8. These cases, summarized in Table 1 and analyzed in detail in Sect. 4.2, illustrate distinct forms of hysteresis in the response.

In Fig. 4b, the broadband noise is cleared after the initial 4.2 ms of the deramped phase. Then at around 99 ms, the noise re-emerges with a broadband region of 13 ms. After 112 ms, the emission is cleared again. Like the ramped phase, the deramped phase of the numerical result in Fig. 4b also demonstrates agreement in broadband emission and clearing with the experimental spectrogram in Fig. 2b. It is important to note that the experimental signal collected by the needle hydrophone was the combined pressure signal from all the bubbles in the field; in this paper, a small subset of 12 bubbles is modeled. However, the similarity between the experimental spectrogram and the numerical one validates the model and demonstrates that the hysteresis phenomenon shown in numerical results underpins the memory-dependent behavior in the spectrogram observed experimentally. In addition, spectrogram results for different bubble distributions are presented in Online Resource 2, supporting the generality of hysteresis observation across multi-bubble configurations.

**Table 1:** Summary of the four representative cases illustrating hysteresis, showing the responses at symmetric time windows in the vicinity of $t_s$ during the ramped and deramped phases

Case	$t_s$ (ms)	Response
	$-26.5$	Broadband noise
	$+26.5$	$nf_0$ spectral lines
	$-43$	$nf_0$ spectral lines
	$+43$	Broadband noise
	$-27$	$nf_0/2$ spectral lines
	$+27$	$nf_0$ spectral lines
	$-35.5$	$nf_0$ spectral lines (low amplitude)
	$+35.5$	$nf_0$ spectral lines (high amplitude)

The case numbers are color-coded (cyan, purple, light green, and orange) consistently with their representation in Fig. 4 and carried through Figs. 5, 6, 7 and 8

### Time history and phase trajectory

The radial oscillations of the 12 bubbles corresponding to the four cases, where hysteresis behavior is identified, are presented in Fig. 5. In each case, the left and right panels in Fig. 5 present the time history at identical excitation amplitude.

The presence or absence of broadband noise observed in the spectrogram, Fig. 4b, can be related to the degree of bubble synchronization in Fig. 5. In Case I, the left panel of Fig. 5 exhibits desynchronized oscillations of the 12 bubbles, while the right panel shows synchronization. This corresponds to the cyan arrow in Fig. 4b, where the ramped phase of this hysteresis region is characterized by broadband noise, and the deramped phase is acoustically cleared. Conversely, Case II reveals synchronized period-1 oscillations on the left and desynchronization on the right, corresponding to harmonics in the ramped phase and broadband noise on the deramped phase in the spectrogram, Fig. 4b, respectively. Case III is shown in Fig. 5e–f. In Fig. 5e, during the ramped phase, the system is on a period-doubling route to chaos, while in Fig. 5f, in the deramped phase, a period-1 solution is obtained. Case IV represents another hysteresis behavior in time history, although the frequency content seems to be the same, as highlighted by orange arrows in the spectrogram, Fig. 4. In this case, presented in Fig. 5g–h, both panels show period-1 bubble oscillations; however, the maximum radius of the left panel is higher than that of the right. These findings suggest that hysteresis in this system can also cause the response to converge to attractors with identical frequency content but with different oscillation amplitudes. To provide a clearer visualization of the convergence of the response, Fig. 6g–h presents the shape of phase-space trajectories in Case IV. The trajectory in the left panel appears to be attracted to a low-amplitude solution, while the trajectory in the right panel converges to a high-amplitude one.

Phase space trajectories of Case I-III are also presented in Fig. 6. In case I and II, although both Fig. 6a and d correspond to the broadband noise region (the cyan arrow in the ramped phase and the purple arrow in the deramped phase) in the spectrogram, Fig. 4b, they exhibit notable differences in oscillation periodicity. Case I during the ramped phase, Fig. 6a, displays chaotic trajectories, whereas in Case II in the deramped phase, Fig. 6d, all bubbles exhibit quasi-periodic behavior: small differences in the response over a period does not allow the trajectories to close perfectly on themselves. It is argued that this is due to the transient behavior induced by the continuously changing nature of the excitation pattern. Fig. 6b,f converges to the high-amplitude attractor identical to Fig. 6h, while Fig. 6c converges to the same low-amplitude attractor as Fig. 6g. Figure 6e, corresponding to period doubling at the onset of acoustic chaos in Fig. 4b, reveals that the phase trajectory seems to converge to a period-2 attractor which doubles the loops compared with Fig. 6c,g. However, these observations require further verification through steady-state analysis to identify stable attractors of those trajectories directly, described in the next section.

### Poincaré map and steady-state analysis

A possible approach to obtain the attractors that correspond to different amplitudes of excitation is to use an initial value problem (IVP) solver where the excitation is ramped to the desired value and kept constant until the system reaches steady-state. Unlike the ramped-then-deramped simulation, where the excitation amplitude changes continuously and the transients are retained, in the steady-state analysis the excitation amplitude is increased-then-decreased in steps, i.e. once a desired amplitude level is reached, it is kept fixed to allow transients to fade out and enable the system response to converge to a stable attractor. Poincaré sections are used to study the dynamics of the system in both the continuously ramped-then-deramped case and in the steady-state setup: the system response is sampled once every excitation period. In Fig. 7, each color-coded point represents a sampled value on the Poincaré section. The twelve distinct colors correspond to the twelve bubbles in the system, as indicated by the legend in Fig. 7. Figure 7a shows the result of the continuously ramped-then-deramped simulation; and panel (b) shows the steady-state response of the system for an increased-then-decreased excitation discretized in 1758 steps. The Poincaré map allows the continuous three-dimensional dynamics to be captured in a two-dimensional discrete map, enabling a clearer visualization of both periodic (where Poincaré sections converge) and aperiodic or strange attractors (where Poincaré sections scatter).

The four hysteresis cases shown in Figs. 5 and 6 are marked by arrows along the x-axis of Fig. 7. Comparing panel (a) and panel (b) in Fig. 7, the type of responses in Cases I and IV are the same at identical amplitude levels, in both the continuously ramped-then-deramped simulation and the steady-state analysis: Case I exhibits the coexistence of a periodic orbit and a strange attractor, while Case IV shows the coexistence of low- and high-amplitude attractors.

However, there are clear differences for Case II and III. This is due to the proximity to bifurcation points, as shown in the insert of Fig. 7b. The bifurcation point can be examined for individual bubbles within the system (see Online Resource 3). To examine the bifurcation points, a bifurcation diagram is obtained by solving the system as a boundary value problem (BVP) with the pseudo-arclength continuation technique, using the COCO software package [39], to characterise the stability of period-1 solutions of the single bubble system in the excitation range 70-85 kPa. The periodic solutions are computed independently of their stability, using orthogonal collocation with three collocation points per mesh interval. To resolve the sharp features and fast dynamics around bubble collapse (evident from the steep variations in wall velocity and narrow radius minima in Fig. 8b,d,f,h), each oscillation period is discretized with 800 mesh intervals. The mesh is adaptively refined to maintain a local discretization error with a relative tolerance of 10$^{-8}$. Stable and unstable attractors are identified, with saddle-node and period-doubling bifurcation points marked by white and red diamonds, respectively. This analysis confirms the coexistence of multiple attractors at identical excitation amplitudes and highlights the bifurcation points that delimit their regions of stability. In the vicinity of these bifurcation points, the effect of the transient response on the Poincaré maps is exacerbated – this results in substantial differences between the maps in panels (a) and (b) at the excitation amplitude levels corresponding to Case II and III.

In Case II, the ramped-then-deramped excitation in panel (a) produces scattered responses, indicating desynchronization, whereas the steady-state analysis in panel (b) clearly identifies low-, mid-, and high-amplitude solutions. The transient dynamics tend to destabilize the mid- and high-amplitude attractor, driving the system to the lower-amplitude branch, which appears to be more stable in the interval 71.0 to 78.0 kPa. This observation will be further investigated in the next section through the basin of attraction of coexisting responses. The transient effects also induce desynchronization among bubbles, giving rise to broadband noise. This mechanism accounts for the noise segment observed between 99-112 ms in Fig. 4b and between 180-186 ms in Fig. 2b. The example illustrates that transient effects cannot be neglected in the neighborhood of bifurcation points, where attractors may change stability, or new attractors may emerge. As a bifurcation is approached, the basin of attraction of a stable solution shrinks and eventually vanishes, making the solution increasingly sensitive to external disturbances–in this case the change in excitation amplitude during the ramped-then-deramped simulation.

In Case III, the system follows the pathway to chaos through period-doubling bifurcations. Chaotic responses are observed in the region between 82.0 and 90.0 kPa for Fig. 7a and 79.5 and 88.8 kPa for Fig. 7b. This shows that the chaotic region is more extended in the steady-state analysis than in the ramped-then-deramped simulation, and it also appears at higher amplitudes in panel (a). For this reason, a sensible comparison between panel (a) and (b) for Case III requires different excitation levels: 81.7 kPa in panel (a) and 79.2 kPa in panel (b).

For completeness, the steady-state phase trajectories are shown in Fig. 8, which reveal the underlying attractor shapes. All four cases exhibit dynamic hysteresis even under steady-state conditions, with the left and right columns corresponding to the increasing and decreasing series of amplitudes, respectively.

In Case I, Fig. 8a exhibits open orbits, which are characteristic of strange attractors, whereas Fig. 8b shows closed trajectories with maximum bubble radii of 124 $\mu$m, indicating periodic high-amplitude orbits. In Case II, Fig. 8c displays periodic low-amplitude orbits for all the bubbles, while Fig. 8d reveals that different bubbles are attracted to different attractors, namely low-, mid- and high-amplitude attractors: for example, the response of bubble 11 (light blue curve in Fig. 8d) converges to the mid-amplitude attractor. This phenomenon, which involves different bubbles being drawn to distinct attractors in Case II, is further explored in the next section. In Case III, Fig. 8e shows closed orbits with two loops per cycle, corresponding to a period-2 attractor, while Fig. 8f exhibits high-amplitude period-1 orbits—this applies to all bubbles. Finally, in Case IV, Fig. 8g shows convergence to low-amplitude attractors, whereas Fig. 8h shows high-amplitude attractors for all bubbles. Consistent with the earlier observation from Fig. 6a,d, where Case I showed chaotic trajectories and Case II quasi-periodic ones, Fig. 8a,d demonstrates that, after the attenuation of transients in steady-state analysis, the ramped phase of Case I corresponds to chaotic attractors, while the deramped phase of Case II follows periodic orbits. Overall, Fig. 8 confirms that even at identical excitation amplitudes, responses can converge to different attractor shapes, establishing hysteresis as a consequence of coexisting attractors.

### Basin of attraction

Due to the memory-dependent nature of hysteresis, different initial conditions can lead the system to converge to different attractors, even under identical excitation conditions. The basin of attraction is defined as the set of initial conditions from which the trajectories converge to a given attractor [40, 41]. In this section, two-dimensional slices of the basin of attraction are examined to illustrate how initial conditions and bubble-bubble interactions influence the collective response of the multi-bubble system.

The full basin of attraction exists in a 24-dimensional space, corresponding to the 2 degrees of freedom for each of the 12 bubbles. To make the problem tractable, only the initial radius and wall speed of a single bubble—the *control bubble*—are varied, while all other bubbles are assigned identical initial conditions. Unless otherwise stated, bubble 2 is chosen as the control bubble. The corresponding results for all other choices of the control bubble are presented in Online Resource 4.

**Fig. 9:**
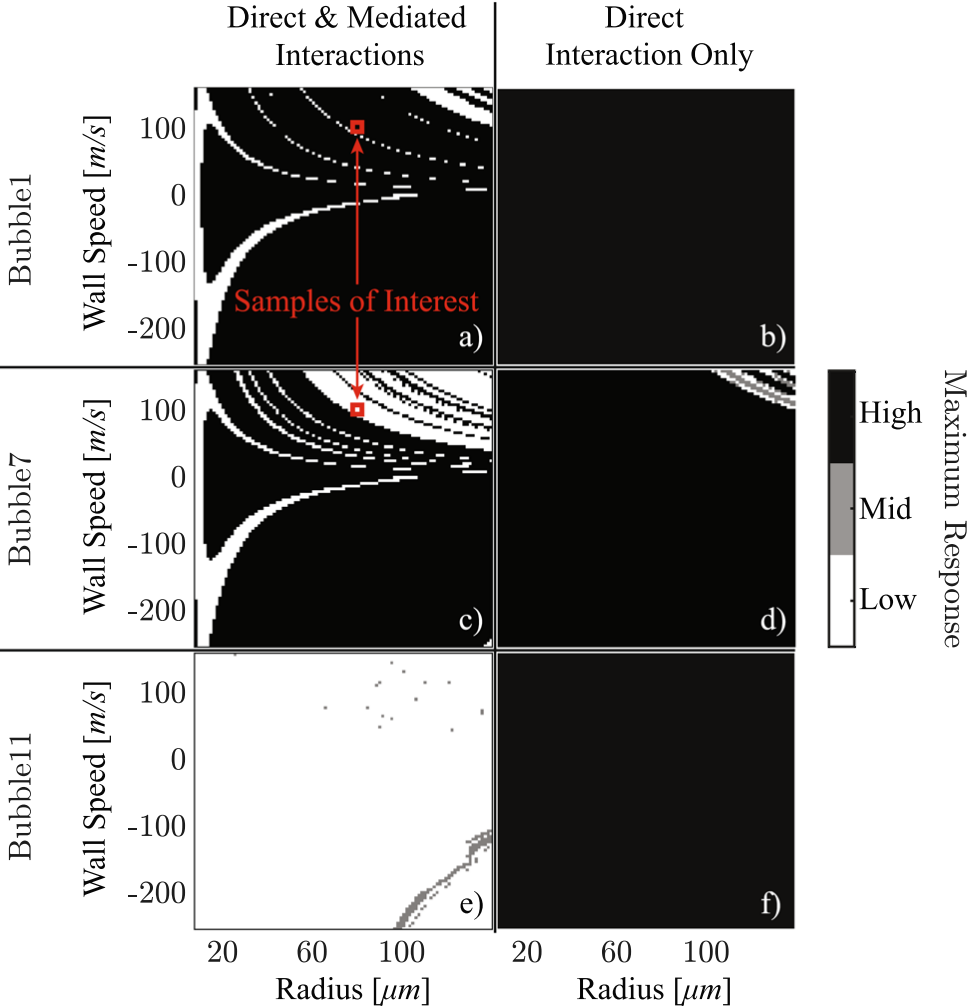
By controlling the initial condition of bubble 2, the maximum steady-state response is plotted to obtain: **a**–**f** the amplitude basins for bubble 1, 7, and 11, where white, gray, and black regions indicate low-, mid-, and high-amplitude attractors, respectively. The left column shows results implementing interactions between all bubbles, while the right column are results neglecting interactions among the non-control bubbles. The red squares in (**a**) and (**c**) mark the responses of bubble 1 and bubble 7, respectively, for an initial radius of 80 $\upmu$m and a wall speed of 100 m/s of the control bubble

All basins of attraction are shown as two-dimensional grayscale plots, where the x-axis represents the initial radius and the y-axis represents the initial wall speed. Each point in the plot is shaded according to the attractor reached from those initial conditions [42, 43]. With the exception of the control bubble, identical initial conditions are assigned to all the other bubbles, and the convergence of each bubble’s response is monitored. As an example, Fig. 9a shows the basins of attraction of bubble 1 when the initial conditions of bubble 2 are varied. Attractors are classified according to the maximum steady-state amplitude of bubble 1 and the color bar of Fig. 9 distinguishes low-, mid- and high-amplitude attractors. In Fig. 9a, only low- (white) and high-amplitude (black) attractors appear. Figures of this type, Fig. 9a–f, are referred to here as *amplitude basins*.

As shown in Fig. 9a and c, bubble 1 and bubble 7 exhibit different amplitude basins, indicating that even under identical initial conditions of the control bubble, different bubbles can converge to different attractors depending on their relative position. This arises because the effective coupling strength between bubbles depends on distance, so the influence of the control bubble is not uniform across the system. For example, when the control bubble has an initial radius of 80 $\upmu$m and a wall speed of 100 m/s (red squares in Fig. 9a and c), bubble 1 converges to a high-amplitude attractor, while bubble 7 converges to a low-amplitude attractor. Figure 9e shows the amplitude basin of bubble 11, where a mid-amplitude (gray) attractor also emerges. This mid-amplitude attractor is consistent with the light-blue branch near 95 $\upmu$m in Fig. 7b.

**Fig. 10:**
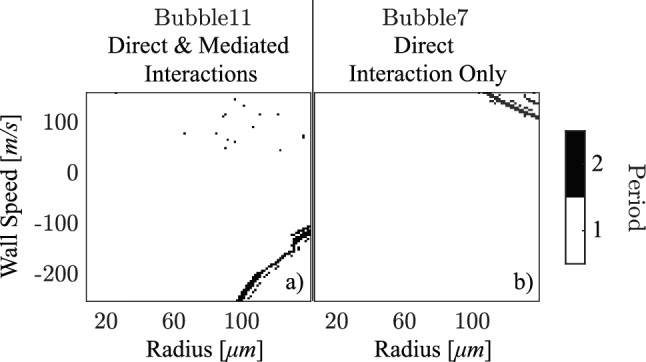
The period of bubble oscillation is plotted to obtain the period basins of **a** bubble 11 and **b** bubble 7, which are the corresponding period basins of Fig. 9**e** and d, respectively. Like Fig. 9, the left column, Fig. 10a, shows results implementing interactions between all bubbles, while the right column, Fig. 10b, are results neglecting interactions among the non-control bubbles

As discussed in Sect. 4.3, the attractors can also differ in the period of oscillation. Figure 10 presents the basins of attraction of bubble 7 and bubble 11, categorized by the steady-state oscillation period. The basins of attraction in Fig. 10 are therefore referred to as *period basins*. Period basins are not shown for bubble 1 and bubble 7 since their responses are always period-1 and thus trivial.

Figures 9a,c,e and 10a demonstrate that, even with identical initial conditions for the control bubble, different bubbles within the system can converge to distinct attractors in both amplitude and periodicity (see Online Resource 4 for the basins of attraction of the other bubbles). This observation suggests the additional role of bubble-bubble interactions. Because of the spatial distribution, the strength of interaction varies between bubbles, and it is useful to distinguish between direct and mediated interactions. In the direct interaction, the control bubble influences the observed bubble directly; in the mediated interaction, the control bubble first influences another bubble, which then influences the observed one.

The study of bubble-bubble interactions is therefore divided into two parts: the first considers global interactions among all bubbles (including both direct and mediated interactions) modulated by a scaling factor. The second isolates the direct interaction by neglecting couplings among the non-control bubbles.

With a scaling factor of 100, all bubble-bubble interactions are negligible because the bubbles are positioned one hundred times further apart than in their original configuration (for results with scaling factors of 100 and 0.1, see Online Resource 5). Under this condition, all bubbles, except the control bubble, exhibit identical amplitude and period basins, corresponding to a high-amplitude, period-1 attractor. This trivial case results in amplitude basins which are uniformly black (high amplitude) and period basins which are uniformly white (period 1) – for more detail see Online Resource 5.

When only direct interactions with the control bubble are considered and the scaling factor is restored to 1, Fig. 9b and f show that bubble 1 and 11 converge to the high-amplitude attractor. Their period basins—not shown in Fig. 10 as they reduce to all white—confirm that these attractors are period-1. In fact, every bubble, except bubble 7, converges to the high-amplitude and period-1 attractor as shown in Online Resource 5. However, as illustrated in Figs. 9d and 10b, bubble 7 can converge to a low-amplitude period-1 attractor, a high-amplitude period-1 attractor, or a mid-amplitude period-2 attractor. Since bubble *m* ($m \ne 2,7$) is located farther from bubble 2 than bubble 7, as shown in Fig. 3, its interaction with the control bubble is weaker. Consequently, the high-amplitude period-1 attractor dominates the basin of attraction for all other bubbles, similar to the weak interaction case resulting from a scaling factor of 100.

## Discussion and conclusion

The experimental and numerical spectrograms of this study reveal clear hysteresis in the cavitation emissions during a continuously ramped-then-deramped sonication. This is demonstrated by the asymmetry in the experimental and numerical spectrograms. To make a quantitative summary of the core findings in these spectrograms, the broadband component is represented as the percentage of power contained in non-harmonic frequencies. To account for spectral leakage, a comb-notch filter with the notch of 750 Hz was applied over the full bandwidth of 100.75 kHz shown in the spectrograms, Fig. 2b and Fig. 4b. The experimental spectrogram in Fig. 2b reveals an asymmetry at matched excitation levels in broadband noise between 50 ms (61%) and 150 ms (9%), as well as between 20 ms (5%) and 180 ms (20%). A similar asymmetry is shown in the theoretical spectrogram. Fig. 4(b) indicates differences in broadband noise between $t_s$ = − 26.5 ms (58%) and $t_s$ = 26.5 ms (5%), and between $t_s$ = -43 ms (2%) and $t_s$ = 43 ms (35%). The broadband asymmetry observed in the numerical spectrogram is also presented in polydisperse systems, as demonstrated in Online Resource 7. Time-history analysis shows that periodic, synchronous bubble oscillations generate harmonic and subharmonic components, whereas aperiodic, asynchronous oscillations correspond to broadband noise. Phase-space representations identify four cases in which trajectories at identical excitation amplitudes differ between the ramped and deramped phases. The bifurcation analysis demonstrates that hysteresis arises from the history-dependent convergence to coexisting attractors, delimited by saddle-node and period-doubling bifurcations. The basin-of-attraction computations further show that the attractor reached at a given amplitude depends on the initial conditions and bubble-bubble interactions.

The studies by Frohly et al. [32] and Seya et al. [33] presented experimental spectra from a series of fixed-amplitude sonications with intervening off-intervals. They showed that spectra collected during a series of sonications of decreasing intensities exhibit more prominent inertial components than the spectra collected during a series of increasing intensities. In contrast, the spectrogram in Fig. 2b (and the spectrograms in Online Resource 6) indicates that broadband noise may be stronger during the ramped phase. However, the excitation protocol in those studies differs from the continuous ramped-then-deramped excitation applied here. To enable a direct comparison, we also analyzed our data using a series of sonications with off-intervals; under these conditions, our results broadly reproduce the trend reported in [32, 33] (Online Resource 1). This suggests that the discrepancy in hysteresis observations is at least partially attributable to differences in the applied excitation protocols. As described in the *Introduction*, the simulations of Seya et al. primarily attribute these observations to changes in equilibrium bubble radius, due to rectified diffusion. These changes were implemented manually, however, by adjusting the equilibrium radius after each sonication, in preparation for the next. Bubbles above and below preset values of radius were also removed, to represent buoyancy and dissolution effects, respectively. Moreover, this model greatly reduces the number of governing equations and incorporates stochastic elements, such as the introduction of a random number of daughter bubbles, to represent fragmentation events.

The hysteresis effects reported in this paper occur in response to a continuously varying ramped-then-deramped sonication. Applying manual adjustments to system parameters during the simulation would therefore significantly limit physical relevance. Instead, we employ a fully coupled deterministic multi-bubble system, which reproduces the detailed features of the experimental spectrogram, to a remarkable degree. This includes the emergence of subharmonics, the development and clearing of broadband noise, and the asymmetry between ramped and deramped phases. Even when the equilibrium radius is fixed, the present work shows that dynamic hysteresis can arise, and demonstrates the coexistence of multiple attractors at the same instantaneous excitation amplitude. The hysteresis observed under the continuous sonication therefore reflects transitions between these coexisting attractors, rather than changes in equilibrium bubble radius. This explains the observation of the stronger broadband noise observed during the ramp compared with the deramp. At lower excitation amplitudes, the bubbles continue converging toward the low-amplitude attractor during the ramp, whereas in the deramp they transition from high- to low-amplitude oscillations, producing transient noise. At higher excitation amplitudes, the bubbles follow the subharmonic route to chaos during the ramp, but settle into periodic attractors during the deramp.

Several other studies have recently adopted fully coupled multi-bubble models, but to the best of our knowledge, none have demonstrated hysteresis behavior in cavitation emissions. For example, Haghi et al. [44] demonstrated the influence of bubble-bubble interactions on bubble oscillations. However, their model used a spherical spreading approximation for bubble emissions, which has been shown to be inaccurate according to the Kirkwood-Bethe approximation [31, 45]. Importantly, their study lacks validation against hydrophone measurements of acoustic cavitation, leaving the predicted emission behavior unverified. Zhang et al. [46] developed a unified model for bubble oscillation and migration. The model [46] was restricted to short simulation durations (tens of milliseconds), as the Runge–Kutta integration diverges when two bubble centers approach one another and coalesce. Their work was also presented without experimental validation of the modeled acoustic cavitation emissions.

The fully coupled multi-bubble model used in this study reproduces the experimentally observed hysteresis with notable qualitative agreement, highlighting the importance of fully resolving bubble-bubble interactions. This level of agreement establishes a benchmark for a physically interpretable model in predicting experimentally generated cavitation emissions. However, several physical processes are not included, such as rectified diffusion, bubble fragmentation, coalescence, and migration. These mechanisms could still contribute to the hysteresis behavior through the continuous evolution of bubble size and position. Bubbles may migrate towards one another due to mutual attraction; alternatively, repulsion from nearby bubbles can drive them into contact with other neighbors. Thus, bubble migration can promote coalescence. Coalescence and rectified diffusion promote bubble growth, potentially shifting bubbles closer to the resonant radius ($\sim$200 $\mu$m for an excitation frequency of 15.5 kHz). In contrast, fragmentation reduces bubble size, moving bubbles away from resonance. Since oscillations are generally more violent near the resonant radius, acoustic emissions are expected to increase as bubbles approach resonance and decrease when they move further from it [12]. Additionally, the bubble population is limited to twelve due to the constraint of computational power. Phase transition, which is comprehensively studied in free oscillation systems such as laser cavitation [47, 48], is also not included in this study of acoustic cavitation because of the fundamental difference between rapid energy deposition-induced cavitation and acoustically driven cavitation, as highlighted in [45, 49]. A logical direction for future work is to investigate the influence of these mechanisms on the cavitation emissions, in a physically meaningful way. Further incorporation of such mechanisms, which exert significant dynamical influence, within the deterministic, fully coupled framework, would broaden the predictive capability of multi-bubble models. In addition, heat and mass transfer in the bubbles could be incorporated; for example, the numerical method proposed in [50] models the processes of heat and mass transfer while accounting for phase change and liquid compressibility.

Potential implications of this work lies in the application of ultrasonic cleaning. Specifically, amplitude modulation of the driving signal at 50 or 100 Hz is implemented in commercial ultrasonic cleaning devices, equipment, and ultrasonic reactors [51]. This is realized as a ‘double half-wave’ modulation, in which the acoustic pressure amplitude periodically ramps up and down, subjecting the cavitation field to continuously varying excitation conditions. Our findings identified higher levels of subharmonic and broadband emissions, which are recognized indicators of more aggressive cavitation activity, in the ramped phase (relative to the deramped phase). Under the aforementioned practical operating conditions, this may imply that enhanced cleaning may occur during the ramped phases, with inertial emissions associated with bubble activity such as jetting and shockwave emission, known to contribute significantly to cleaning effects [52–55]

Overall, the present work establishes that the hysteresis observed in cavitation emissions under continuous ramped-then-deramped excitation amplitude reflects the structure of the underlying nonlinear dynamics. The coexistence of multiple attractors, the bifurcations that delimit their stability, and the influence of initial conditions and bubble-bubble interactions, together produce a history-dependent response even when the equilibrium radius is fixed. These findings clarify the physical origin of cavitation hysteresis and may support future efforts to interpret cavitation spectra, or improve efficiency in ultrasonic cleaning.

## Supplementary Information

Below is the link to the electronic supplementary material.

Supplementary file 1 (pdf 1378 KB)

Supplementary file 2 (pdf 415 KB)

Supplementary file 3 (pdf 779 KB)

Supplementary file 4 (pdf 10364 KB)

Supplementary file 5 (pdf 1374 KB)

Supplementary file 6 (pdf 1257 KB)

Supplementary file 7 (pdf 733 KB)

## Data Availability

All data and materials supporting the findings of this study are available within the paper and its Supplementary Information.
