# Supplementary material for: Hysteresis in cavitation emissions during a ramped-then-deramped amplitude sonication: A theoretical and experimental investigation
Source: Nonlinear Dyn. 2026 Apr 21;114(8):591. doi: 10.1007/s11071-026-12462-3 (PMC13100018; doi:10.1007/s11071-026-12462-3)
Supplement: Supplementary file 1 — (pdf 1378 KB) [file 11071_2026_12462_MOESM1_ESM.pdf]

Hysteresis in cavitation emissions during a ramped-then-deramped amplitude sonication

A theoretical and experimental investigation

Supplementary Material 1: Fixed-amplitude Sonications

Y. Zhang[1], S. Li[1], P. Prentice[1] and A. Cammarano[2]
[1]Cavitation Laboratory, Centre for Medical and Industrial Ultrasonics,
University of Glasgow, University Avenue, Glasgow, G12 8QQ, UK
[2]Department of Aeronautics and Astronautics,
University of Southampton, Burgess Road, Southampton, SO16 7QF, UK
email: andrea.cammarano@soton.ac.uk

Journal: Nonlinear Dynamics

Our experiment and simulation used a ramped-then-deramped excitation amplitude (§2 *Experimental observations* and §3 *Theoretical methods*) rather than the increasing and decreasing series used by Frohly et al. [1] and Seya et al. [2]. The spectrograms of our experimental and numerical results (Fig. 2(b) and Fig. 4(b) in the main manuscript) show that lower broadband noise can be observed in the deramped phase than in the ramped phase, whereas Frohly et al. and Seya et al. reported stronger broadband and subharmonic emissions in the decreasing series. We speculate that the discrepancy may be at least partially due to differences in the sonication protocol. To support this, the series of increases and decreases in excitation amplitude is applied to induce interruptions between bursts of sonications, in accordance with the protocol used in [1, 2]. With an interruption of 500 ms between two successive sonications, each sonication lasted 5 s in the experiment and 300 ms in the simulation to reduce computational demand. Hysteresis is observed in both the experimental and numerical spectra, as shown in Fig. 1 and Fig. 2, where the blue and red curves represent the spectra of the ramped and deramped phases, respectively.

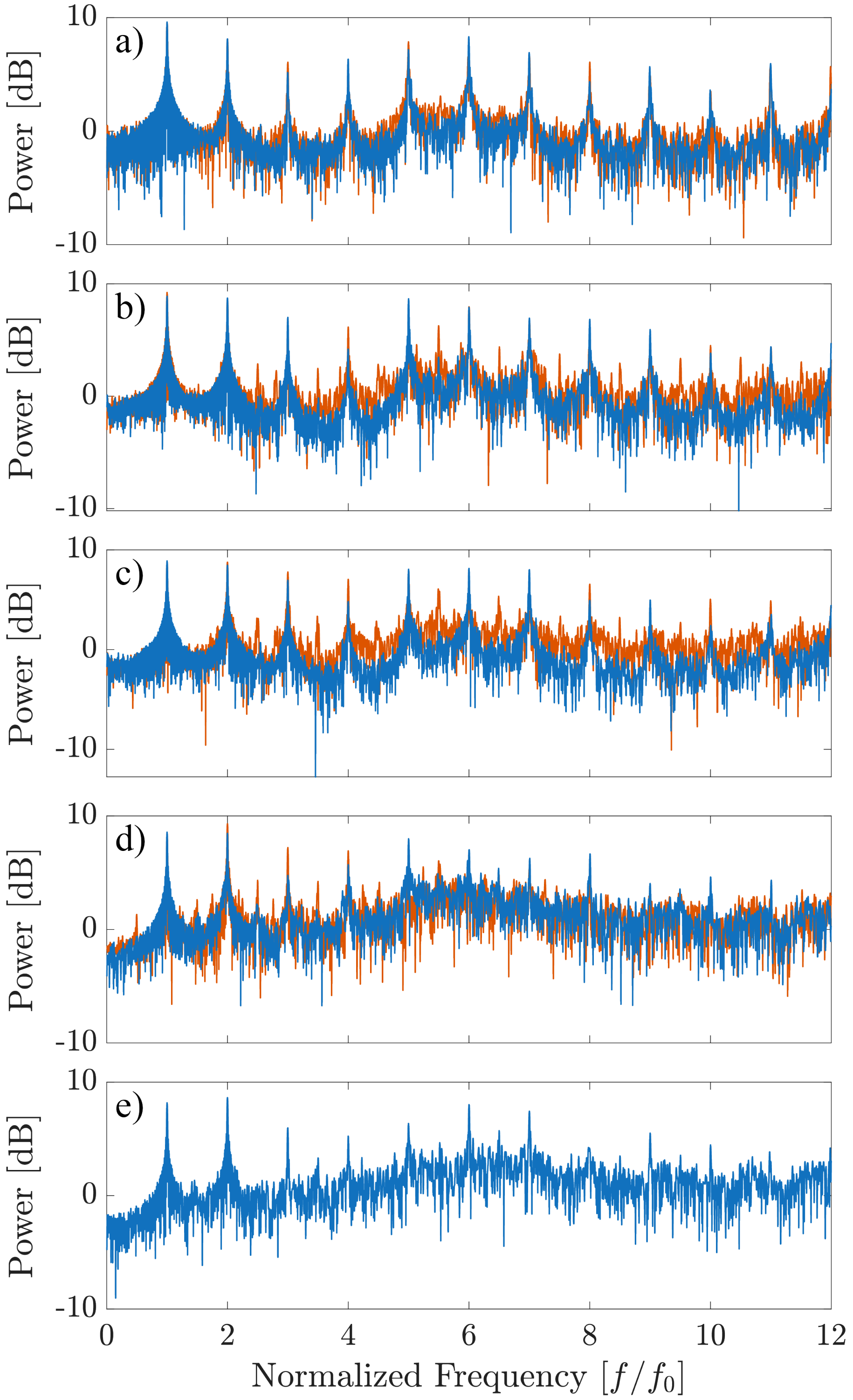

Figure 1: Experimental spectra showing the blue curves for the increasing series and the red curves for the decreasing series, under excitation amplitudes of (a) 180 mVpp, (b) 225 mVpp, (c) 270 mVpp, (d) 315 mVpp, and (e) 360 mVpp.

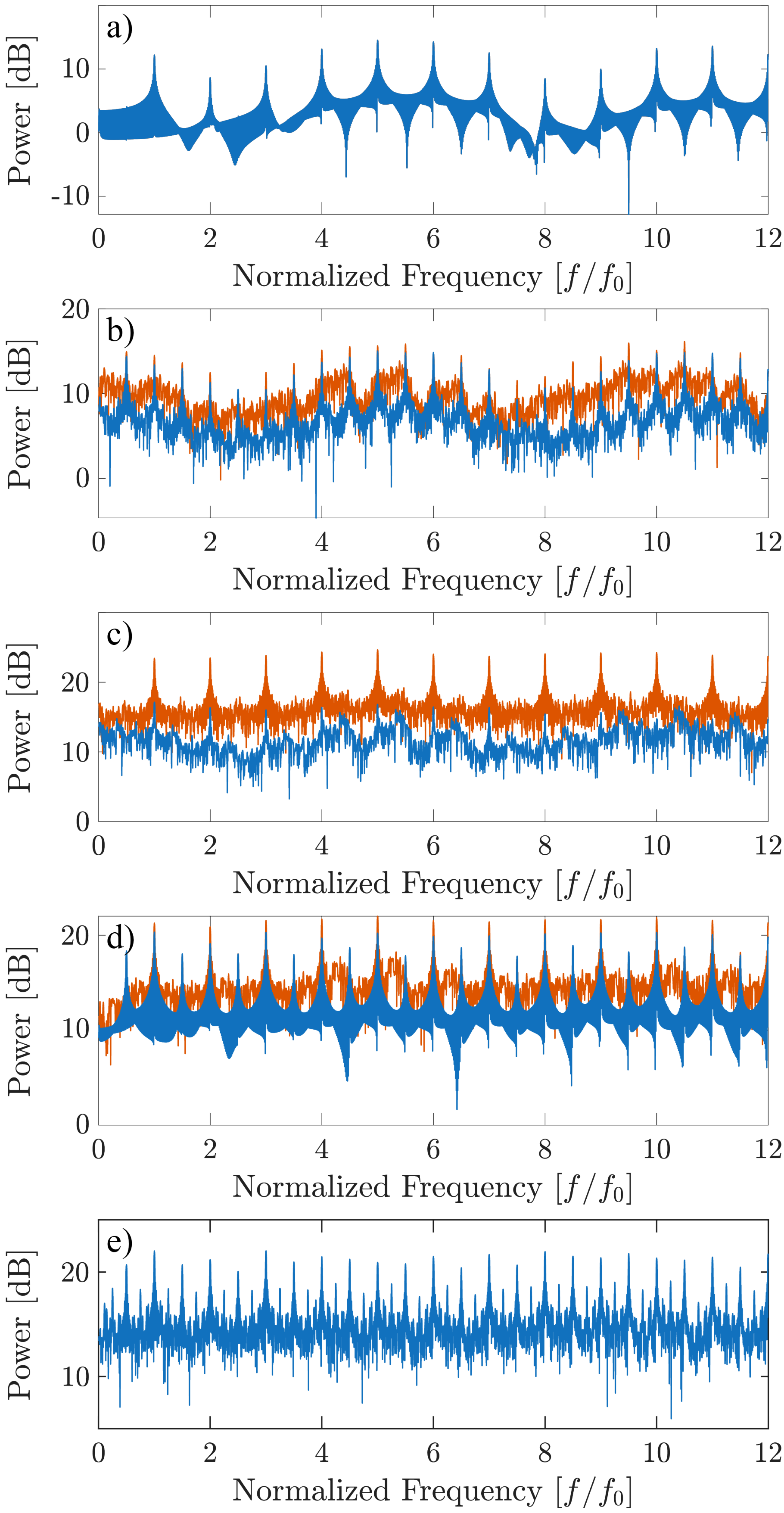

Figure 2: Numerical spectra showing the blue curves for the increasing series and the red curves for the decreasing series, under excitation amplitudes of (a) 75 kPa, (b) 79.5 kPa, (c) 84 kPa, (d) 88.5 kPa, and (e) 93 kPa.

References

[1] Jacques Frohly, Stephane Labouret, Claude Bruneel, Isabelle Looten-Baquet, and Roland Torguet. Ultrasonic cavitation monitoring by acoustic noise power measurement. *The Journal of the Acoustical Society of America*, 108(5):2012–2020, 2000.

[2] Pauline Muleki Seya, Cyril Desjouy, Jean-Christophe Béra, and Claude Inserra. Hysteresis of inertial cavitation activity induced by fluctuating bubble size distribution. *Ultrasonics Sonochemistry*, 27:262–267, 2015.
