# Supplementary material for: Hysteresis in cavitation emissions during a ramped-then-deramped amplitude sonication: A theoretical and experimental investigation
Source: Nonlinear Dyn. 2026 Apr 21;114(8):591. doi: 10.1007/s11071-026-12462-3 (PMC13100018; doi:10.1007/s11071-026-12462-3)
Supplement: Supplementary file 2 — (pdf 415 KB) [file 11071_2026_12462_MOESM2_ESM.pdf]

Hysteresis in cavitation emissions during a ramped-then-deramped amplitude sonication
A theoretical and experimental investigation
Supplementary Material 2: Bubble Distribution

Y. Zhang[1], S. Li[1], P. Prentice[1] and A. Cammarano[2]
[1]Cavitation Laboratory, Centre for Medical and Industrial Ultrasonics,
University of Glasgow, University Avenue, Glasgow, G12 8QQ, UK
[2]Department of Aeronautics and Astronautics,
University of Southampton, Burgess Road, Southampton, SO16 7QF, UK
email: andrea.cammarano@soton.ac.uk

Journal: Nonlinear Dynamics

The acoustic emissions of multi-bubble systems with two different spatial distributions were investigated using spectrogram analysis. The results demonstrate the generality of hysteresis across different bubble arrangements. In the first configuration (Fig. S2.1), eleven bubbles are evenly distributed along a circle, and the corresponding spectrogram is shown in Fig. S2.2. Clear hysteresis is shown, with a stronger broadband noise appearing during an interval in the ramp phase. However, because all eleven bubbles are equivalent, unlike the distribution in real experiments, the resulting spectrogram differs from Fig. 2(b) in the main manuscript: the re-emergence of broadband noise and the brief interval of increased broadband during the deramp phase are absent. When a central bubble (bubble 1) is introduced at the center of the circle, as shown in Fig. S2.3, the equivalence among bubbles is broken. In this configuration, the spectrogram, Fig. S2.4, more closely resembles Fig. 2(b), showing both the re-emergence of broadband noise and the short interval of increased broadband during the deramp.

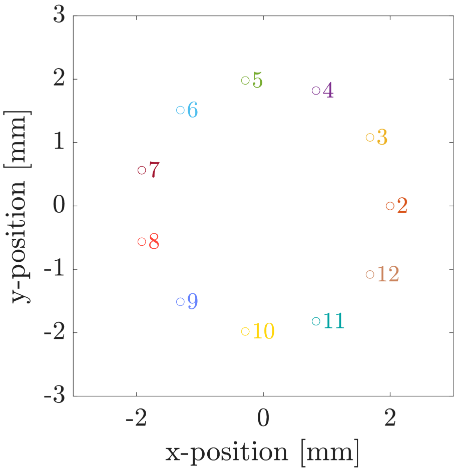

Figure S2.1: The distribution of a 11-bubble system along a circle.

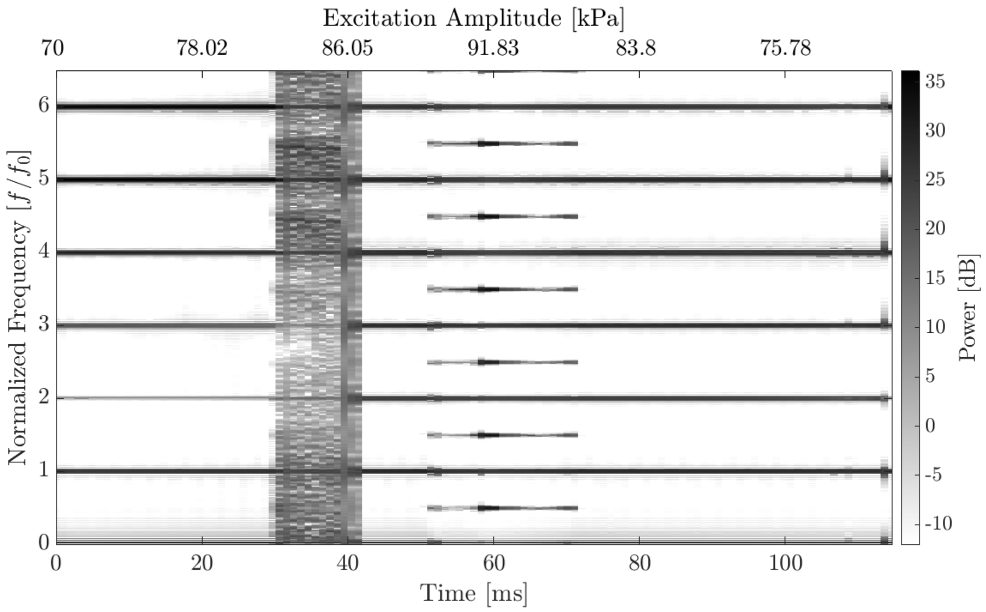

Figure S2.2: The spectrogram result of the distribution shown in Fig. S2.1.

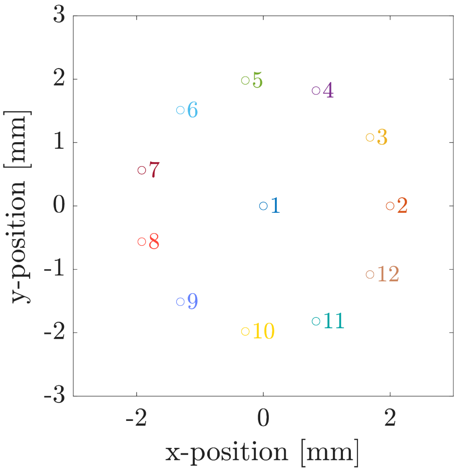

Figure S2.3: The distribution of a 12-bubble system.

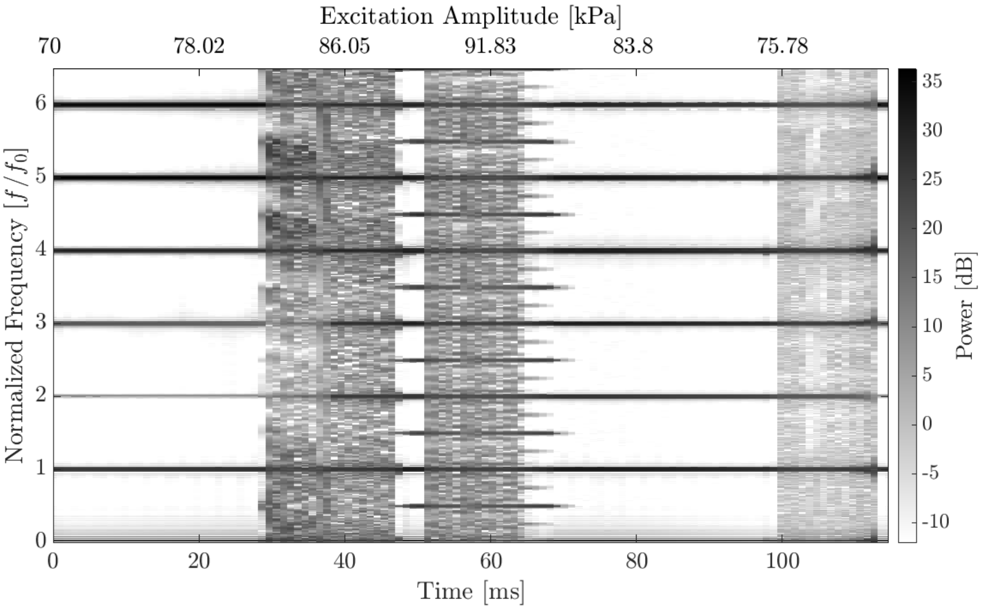

Figure S2.4: The spectrogram result of the distribution shown in Fig. S2.3.
