# Supplementary material for: Hysteresis in cavitation emissions during a ramped-then-deramped amplitude sonication: A theoretical and experimental investigation
Source: Nonlinear Dyn. 2026 Apr 21;114(8):591. doi: 10.1007/s11071-026-12462-3 (PMC13100018; doi:10.1007/s11071-026-12462-3)
Supplement: Supplementary file 3 — (pdf 779 KB) [file 11071_2026_12462_MOESM3_ESM.pdf]

Hysteresis in cavitation emissions during a ramped-then-deramped amplitude sonication

A theoretical and experimental investigation

Supplementary Material 3: Steady-state Analysis

Y. Zhang[1], S. Li[1], P. Prentice[1] and A. Cammarano[2]

[1]Cavitation Laboratory, Centre for Medical and Industrial Ultrasonics,
University of Glasgow, University Avenue, Glasgow, G12 8QQ, UK

[2]Department of Aeronautics and Astronautics,
University of Southampton, Burgess Road, Southampton, SO16 7QF, UK

email: andrea.cammarano@soton.ac.uk

Journal: Nonlinear Dynamics

The bifurcation diagram, Fig. S3.1, is the result of a single bubble from the 12-bubble system. Stable and unstable attractors are identified, with saddle-node and period-doubling bifurcation points marked by white and red diamonds, respectively. This bifurcation diagram represents the 12-bubble system under small interactions. To show the evolution of the bifurcation structure with increasing interactions, i.e., decreasing scaling factors, Fig. S3.2 is presented.

Panel (a) and (b) of Fig. S3.2, together with Fig. 7(b) in the main manuscript, show the bifurcation diagrams under three different scaling factors: 100, 2, and 1 respectively. With a scaling factor of 100, the interaction between bubbles is negligible – this results in the consistent result of the stable branches shown in Fig. S3.2(a) with the bifurcation diagram, Fig. S3.1. The evolution of scaling factors connects Fig. S3.1 to Fig. 7(b) with the qualitative consistency in the bifurcation structure. This analysis also confirms the coexistence of multiple attractors at identical excitation amplitudes under different scaling factors.

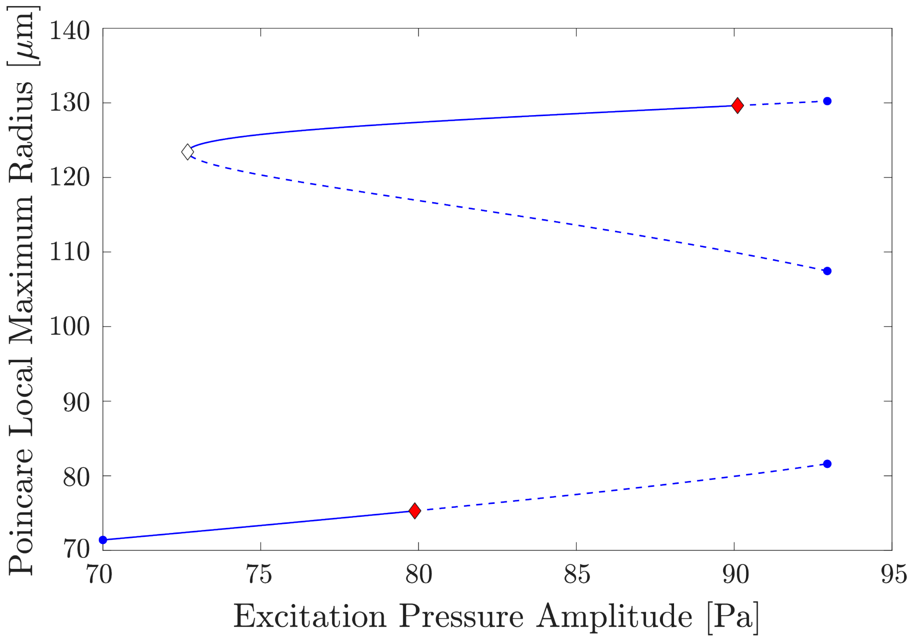

Figure S3.1: Bifurcation diagram obtained via boundary value problem using the COCO continuation software. The solid and dashed curves are the stable and unstable solutions, respectively. Saddle-node and period-doubling bifurcations are marked by white and red diamonds, respectively.

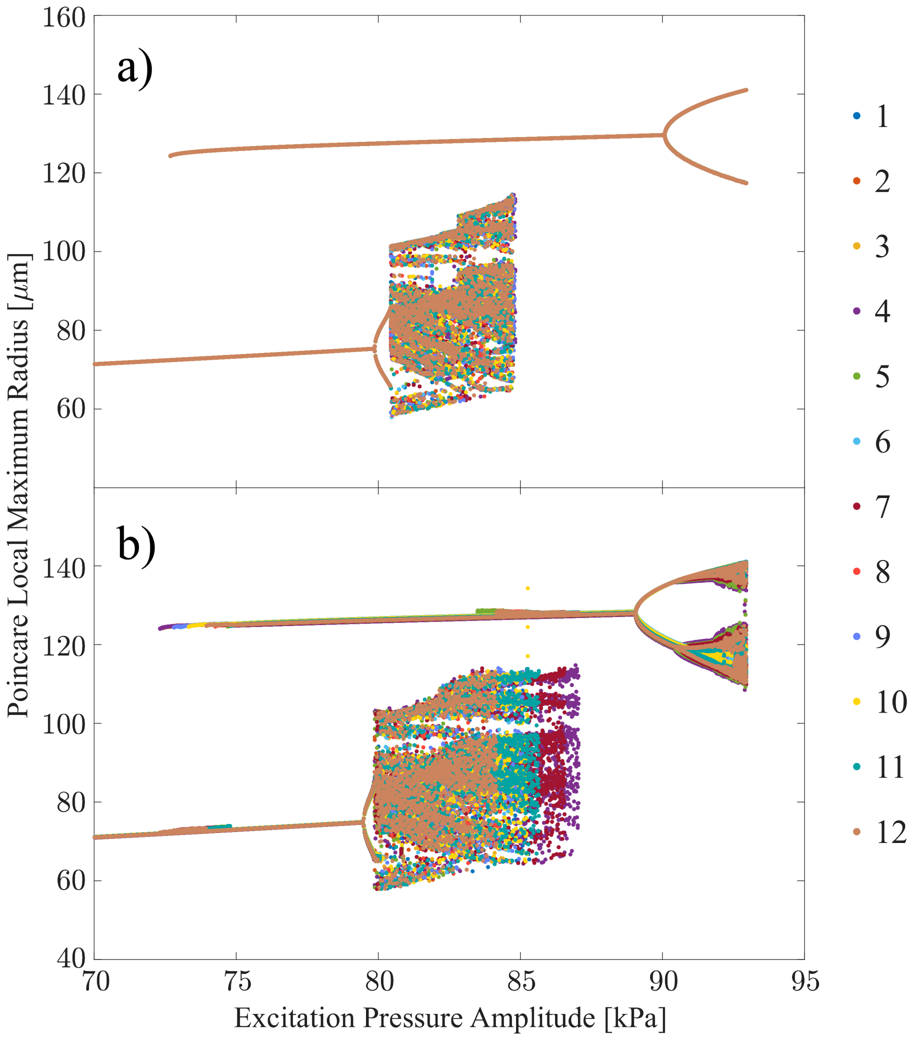

Figure S3.2: Bifurcation diagram generated from increased-then-decreased series of 1758 excitation amplitudes under the scaling factor of (a) 100 (b) 2.
