# Supplementary material for: Hysteresis in cavitation emissions during a ramped-then-deramped amplitude sonication: A theoretical and experimental investigation
Source: Nonlinear Dyn. 2026 Apr 21;114(8):591. doi: 10.1007/s11071-026-12462-3 (PMC13100018; doi:10.1007/s11071-026-12462-3)
Supplement: Supplementary file 4 — (pdf 10364 KB) [file 11071_2026_12462_MOESM4_ESM.pdf]

Hysteresis in cavitation emissions during a ramped-then-deramped amplitude sonication
A theoretical and experimental investigation
Supplementary Material 4: Control bubble

Y. Zhang[1], S. Li[1], P. Prentice[1] and A. Cammarano[2]
[1]Cavitation Laboratory, Centre for Medical and Industrial Ultrasonics,
University of Glasgow, University Avenue, Glasgow, G12 8QQ, UK
[2]Department of Aeronautics and Astronautics,
University of Southampton, Burgess Road, Southampton, SO16 7QF, UK
email: andrea.cammarano@soton.ac.uk

Journal: Nonlinear Dynamics

The basin-of-attraction analysis is extended beyond the examples shown in the main text (see Fig. 9 and Fig. 10) by including (i) the basins of attraction for all bubbles when bubble 2 serves as the control bubble, presented as twelve figures in the subsections below; and (ii) the cases where each bubble in turn is designated as the control bubble, with results for all twelve cases presented in twelve corresponding subsections. The results confirm that, even under identical initial conditions for the control bubble, different bubbles within the system can converge to distinct attractors in both amplitude and periodicity. This diversity of responses highlights the influence of bubble-bubble coupling arising from spatial distribution.

Control bubble: Bubble 1

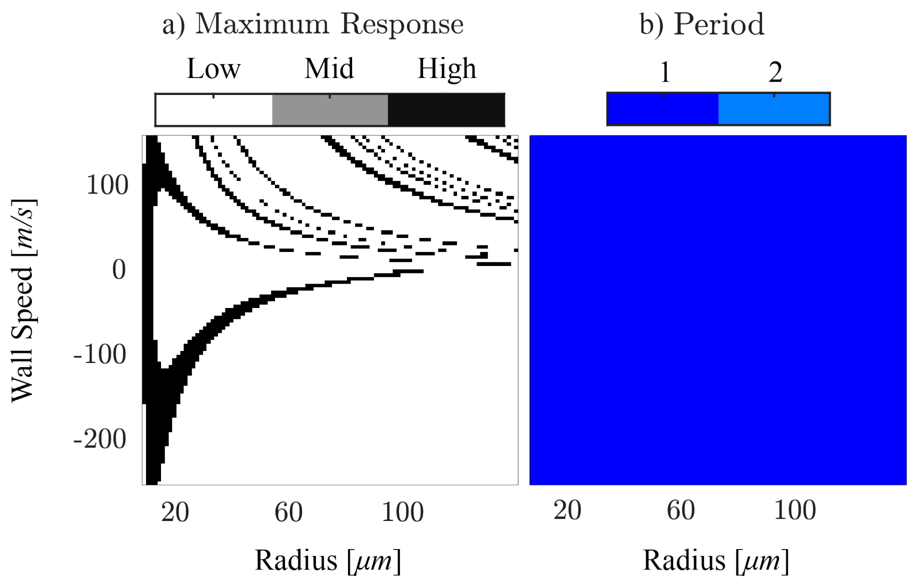

Figure S4.1: (a) the amplitude basins for bubble 1, where low-, mid- and high-amplitude attractor correspond to white, gray and black; and (b) the corresponding period basins of these attractors, as indicated by the colorbar.

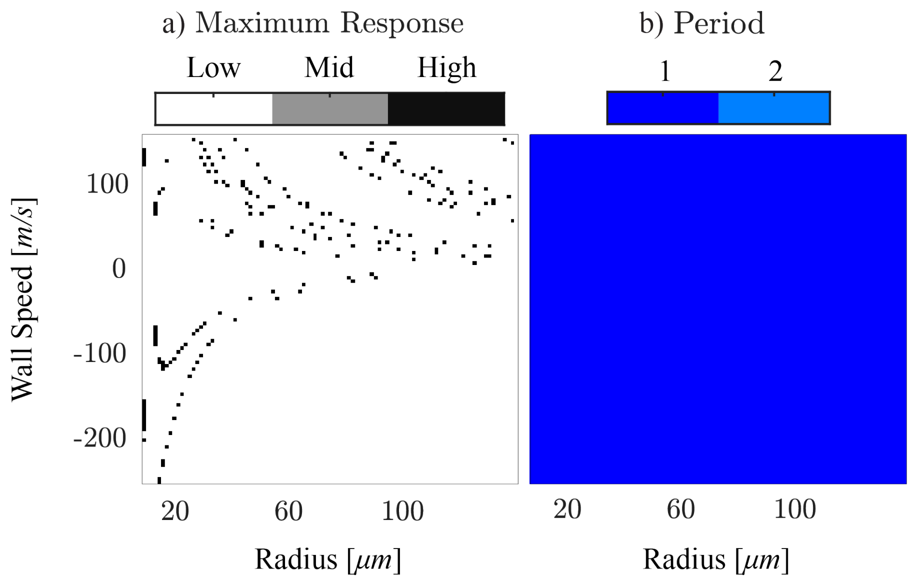

Figure S4.2: (a) the amplitude basins for bubble 2, where low-, mid- and high-amplitude attractor correspond to white, gray and black; and (b) the corresponding period basins of these attractors, as indicated by the colorbar.

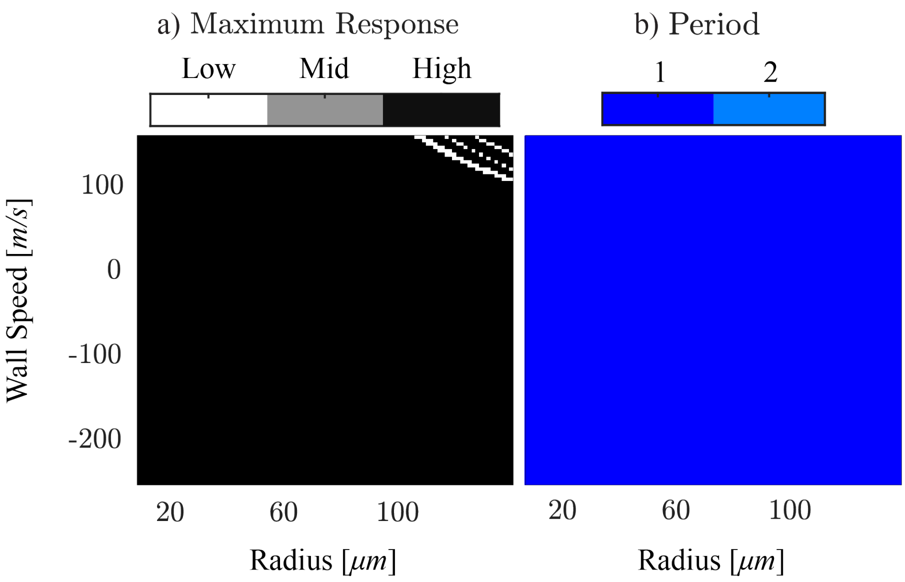

Figure S4.3: (a) the amplitude basins for bubble 3, where low-, mid- and high-amplitude attractor correspond to white, gray and black; and (b) the corresponding period basins of these attractors, as indicated by the colorbar.

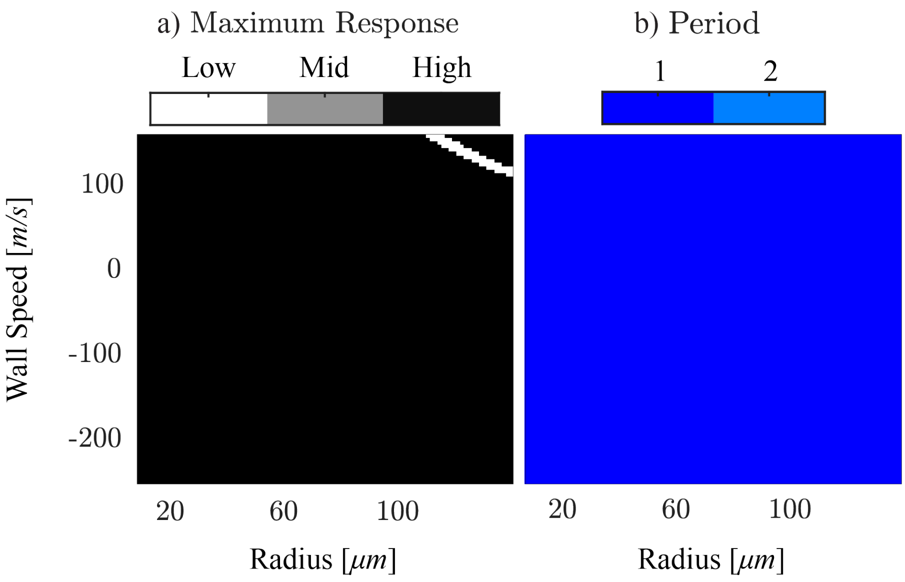

Figure S4.4: (a) the amplitude basins for bubble 4, where low-, mid- and high-amplitude attractor correspond to white, gray and black; and (b) the corresponding period basins of these attractors, as indicated by the colorbar.

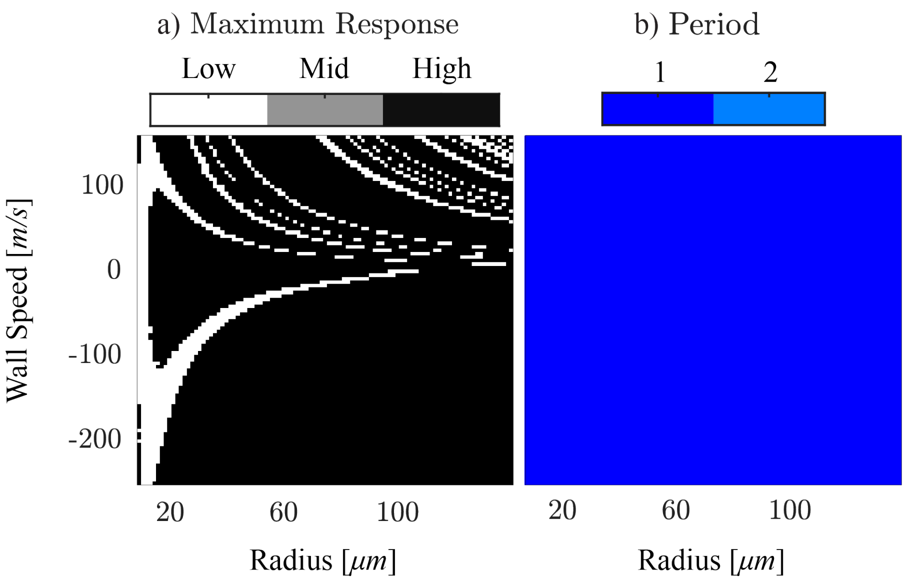

Figure S4.5: (a) the amplitude basins for bubble 5, where low-, mid- and high-amplitude attractor correspond to white, gray and black; and (b) the corresponding period basins of these attractors, as indicated by the colorbar.

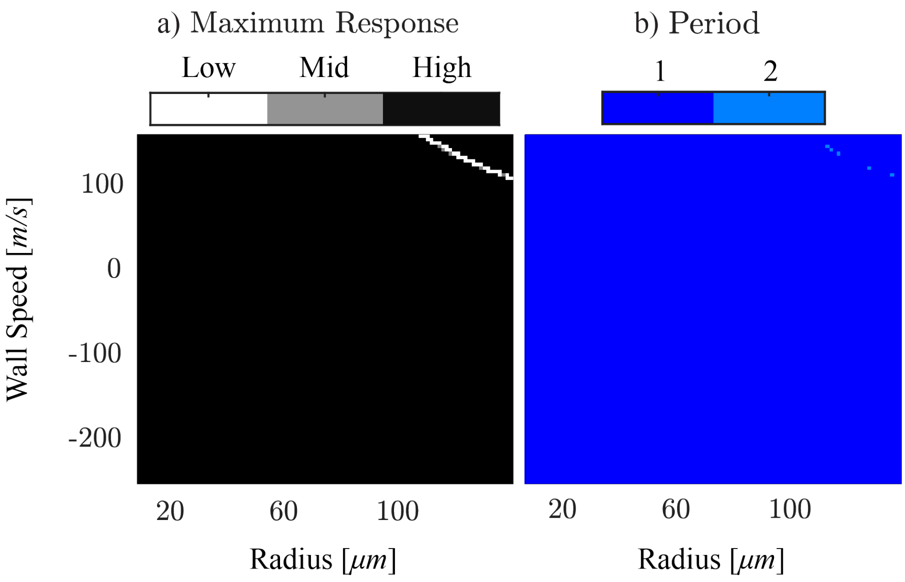

Figure S4.6: (a) the amplitude basins for bubble 6, where low-, mid- and high-amplitude attractor correspond to white, gray and black; and (b) the corresponding period basins of these attractors, as indicated by the colorbar.

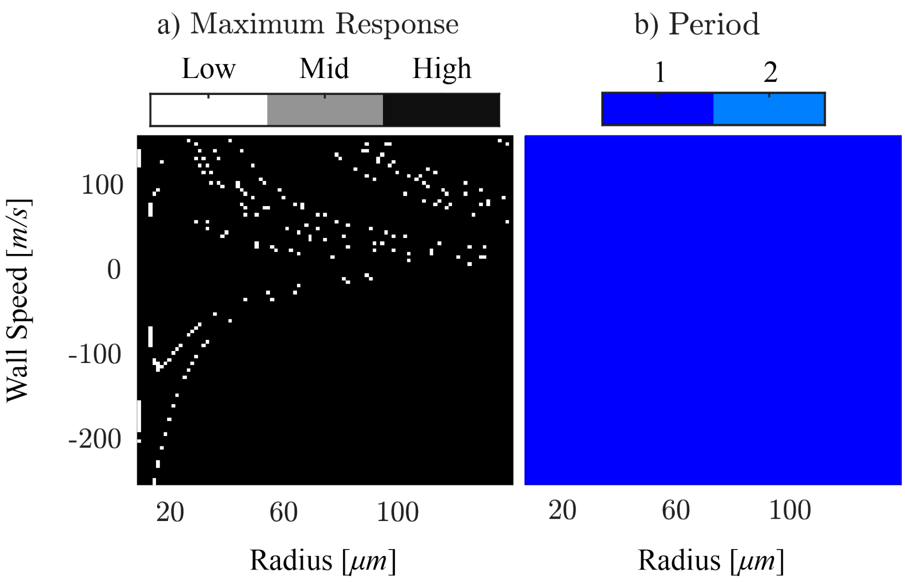

Figure S4.7: (a) the amplitude basins for bubble 7, where low-, mid- and high-amplitude attractor correspond to white, gray and black; and (b) the corresponding period basins of these attractors, as indicated by the colorbar.

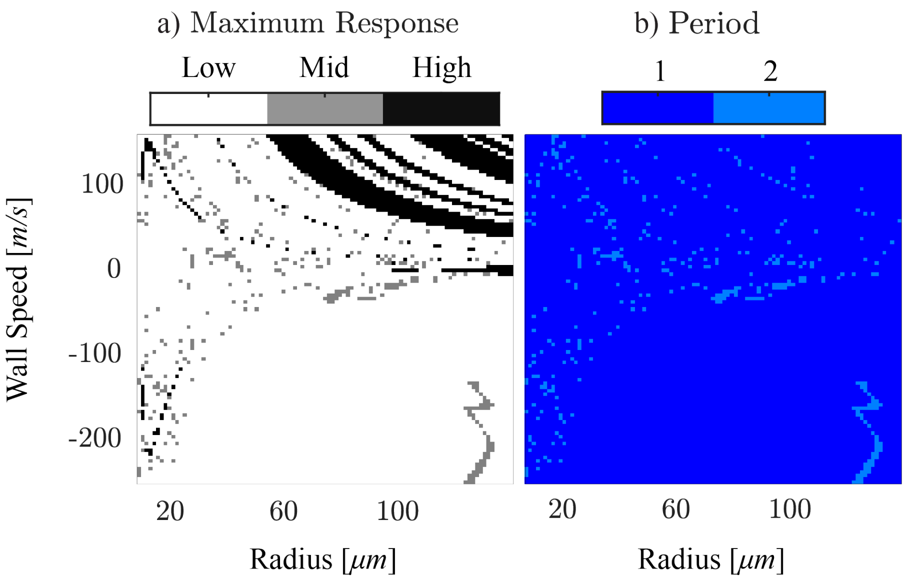

Figure S4.8: (a) the amplitude basins for bubble 8, where low-, mid- and high-amplitude attractor correspond to white, gray and black; and (b) the corresponding period basins of these attractors, as indicated by the colorbar.

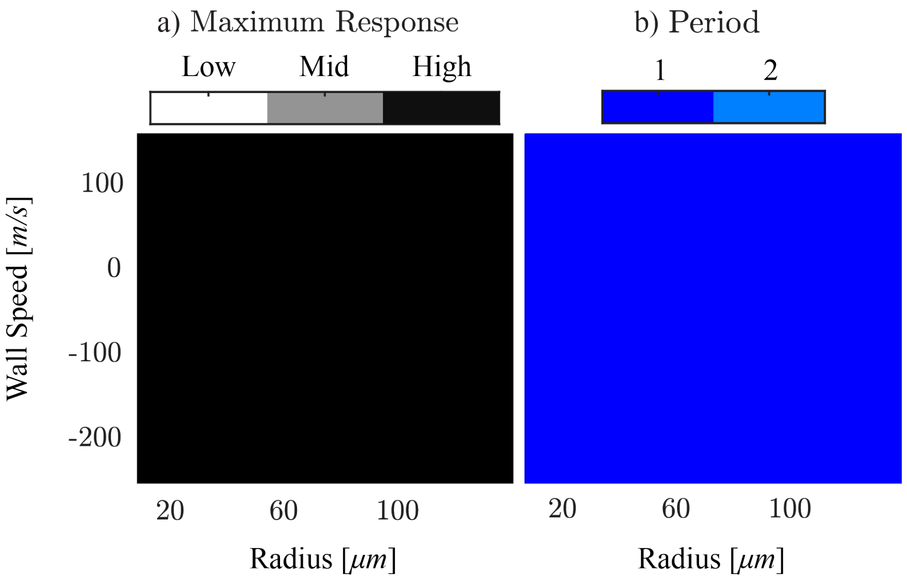

Figure S4.9: (a) the amplitude basins for bubble 9, where low-, mid- and high-amplitude attractor correspond to white, gray and black; and (b) the corresponding period basins of these attractors, as indicated by the colorbar.

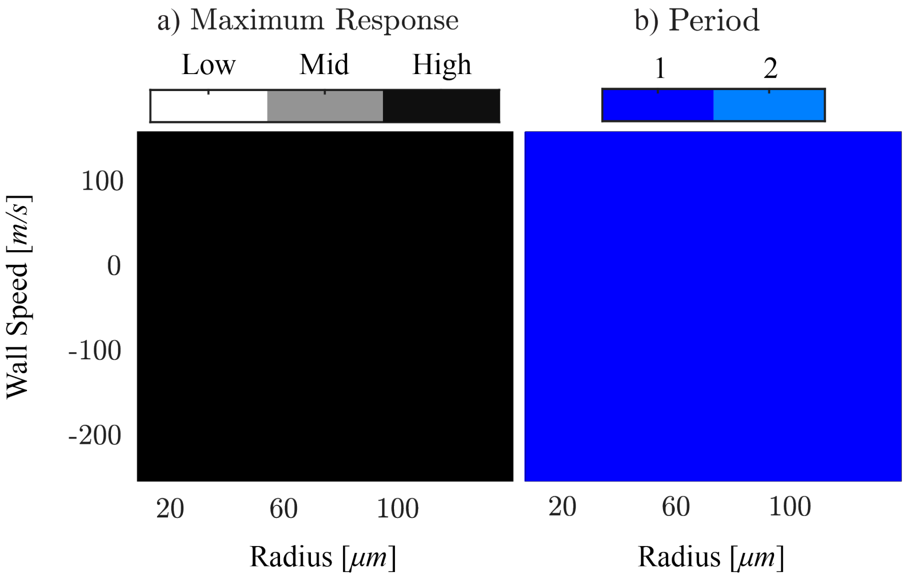

Figure S4.10: (a) the amplitude basins for bubble 10, where low-, mid- and high-amplitude attractor correspond to white, gray and black; and (b) the corresponding period basins of these attractors, as indicated by the colorbar.

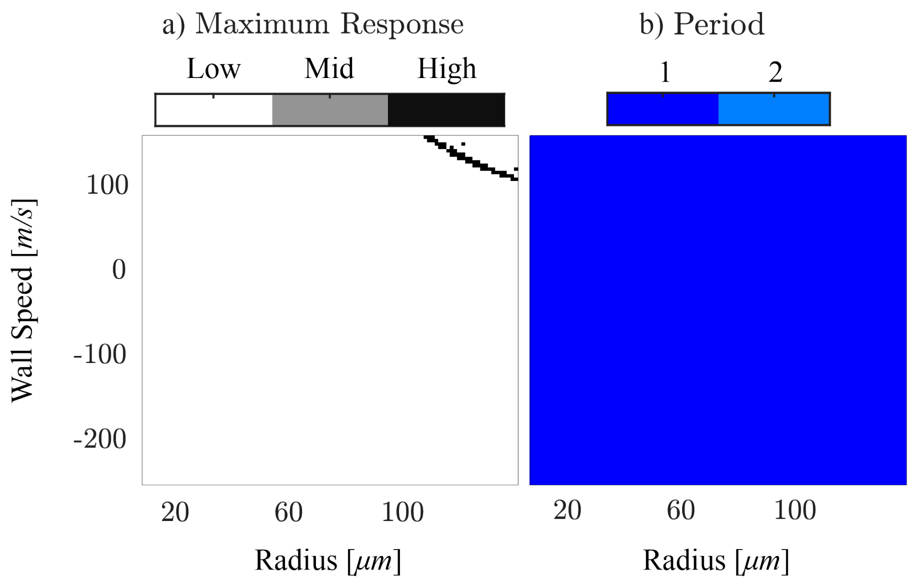

Figure S4.11: (a) the amplitude basins for bubble 11, where low-, mid- and high-amplitude attractor correspond to white, gray and black; and (b) the corresponding period basins of these attractors, as indicated by the colorbar.

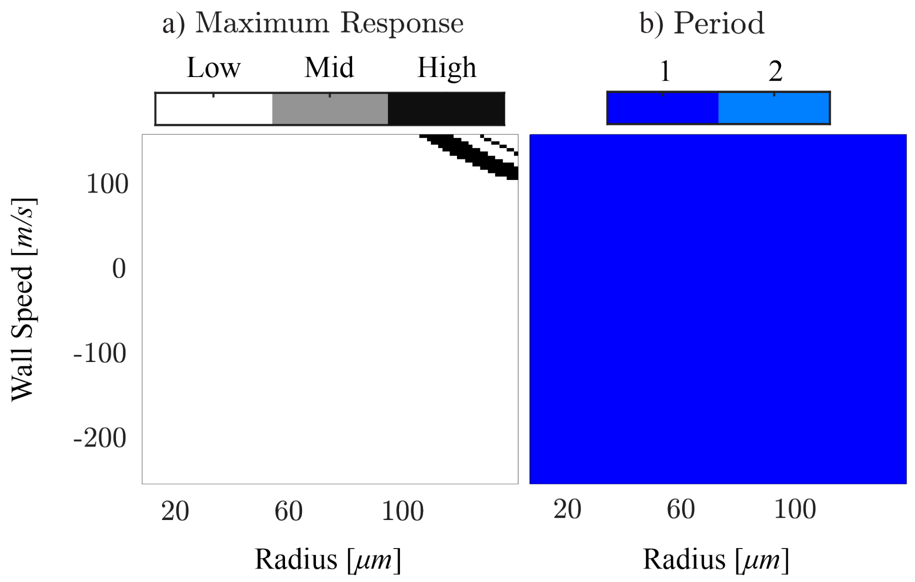

Figure S4.12: (a) the amplitude basins for bubble 12, where low-, mid- and high-amplitude attractor correspond to white, gray and black; and (b) the corresponding period basins of these attractors, as indicated by the colorbar.

Control bubble: Bubble 2

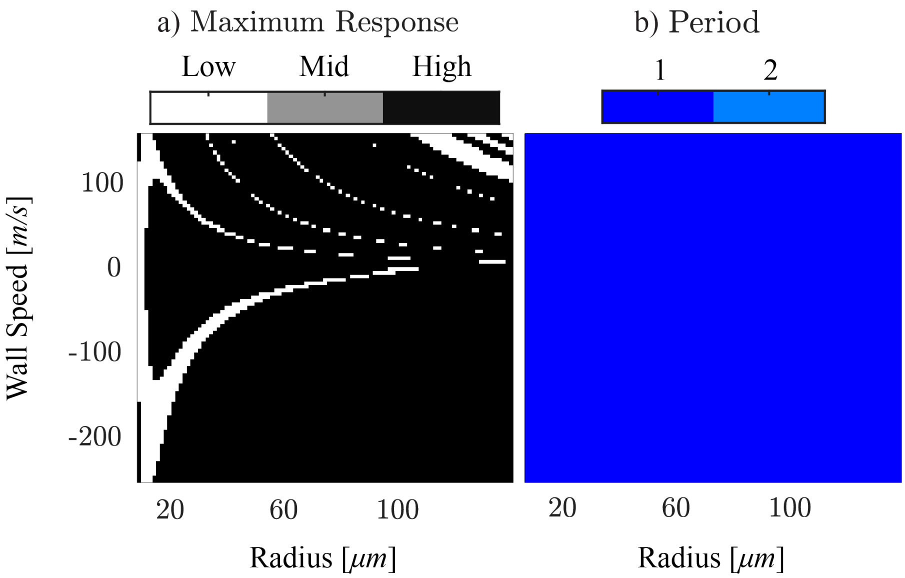

Figure S4.13: (a) the amplitude basins for bubble 1, where low-, mid- and high-amplitude attractor correspond to white, gray and black; and (b) the corresponding period basins of these attractors, as indicated by the colorbar.

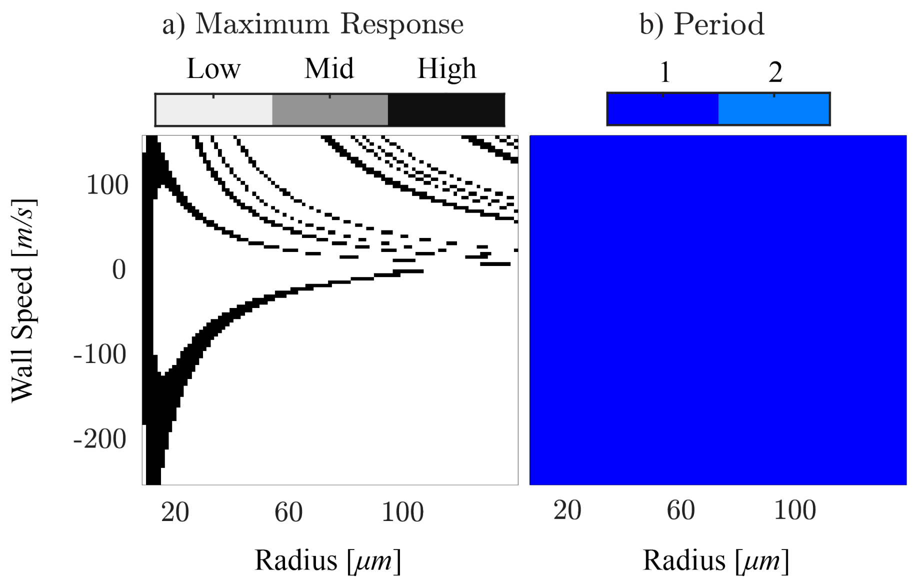

Figure S4.14: (a) the amplitude basins for bubble 2, where low-, mid- and high-amplitude attractor correspond to white, gray and black; and (b) the corresponding period basins of these attractors, as indicated by the colorbar.

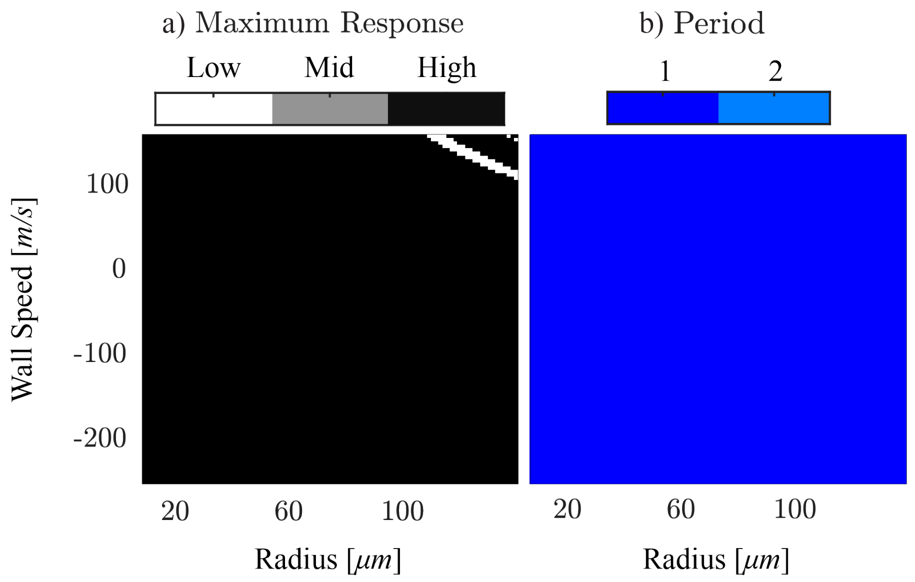

Figure S4.15: (a) the amplitude basins for bubble 3, where low-, mid- and high-amplitude attractor correspond to white, gray and black; and (b) the corresponding period basins of these attractors, as indicated by the colorbar.

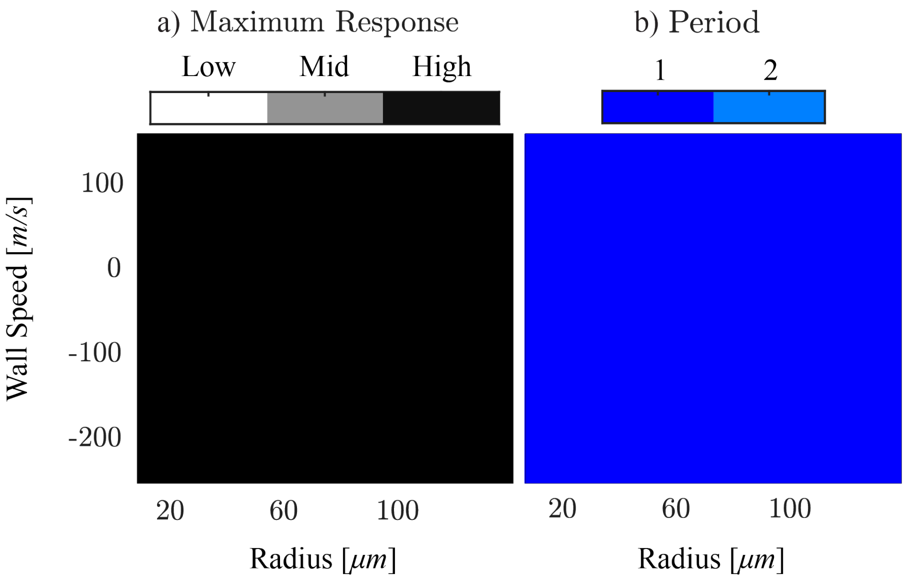

Figure S4.16: (a) the amplitude basins for bubble 4, where low-, mid- and high-amplitude attractor correspond to white, gray and black; and (b) the corresponding period basins of these attractors, as indicated by the colorbar.

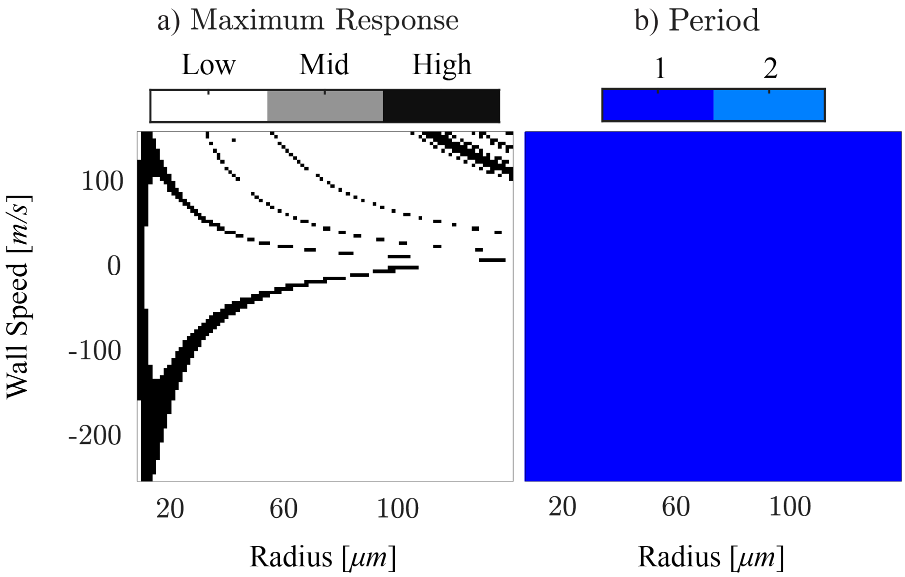

Figure S4.17: (a) the amplitude basins for bubble 5, where low-, mid- and high-amplitude attractor correspond to white, gray and black; and (b) the corresponding period basins of these attractors, as indicated by the colorbar.

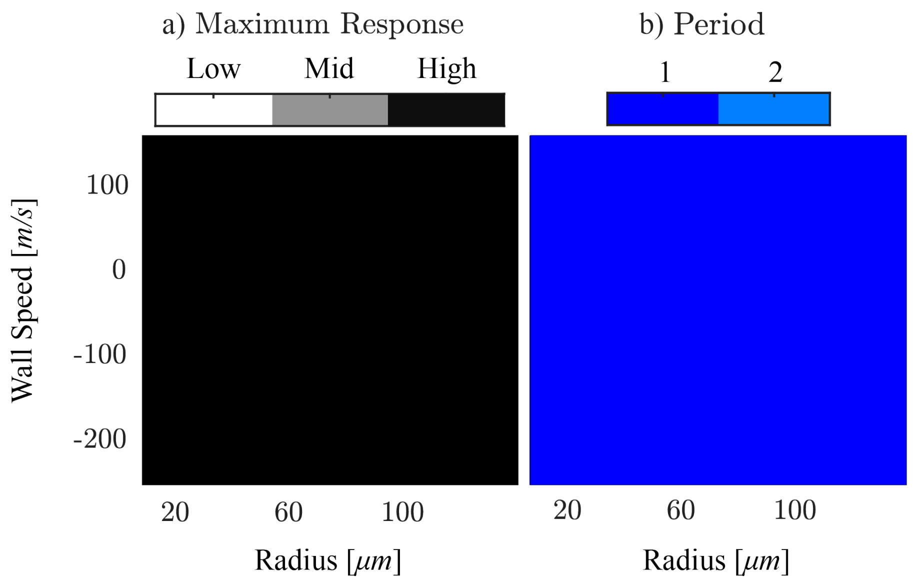

Figure S4.18: (a) the amplitude basins for bubble 6, where low-, mid- and high-amplitude attractor correspond to white, gray and black; and (b) the corresponding period basins of these attractors, as indicated by the colorbar.

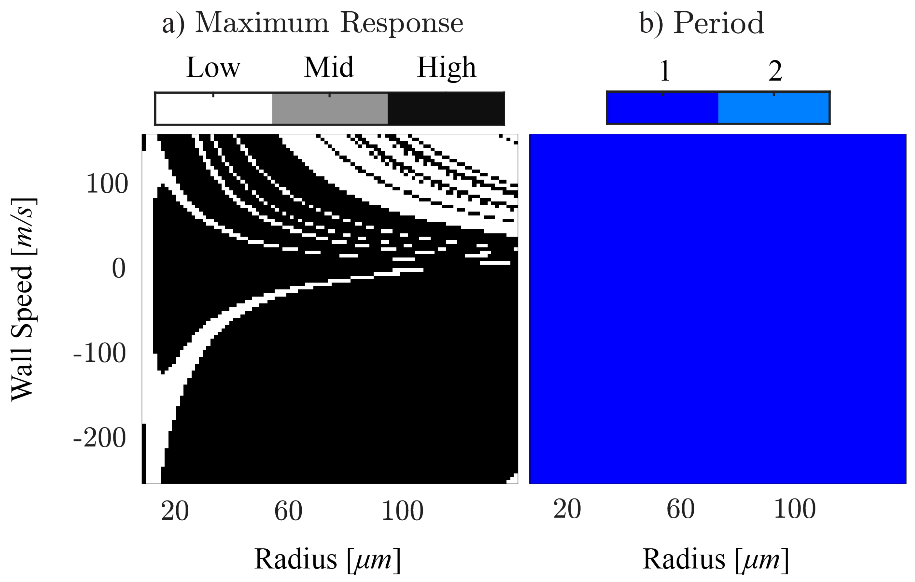

Figure S4.19: (a) the amplitude basins for bubble 7, where low-, mid- and high-amplitude attractor correspond to white, gray and black; and (b) the corresponding period basins of these attractors, as indicated by the colorbar.

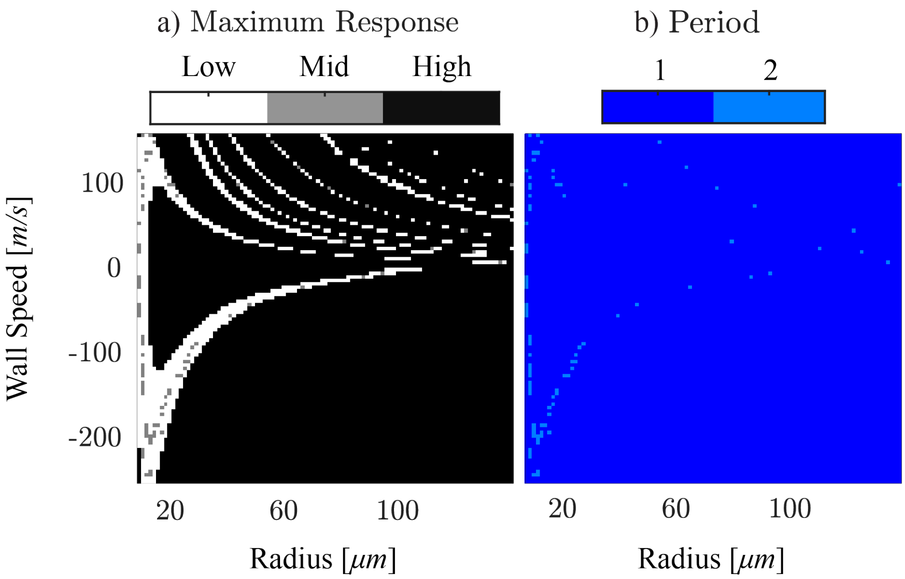

Figure S4.20: (a) the amplitude basins for bubble 8, where low-, mid- and high-amplitude attractor correspond to white, gray and black; and (b) the corresponding period basins of these attractors, as indicated by the colorbar.

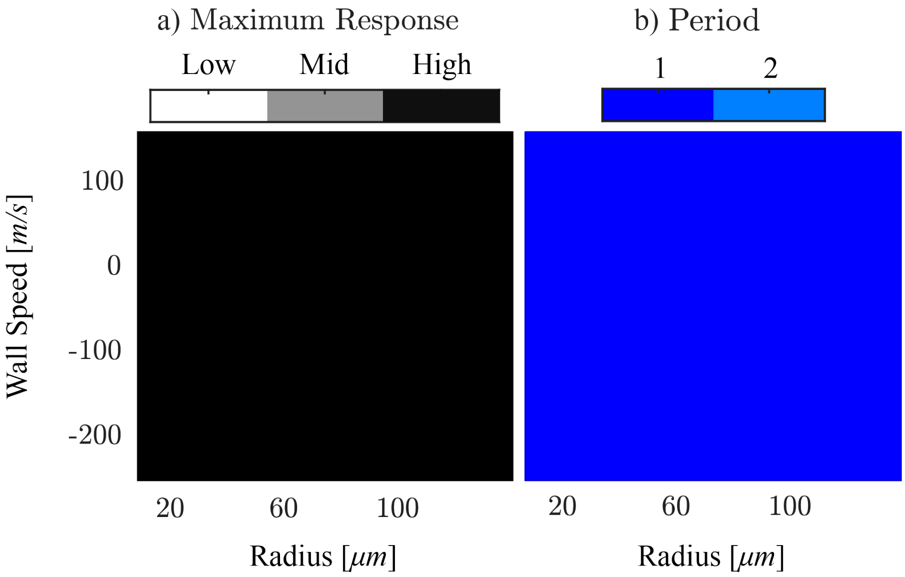

Figure S4.21: (a) the amplitude basins for bubble 9, where low-, mid- and high-amplitude attractor correspond to white, gray and black; and (b) the corresponding period basins of these attractors, as indicated by the colorbar.

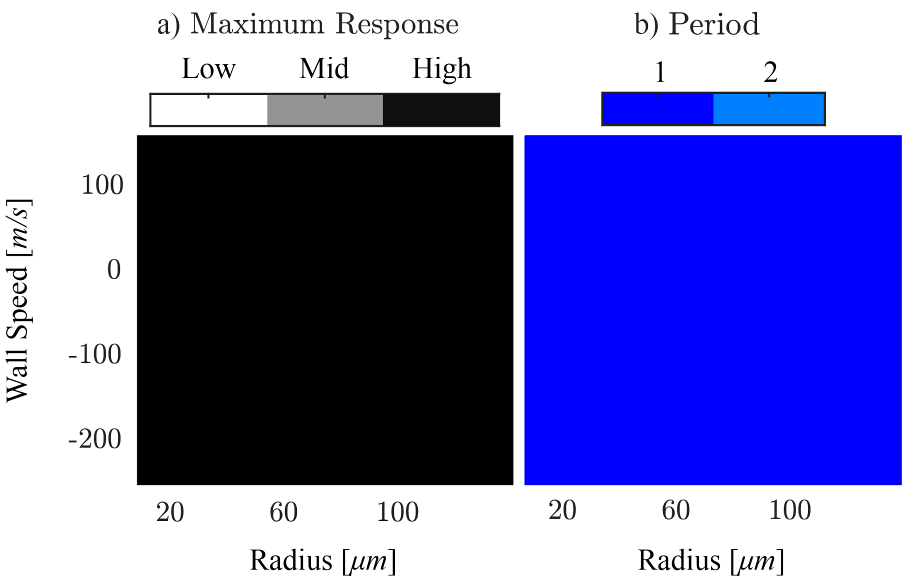

Figure S4.22: (a) the amplitude basins for bubble 10, where low-, mid- and high-amplitude attractor correspond to white, gray and black; and (b) the corresponding period basins of these attractors, as indicated by the colorbar.

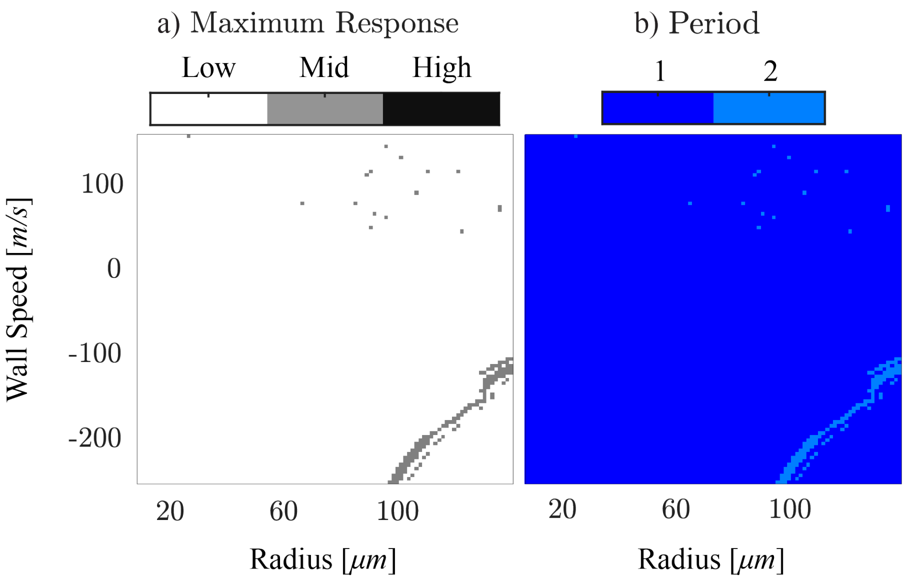

Figure S4.23: (a) the amplitude basins for bubble 11, where low-, mid- and high-amplitude attractor correspond to white, gray and black; and (b) the corresponding period basins of these attractors, as indicated by the colorbar.

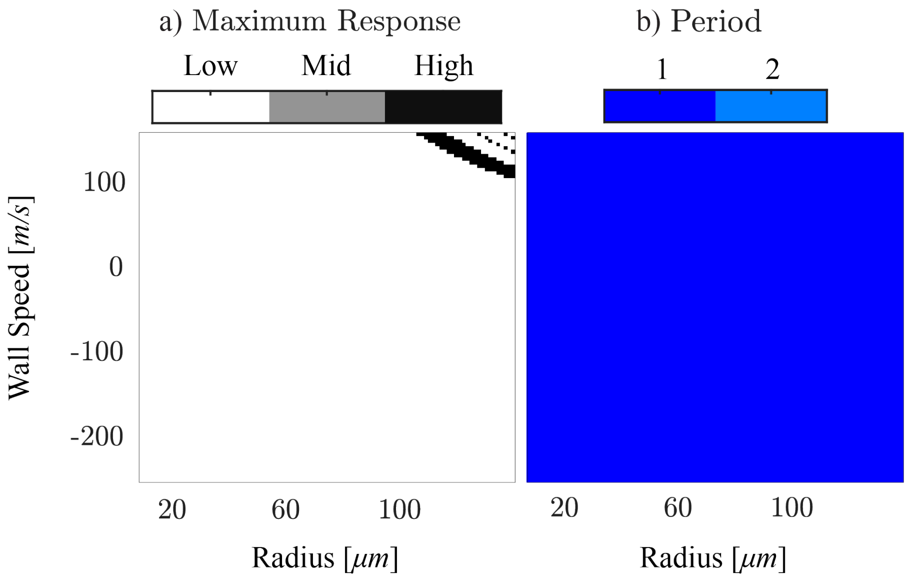

Figure S4.24: (a) the amplitude basins for bubble 12, where low-, mid- and high-amplitude attractor correspond to white, gray and black; and (b) the corresponding period basins of these attractors, as indicated by the colorbar.

Control bubble: Bubble 3

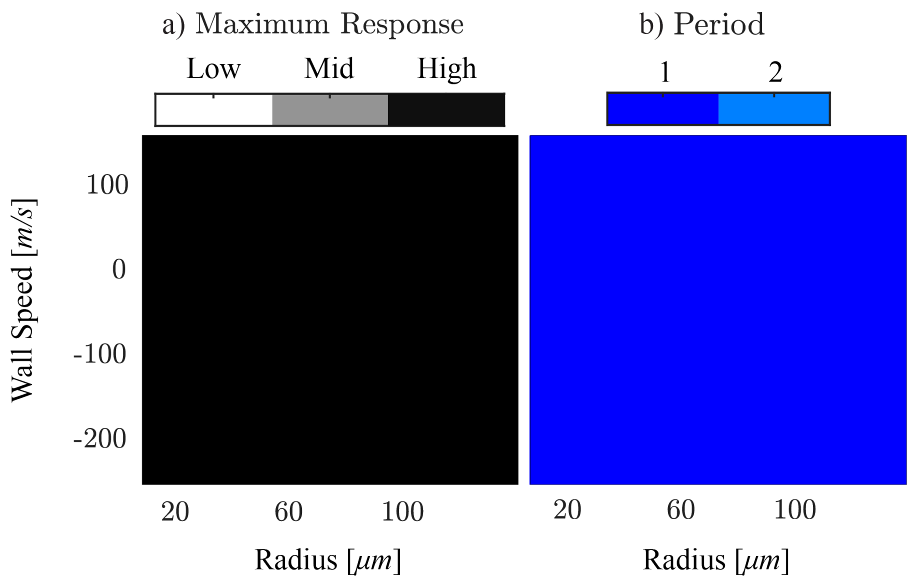

Figure S4.25: (a) the amplitude basins for bubble 1, where low-, mid- and high-amplitude attractor correspond to white, gray and black; and (b) the corresponding period basins of these attractors, as indicated by the colorbar.

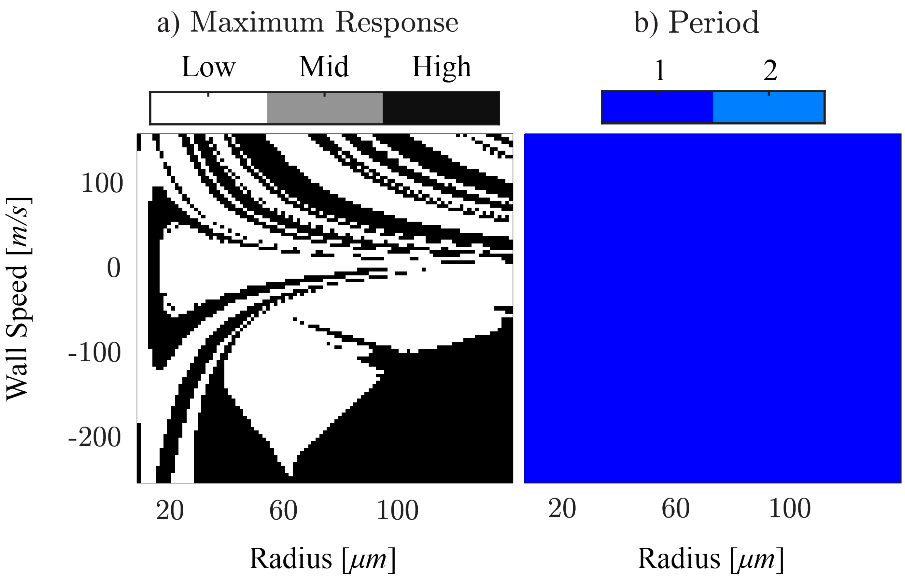

Figure S4.26: (a) the amplitude basins for bubble 2, where low-, mid- and high-amplitude attractor correspond to white, gray and black; and (b) the corresponding period basins of these attractors, as indicated by the colorbar.

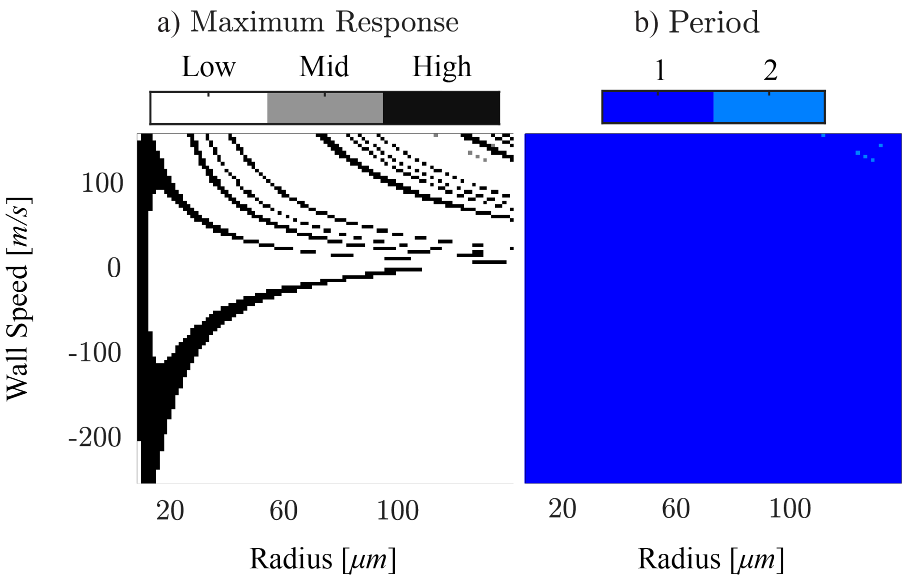

Figure S4.27: (a) the amplitude basins for bubble 3, where low-, mid- and high-amplitude attractor correspond to white, gray and black; and (b) the corresponding period basins of these attractors, as indicated by the colorbar.

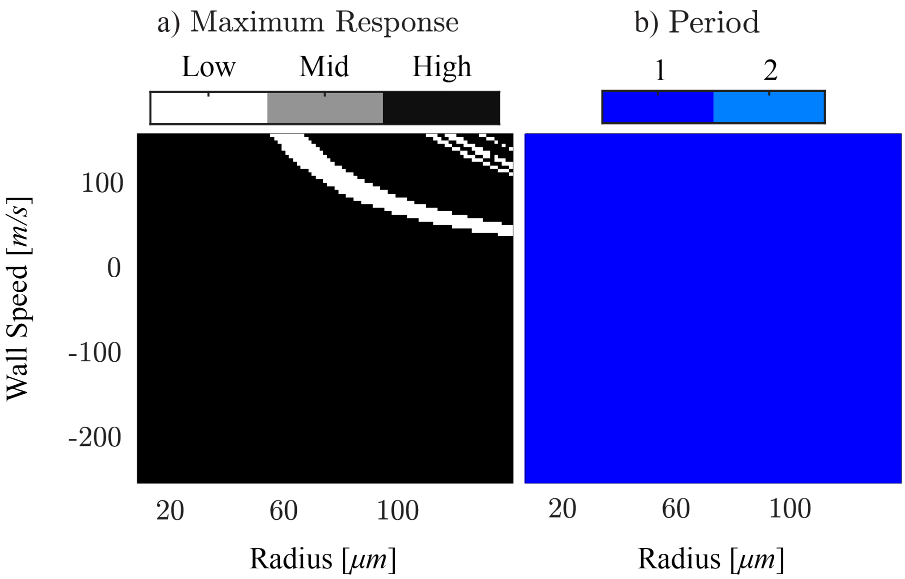

Figure S4.28: (a) the amplitude basins for bubble 4, where low-, mid- and high-amplitude attractor correspond to white, gray and black; and (b) the corresponding period basins of these attractors, as indicated by the colorbar.

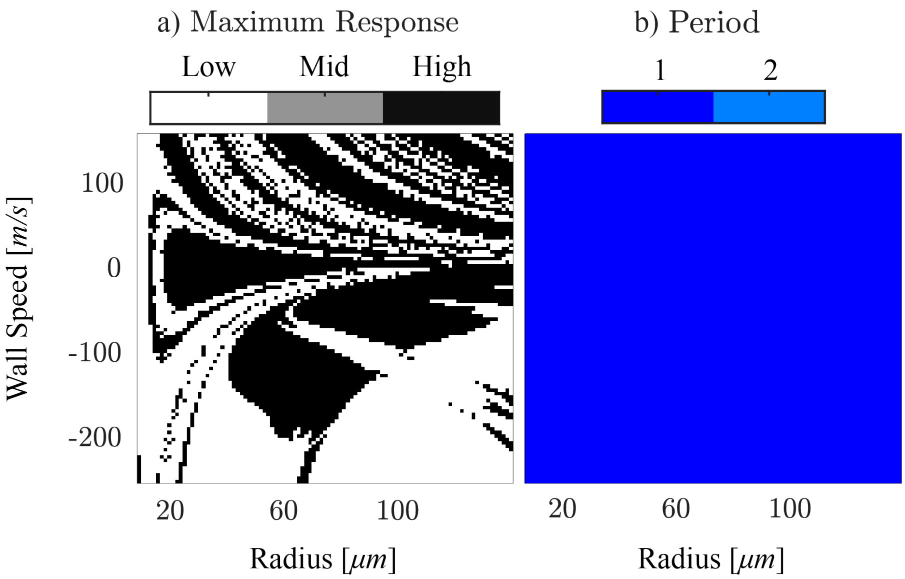

Figure S4.29: (a) the amplitude basins for bubble 5, where low-, mid- and high-amplitude attractor correspond to white, gray and black; and (b) the corresponding period basins of these attractors, as indicated by the colorbar.

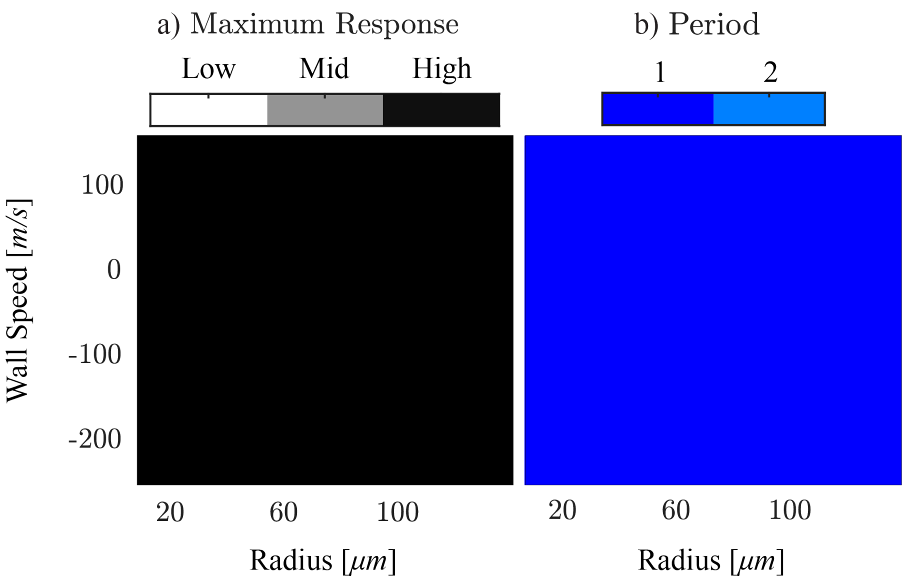

Figure S4.30: (a) the amplitude basins for bubble 6, where low-, mid- and high-amplitude attractor correspond to white, gray and black; and (b) the corresponding period basins of these attractors, as indicated by the colorbar.

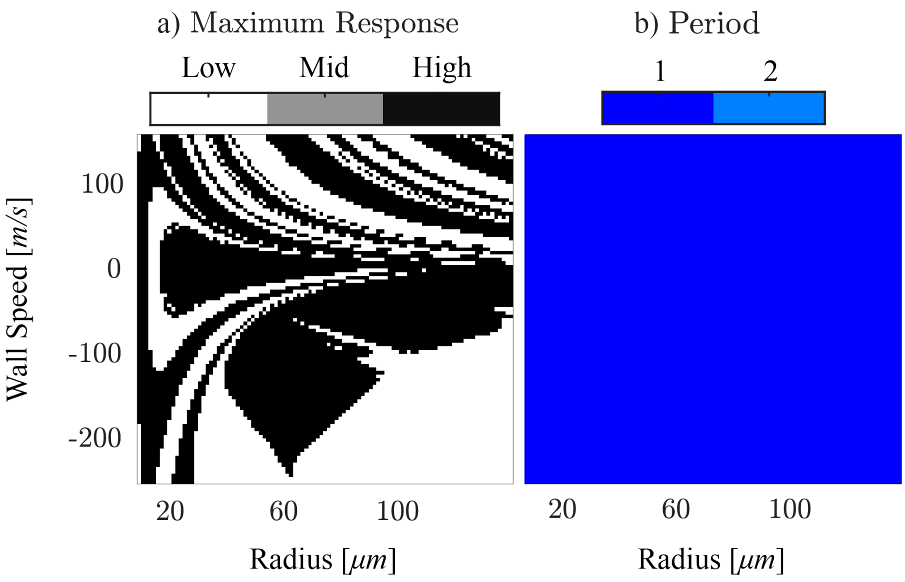

Figure S4.31: (a) the amplitude basins for bubble 7, where low-, mid- and high-amplitude attractor correspond to white, gray and black; and (b) the corresponding period basins of these attractors, as indicated by the colorbar.

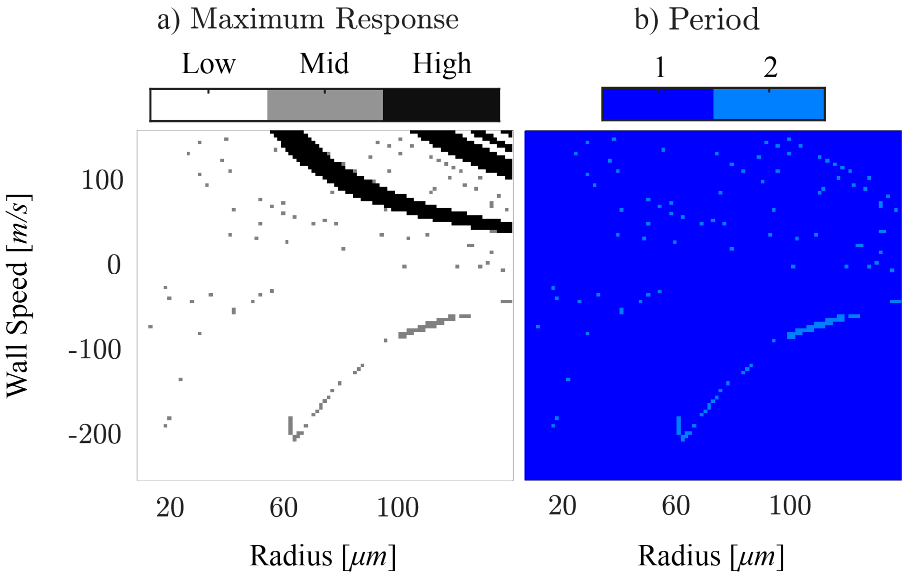

Figure S4.32: (a) the amplitude basins for bubble 8, where low-, mid- and high-amplitude attractor correspond to white, gray and black; and (b) the corresponding period basins of these attractors, as indicated by the colorbar.

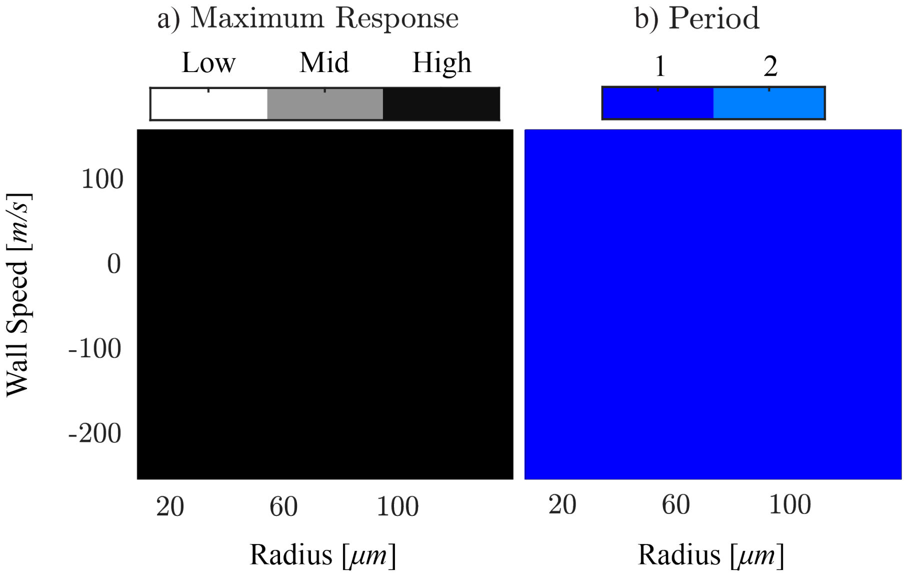

Figure S4.33: (a) the amplitude basins for bubble 9, where low-, mid- and high-amplitude attractor correspond to white, gray and black; and (b) the corresponding period basins of these attractors, as indicated by the colorbar.

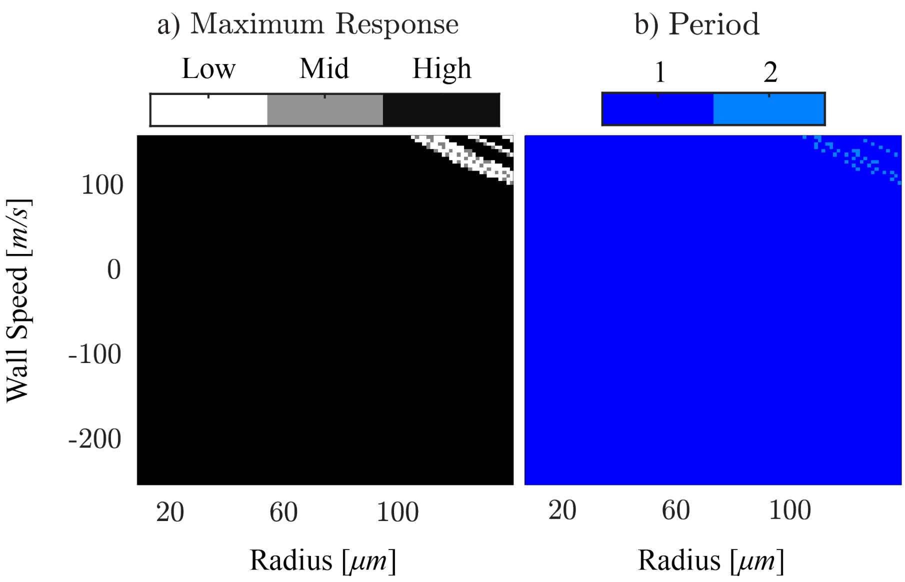

Figure S4.34: (a) the amplitude basins for bubble 10, where low-, mid- and high-amplitude attractor correspond to white, gray and black; and (b) the corresponding period basins of these attractors, as indicated by the colorbar.

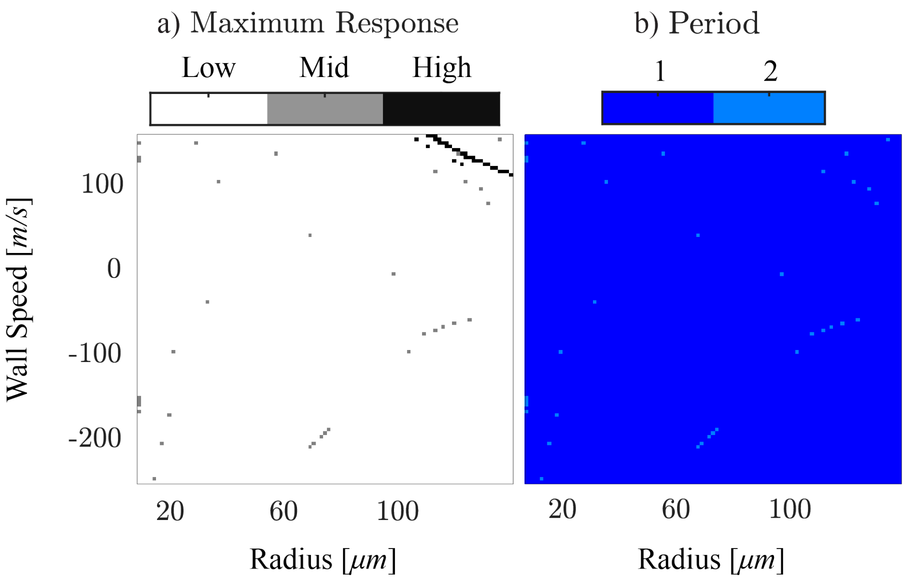

Figure S4.35: (a) the amplitude basins for bubble 11, where low-, mid- and high-amplitude attractor correspond to white, gray and black; and (b) the corresponding period basins of these attractors, as indicated by the colorbar.

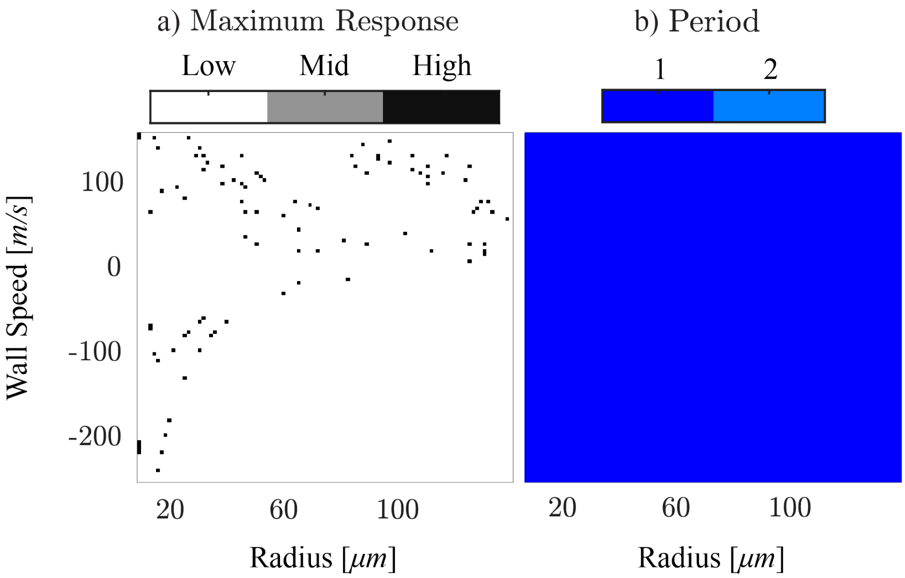

Figure S4.36: (a) the amplitude basins for bubble 12, where low-, mid- and high-amplitude attractor correspond to white, gray and black; and (b) the corresponding period basins of these attractors, as indicated by the colorbar.

Control bubble: Bubble 4

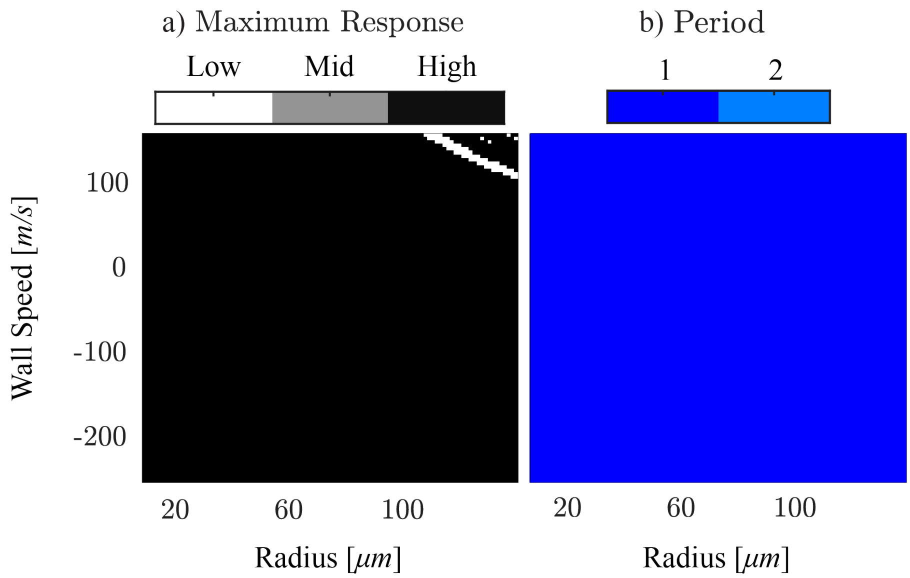

Figure S4.37: (a) the amplitude basins for bubble 1, where low-, mid- and high-amplitude attractor correspond to white, gray and black; and (b) the corresponding period basins of these attractors, as indicated by the colorbar.

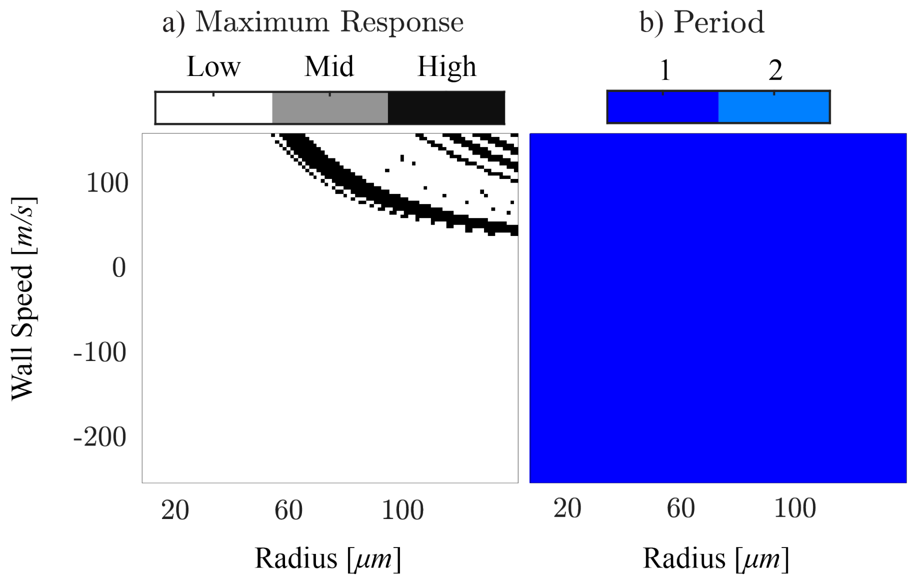

Figure S4.38: (a) the amplitude basins for bubble 2, where low-, mid- and high-amplitude attractor correspond to white, gray and black; and (b) the corresponding period basins of these attractors, as indicated by the colorbar.

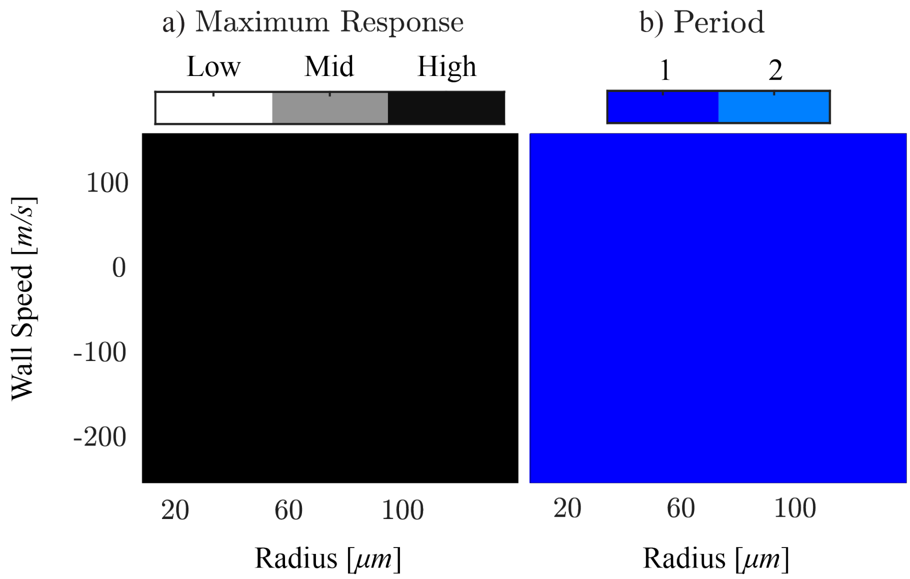

Figure S4.39: (a) the amplitude basins for bubble 3, where low-, mid- and high-amplitude attractor correspond to white, gray and black; and (b) the corresponding period basins of these attractors, as indicated by the colorbar.

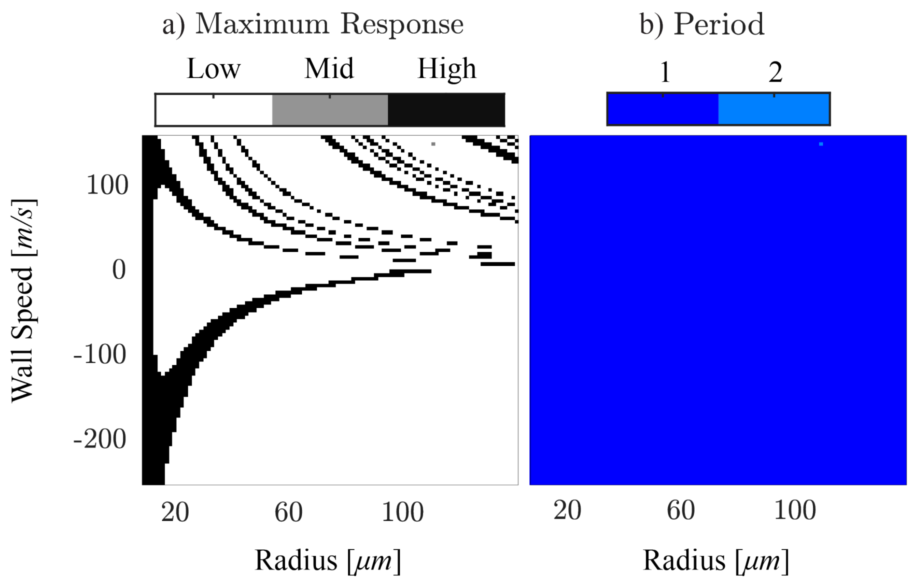

Figure S4.40: (a) the amplitude basins for bubble 4, where low-, mid- and high-amplitude attractor correspond to white, gray and black; and (b) the corresponding period basins of these attractors, as indicated by the colorbar.

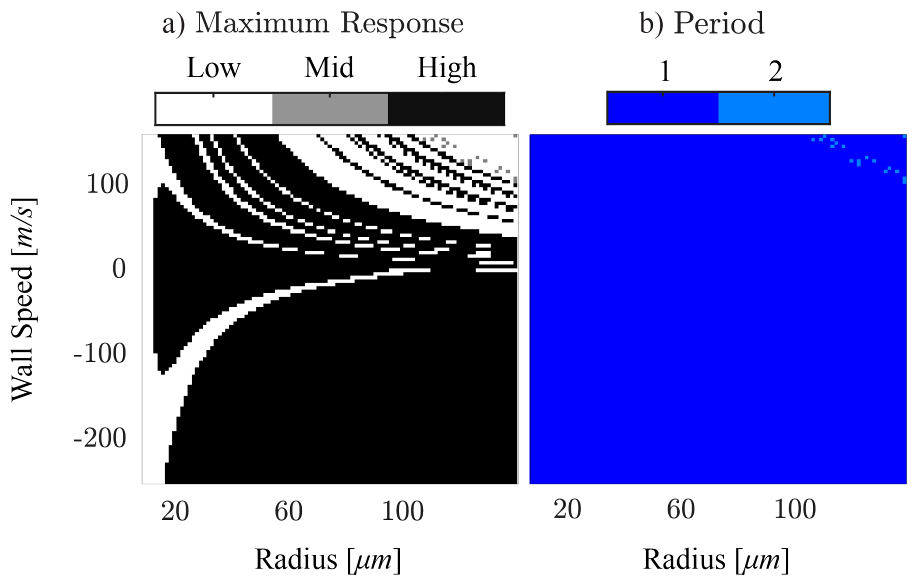

Figure S4.41: (a) the amplitude basins for bubble 5, where low-, mid- and high-amplitude attractor correspond to white, gray and black; and (b) the corresponding period basins of these attractors, as indicated by the colorbar.

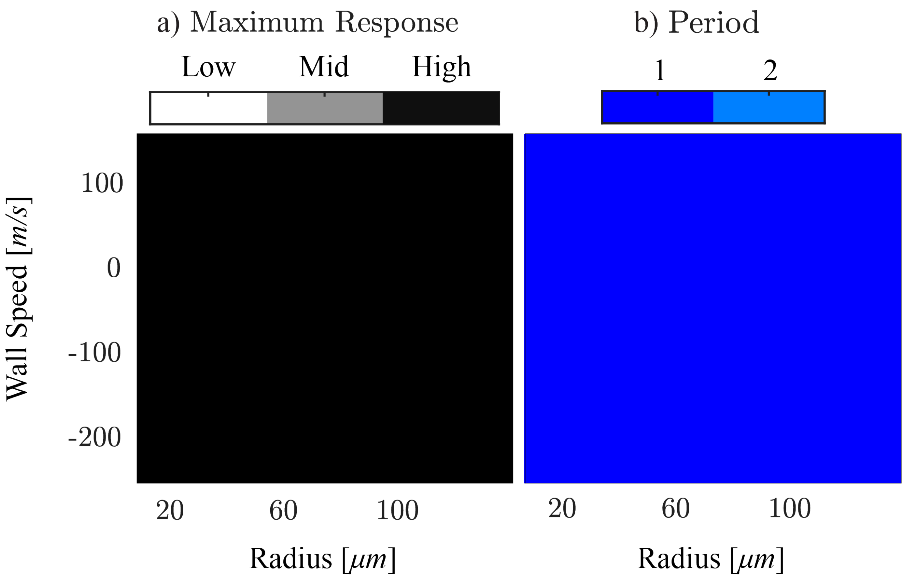

Figure S4.42: (a) the amplitude basins for bubble 6, where low-, mid- and high-amplitude attractor correspond to white, gray and black; and (b) the corresponding period basins of these attractors, as indicated by the colorbar.

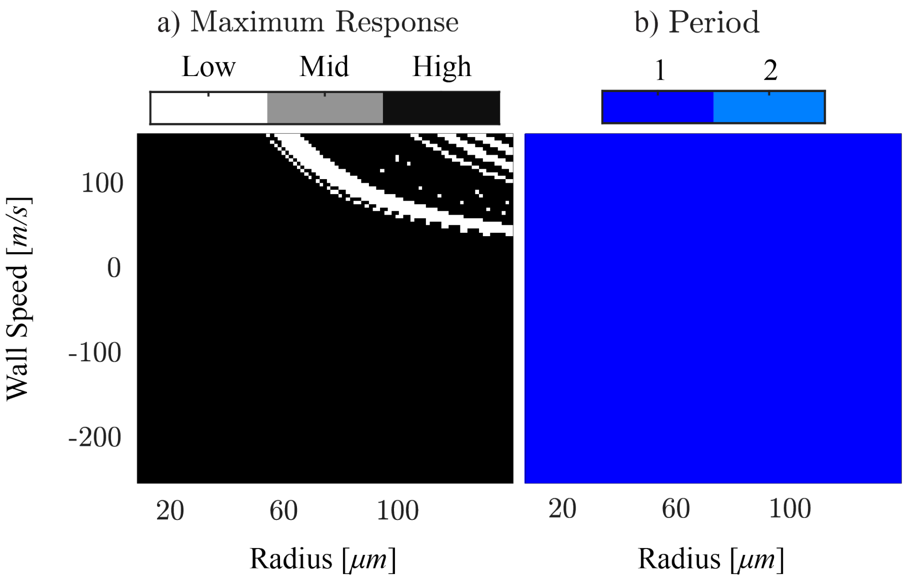

Figure S4.43: (a) the amplitude basins for bubble 7, where low-, mid- and high-amplitude attractor correspond to white, gray and black; and (b) the corresponding period basins of these attractors, as indicated by the colorbar.

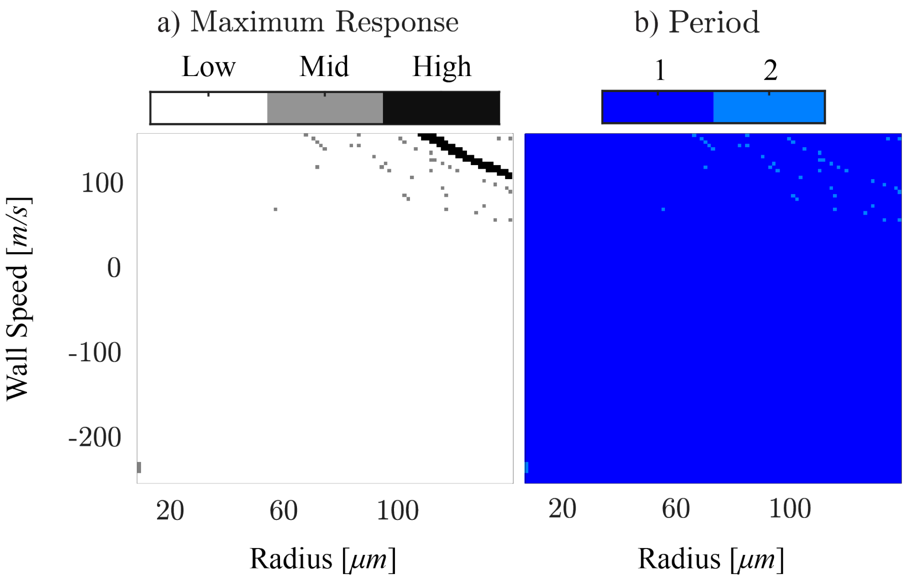

Figure S4.44: (a) the amplitude basins for bubble 8, where low-, mid- and high-amplitude attractor correspond to white, gray and black; and (b) the corresponding period basins of these attractors, as indicated by the colorbar.

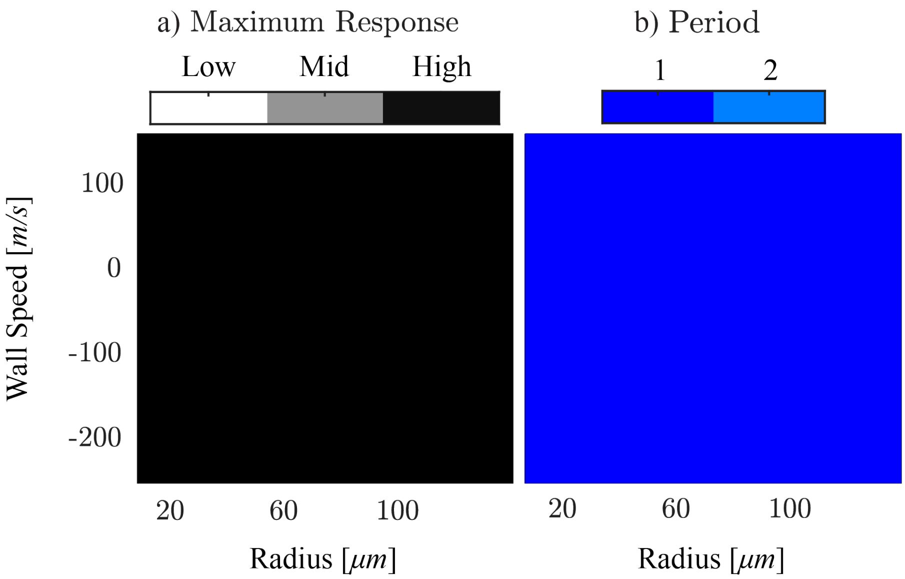

Figure S4.45: (a) the amplitude basins for bubble 9, where low-, mid- and high-amplitude attractor correspond to white, gray and black; and (b) the corresponding period basins of these attractors, as indicated by the colorbar.

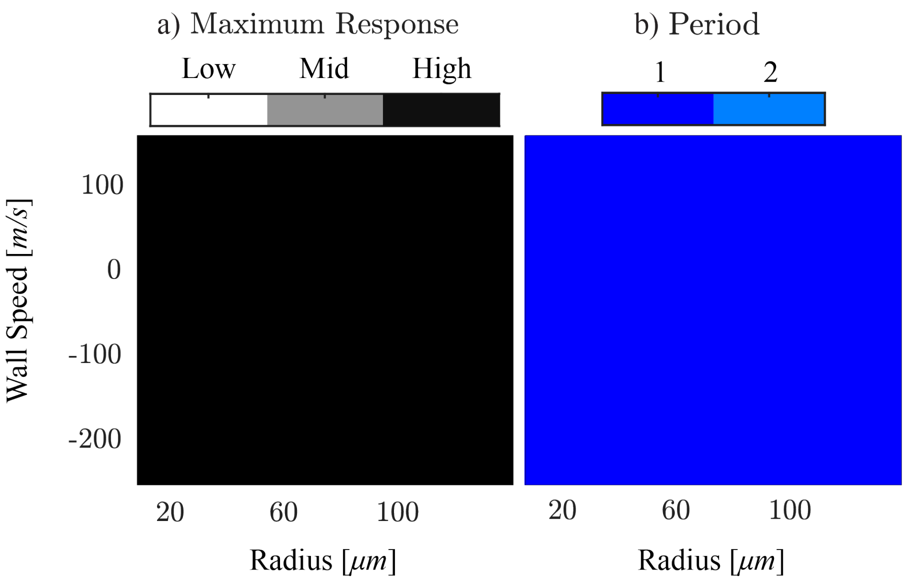

Figure S4.46: (a) the amplitude basins for bubble 10, where low-, mid- and high-amplitude attractor correspond to white, gray and black; and (b) the corresponding period basins of these attractors, as indicated by the colorbar.

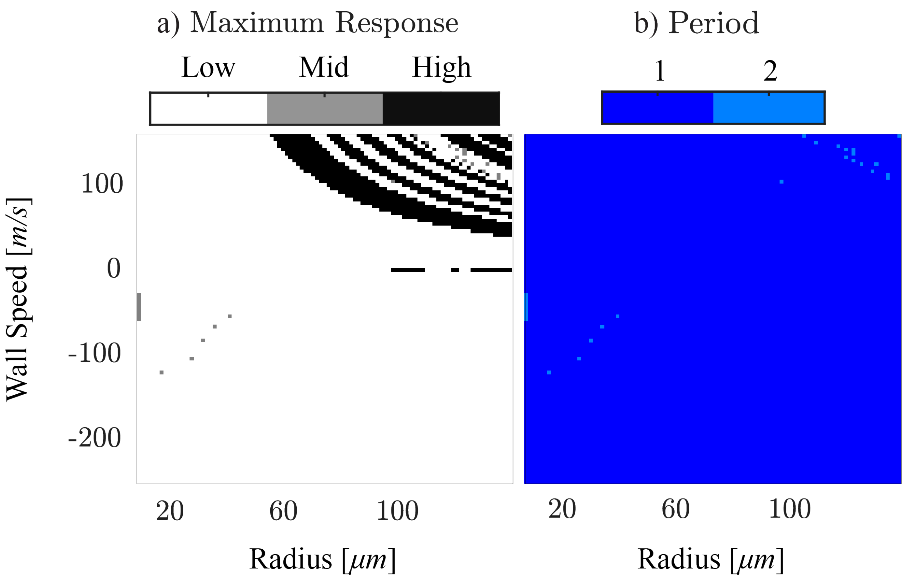

Figure S4.47: (a) the amplitude basins for bubble 11, where low-, mid- and high-amplitude attractor correspond to white, gray and black; and (b) the corresponding period basins of these attractors, as indicated by the colorbar.

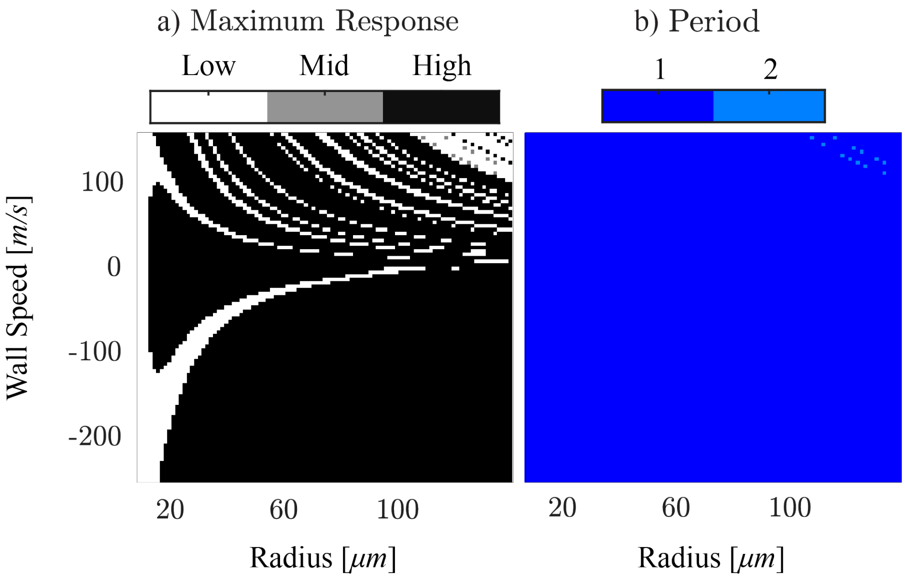

Figure S4.48: (a) the amplitude basins for bubble 12, where low-, mid- and high-amplitude attractor correspond to white, gray and black; and (b) the corresponding period basins of these attractors, as indicated by the colorbar.

Control bubble: Bubble 5

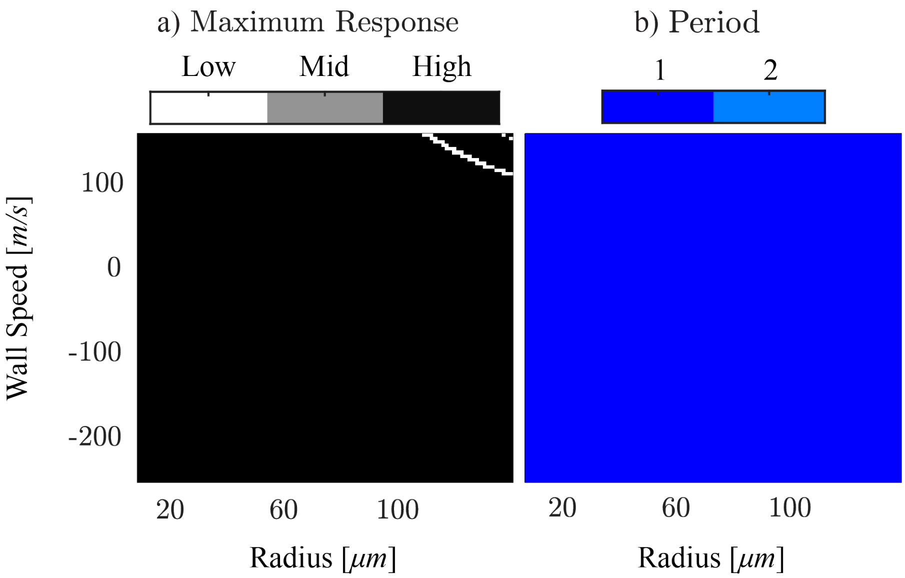

Figure S4.49: (a) the amplitude basins for bubble 1, where low-, mid- and high-amplitude attractor correspond to white, gray and black; and (b) the corresponding period basins of these attractors, as indicated by the colorbar.

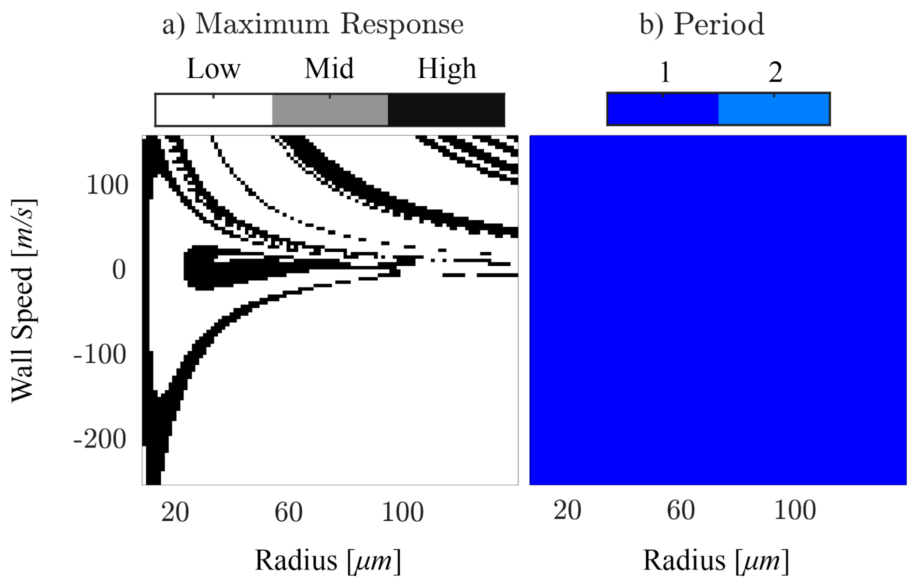

Figure S4.50: (a) the amplitude basins for bubble 2, where low-, mid- and high-amplitude attractor correspond to white, gray and black; and (b) the corresponding period basins of these attractors, as indicated by the colorbar.

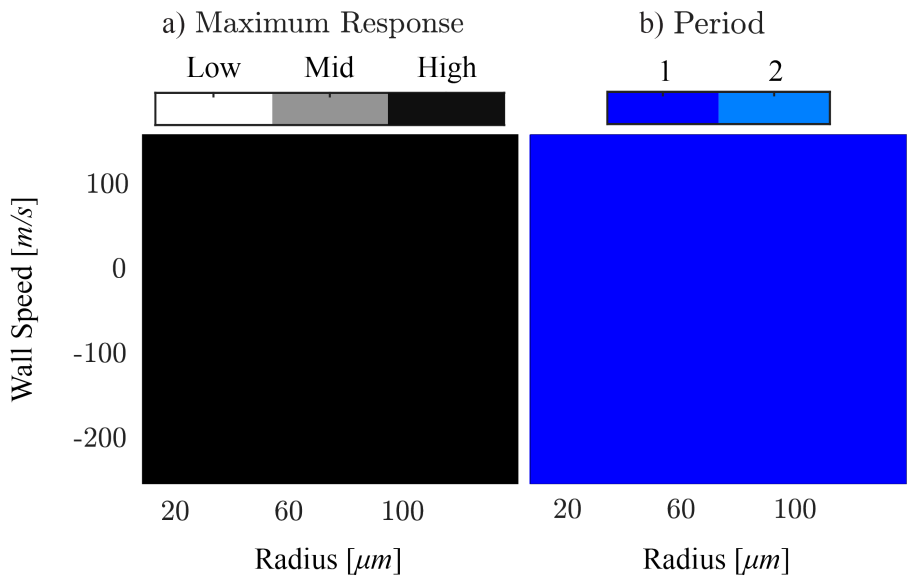

Figure S4.51: (a) the amplitude basins for bubble 3, where low-, mid- and high-amplitude attractor correspond to white, gray and black; and (b) the corresponding period basins of these attractors, as indicated by the colorbar.

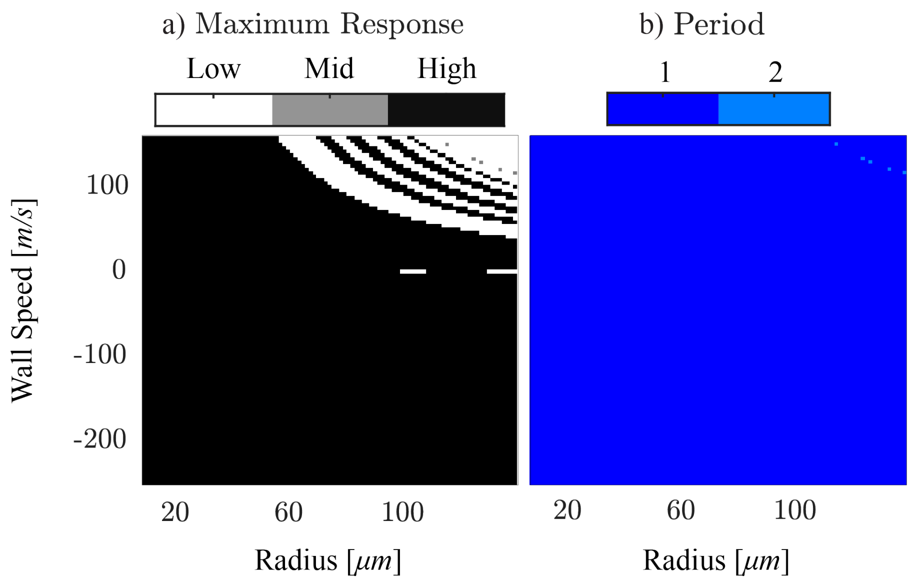

Figure S4.52: (a) the amplitude basins for bubble 4, where low-, mid- and high-amplitude attractor correspond to white, gray and black; and (b) the corresponding period basins of these attractors, as indicated by the colorbar.

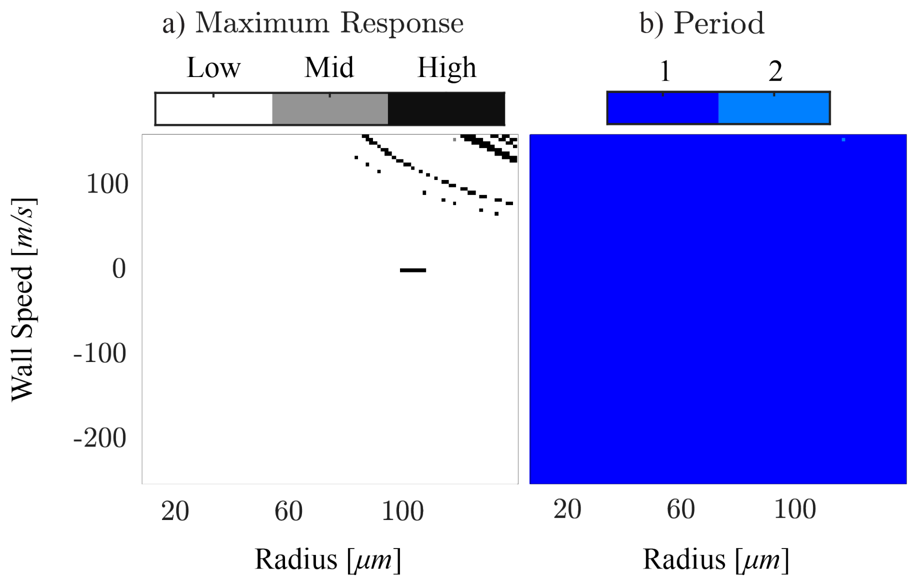

Figure S4.53: (a) the amplitude basins for bubble 5, where low-, mid- and high-amplitude attractor correspond to white, gray and black; and (b) the corresponding period basins of these attractors, as indicated by the colorbar.

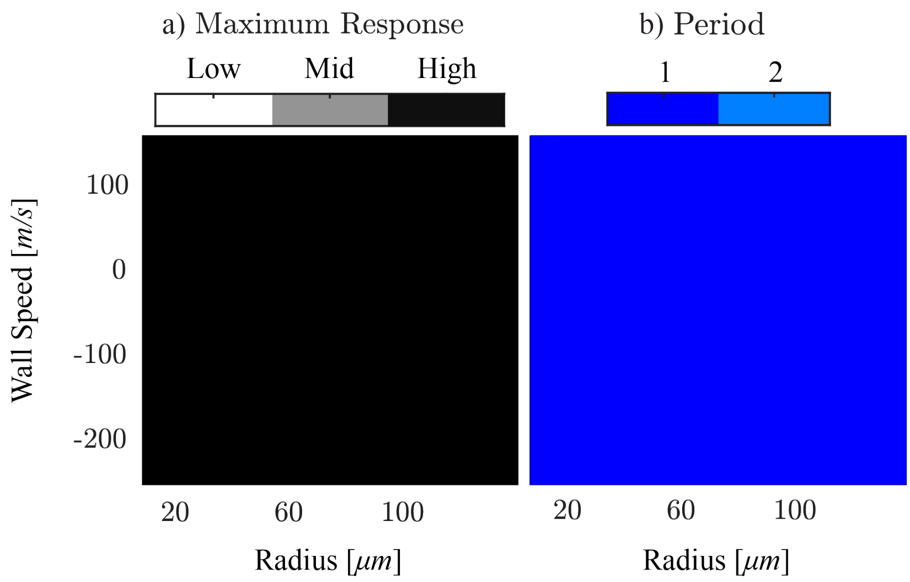

Figure S4.54: (a) the amplitude basins for bubble 6, where low-, mid- and high-amplitude attractor correspond to white, gray and black; and (b) the corresponding period basins of these attractors, as indicated by the colorbar.

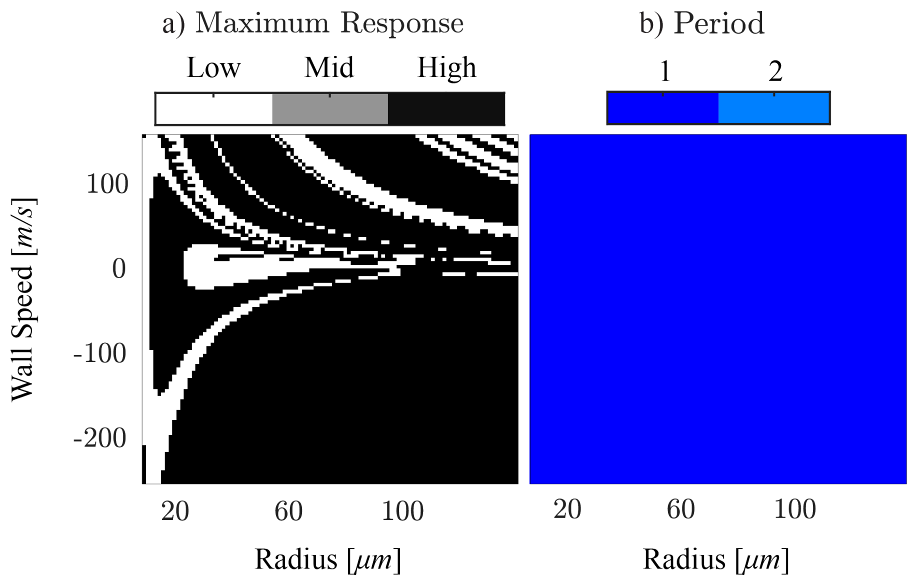

Figure S4.55: (a) the amplitude basins for bubble 7, where low-, mid- and high-amplitude attractor correspond to white, gray and black; and (b) the corresponding period basins of these attractors, as indicated by the colorbar.

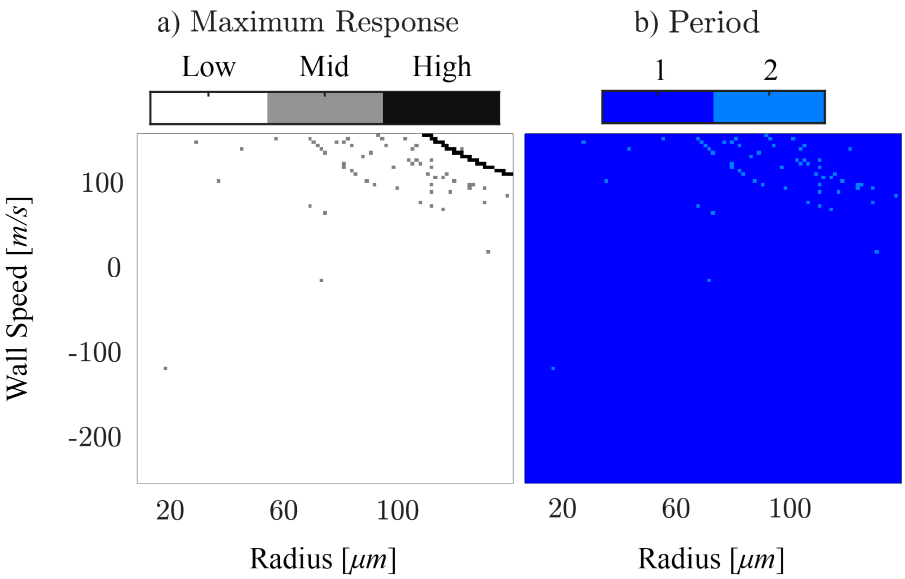

Figure S4.56: (a) the amplitude basins for bubble 8, where low-, mid- and high-amplitude attractor correspond to white, gray and black; and (b) the corresponding period basins of these attractors, as indicated by the colorbar.

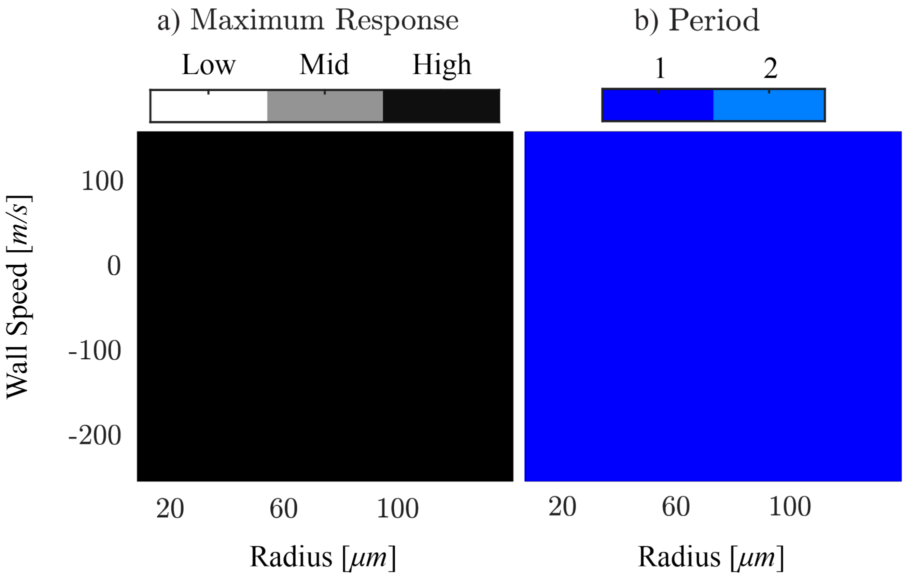

Figure S4.57: (a) the amplitude basins for bubble 9, where low-, mid- and high-amplitude attractor correspond to white, gray and black; and (b) the corresponding period basins of these attractors, as indicated by the colorbar.

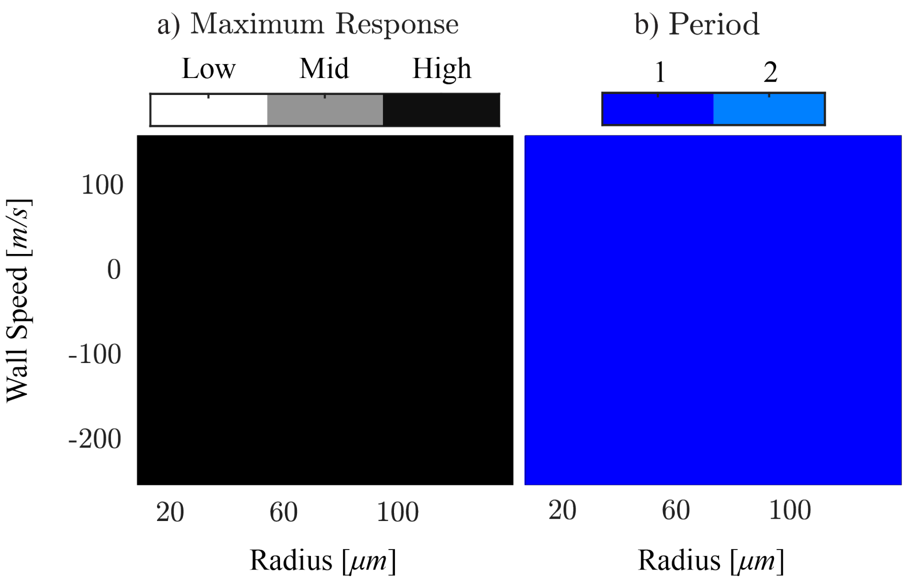

Figure S4.58: (a) the amplitude basins for bubble 10, where low-, mid- and high-amplitude attractor correspond to white, gray and black; and (b) the corresponding period basins of these attractors, as indicated by the colorbar.

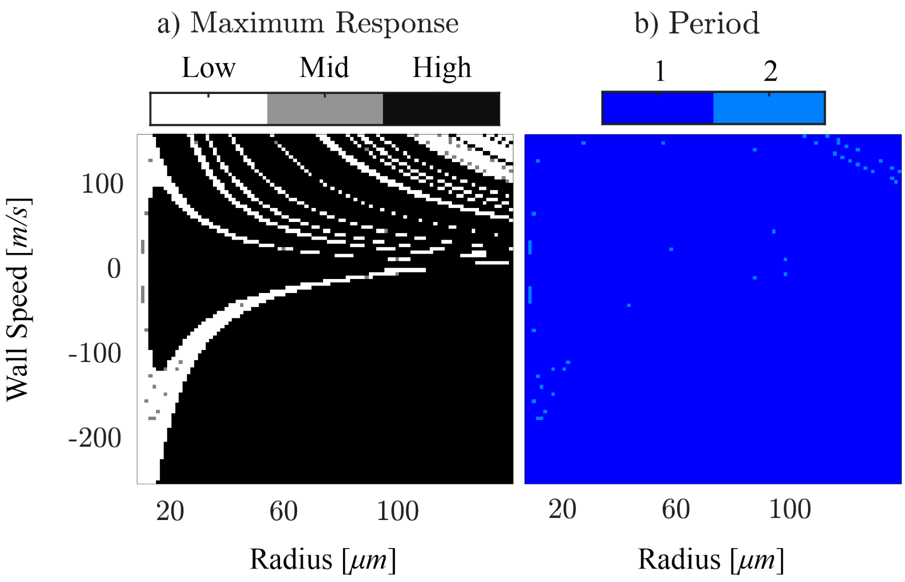

Figure S4.59: (a) the amplitude basins for bubble 11, where low-, mid- and high-amplitude attractor correspond to white, gray and black; and (b) the corresponding period basins of these attractors, as indicated by the colorbar.

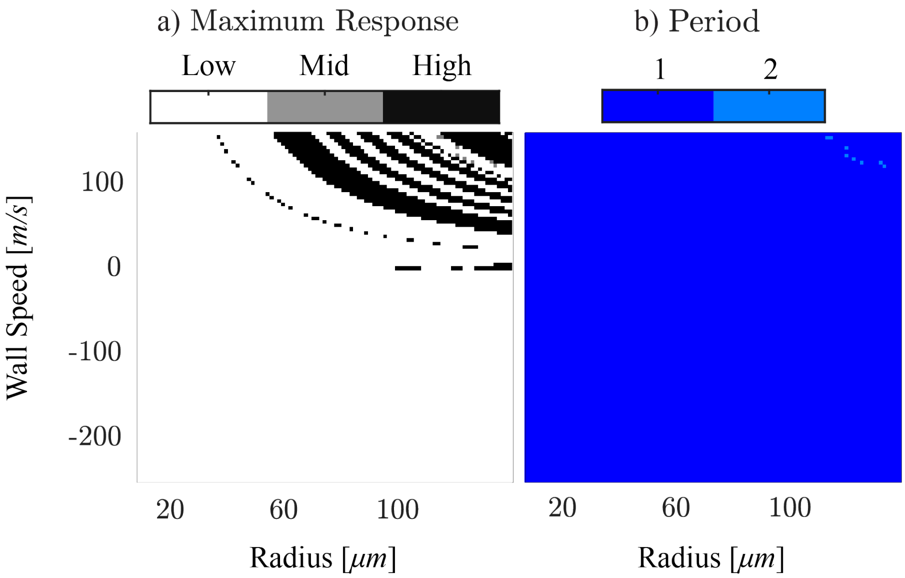

Figure S4.60: (a) the amplitude basins for bubble 12, where low-, mid- and high-amplitude attractor correspond to white, gray and black; and (b) the corresponding period basins of these attractors, as indicated by the colorbar.

Control bubble: Bubble 6

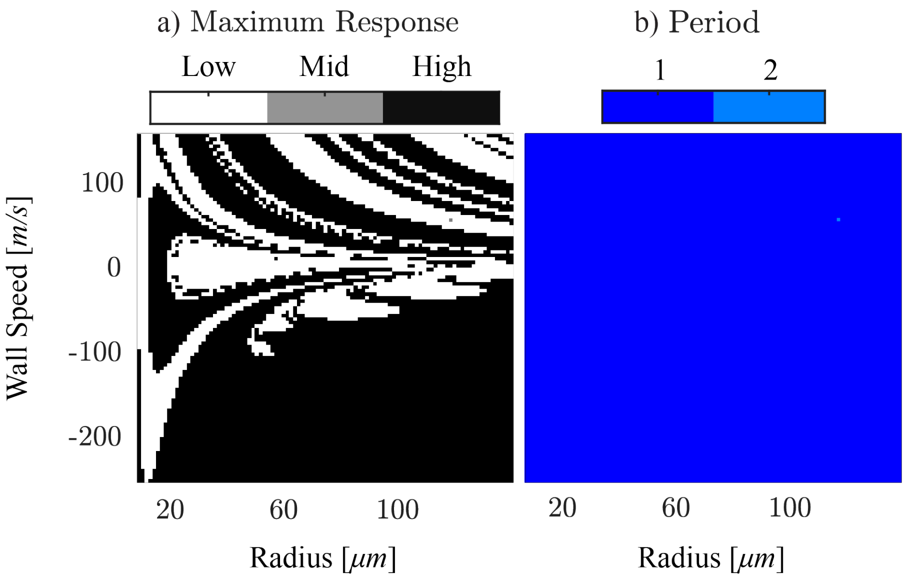

Figure S4.61: (a) the amplitude basins for bubble 1, where low-, mid- and high-amplitude attractor correspond to white, gray and black; and (b) the corresponding period basins of these attractors, as indicated by the colorbar.

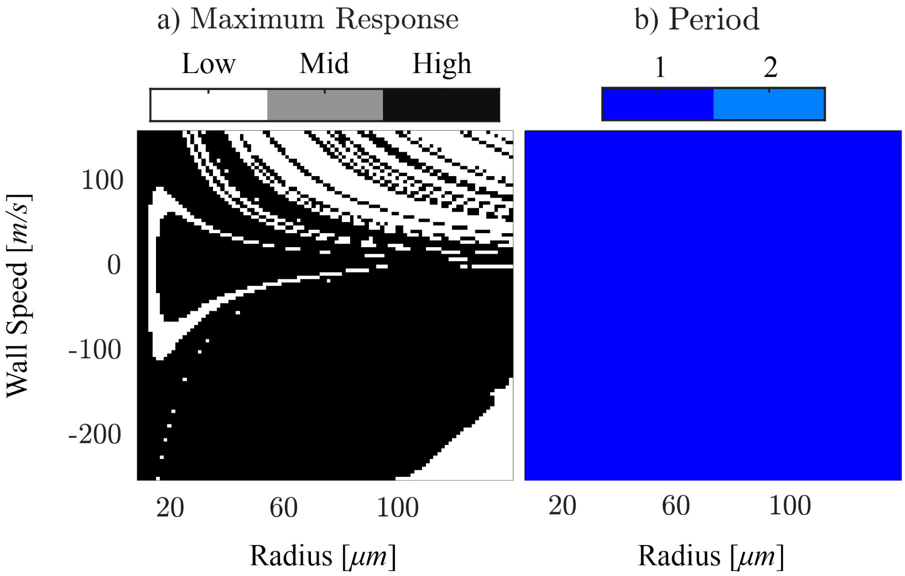

Figure S4.62: (a) the amplitude basins for bubble 2, where low-, mid- and high-amplitude attractor correspond to white, gray and black; and (b) the corresponding period basins of these attractors, as indicated by the colorbar.

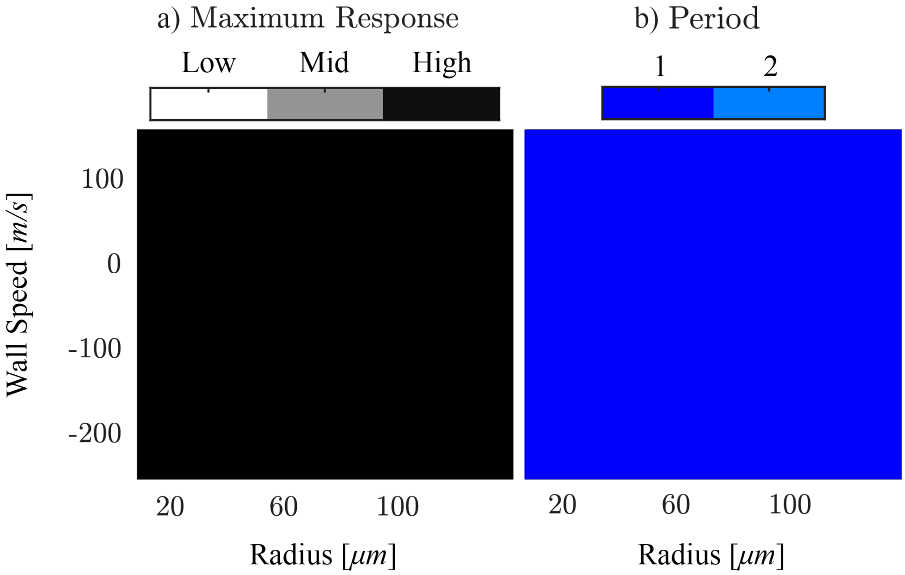

Figure S4.63: (a) the amplitude basins for bubble 3, where low-, mid- and high-amplitude attractor correspond to white, gray and black; and (b) the corresponding period basins of these attractors, as indicated by the colorbar.

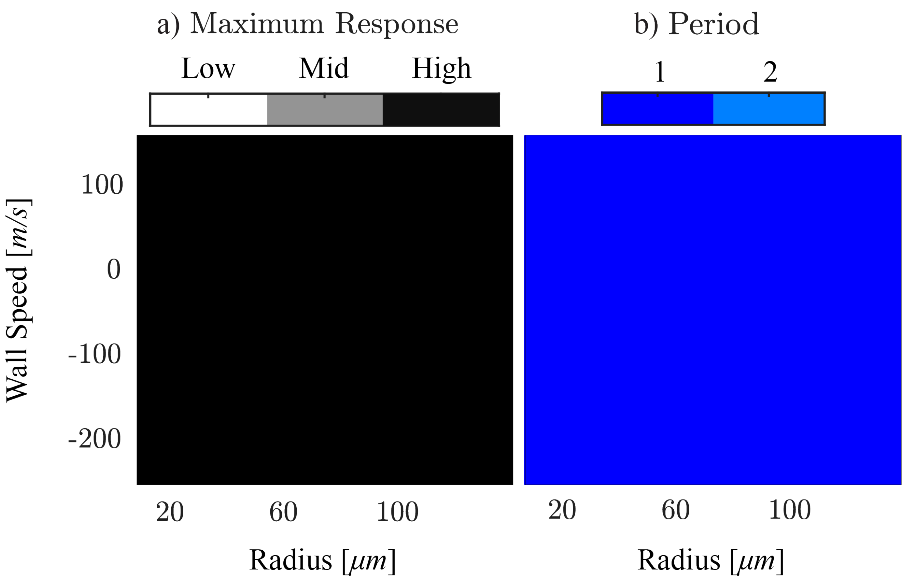

Figure S4.64: (a) the amplitude basins for bubble 4, where low-, mid- and high-amplitude attractor correspond to white, gray and black; and (b) the corresponding period basins of these attractors, as indicated by the colorbar.

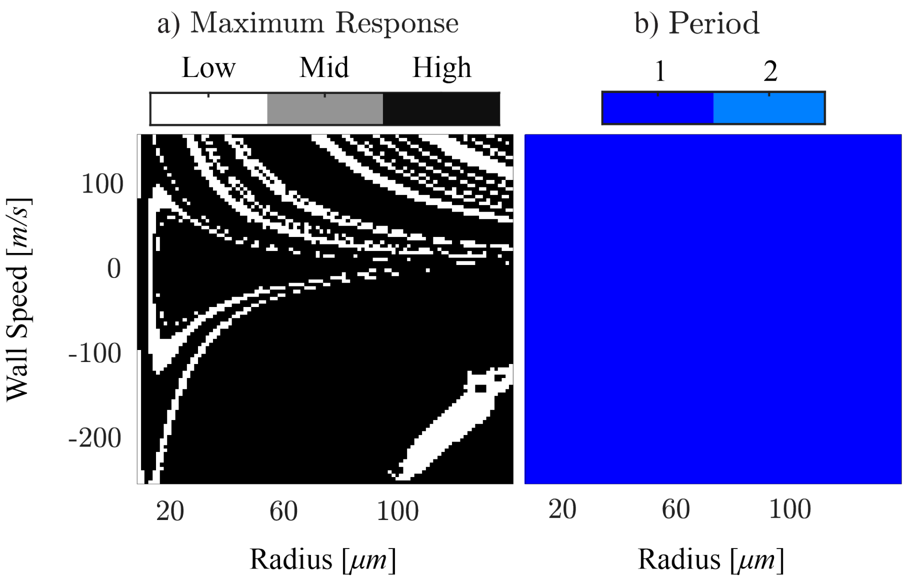

Figure S4.65: (a) the amplitude basins for bubble 5, where low-, mid- and high-amplitude attractor correspond to white, gray and black; and (b) the corresponding period basins of these attractors, as indicated by the colorbar.

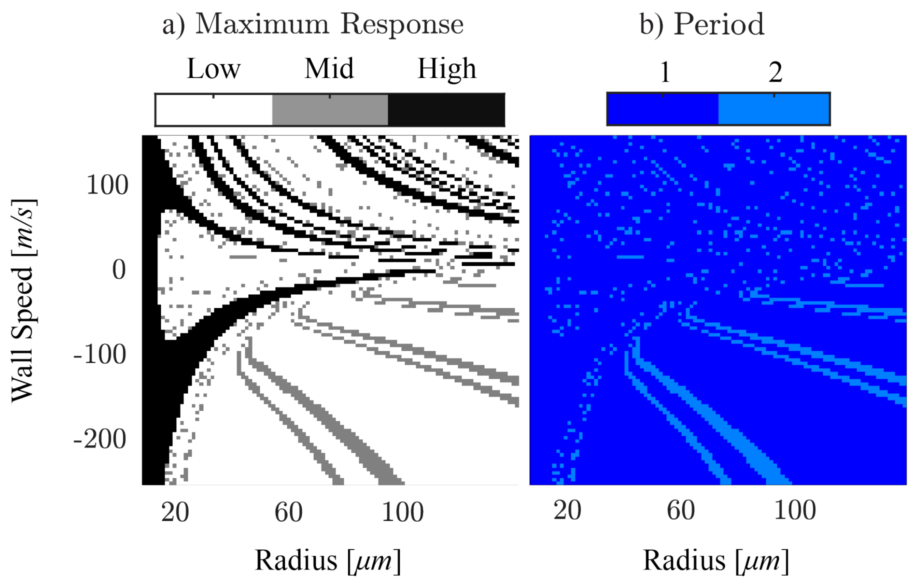

Figure S4.66: (a) the amplitude basins for bubble 6, where low-, mid- and high-amplitude attractor correspond to white, gray and black; and (b) the corresponding period basins of these attractors, as indicated by the colorbar.

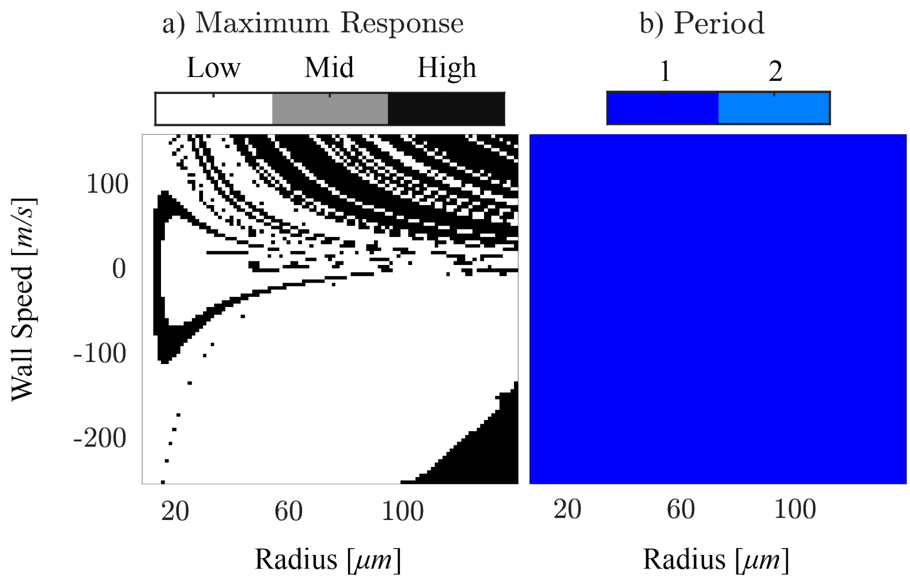

Figure S4.67: (a) the amplitude basins for bubble 7, where low-, mid- and high-amplitude attractor correspond to white, gray and black; and (b) the corresponding period basins of these attractors, as indicated by the colorbar.

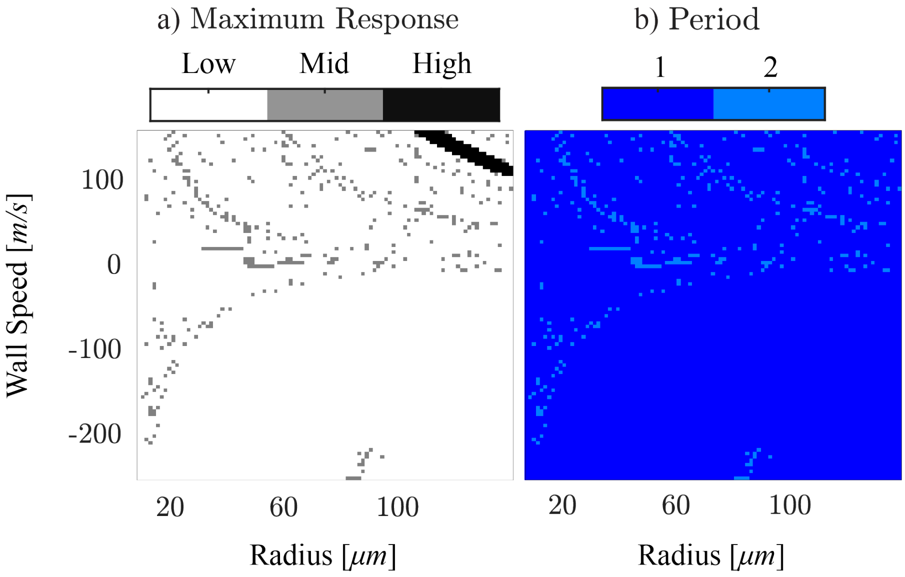

Figure S4.68: (a) the amplitude basins for bubble 8, where low-, mid- and high-amplitude attractor correspond to white, gray and black; and (b) the corresponding period basins of these attractors, as indicated by the colorbar.

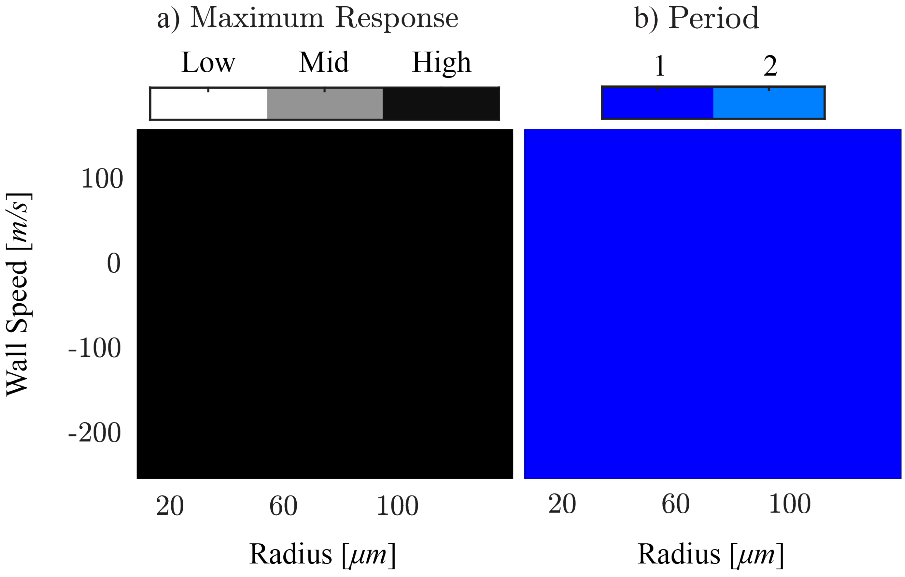

Figure S4.69: (a) the amplitude basins for bubble 9, where low-, mid- and high-amplitude attractor correspond to white, gray and black; and (b) the corresponding period basins of these attractors, as indicated by the colorbar.

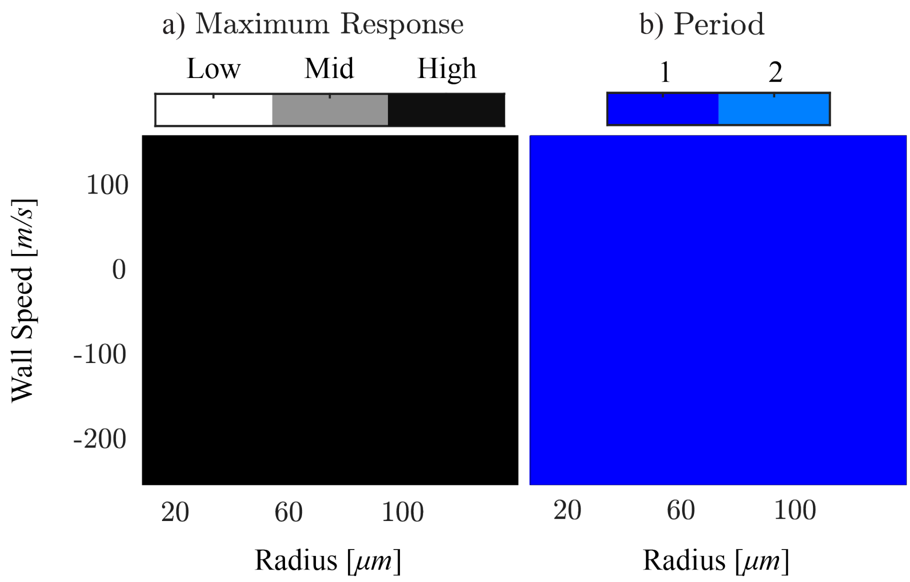

Figure S4.70: (a) the amplitude basins for bubble 10, where low-, mid- and high-amplitude attractor correspond to white, gray and black; and (b) the corresponding period basins of these attractors, as indicated by the colorbar.

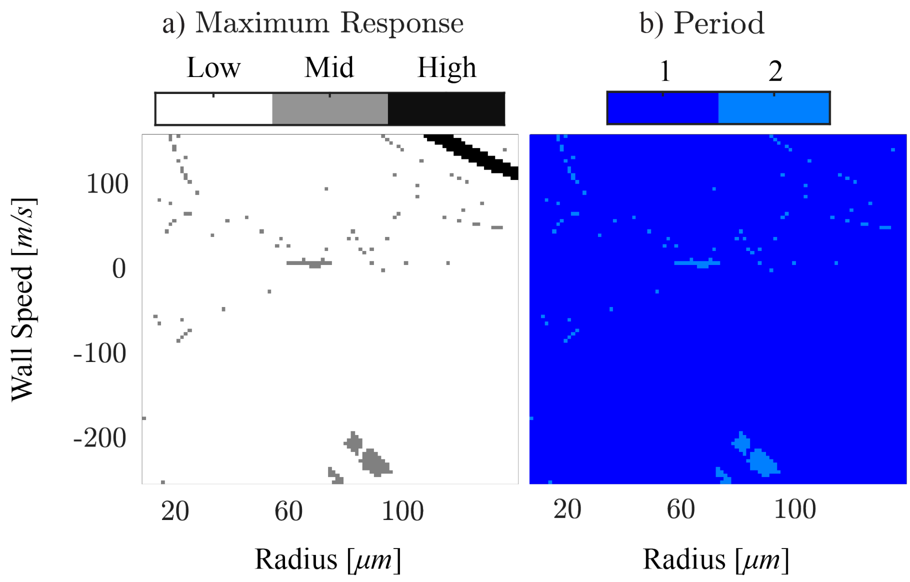

Figure S4.71: (a) the amplitude basins for bubble 11, where low-, mid- and high-amplitude attractor correspond to white, gray and black; and (b) the corresponding period basins of these attractors, as indicated by the colorbar.

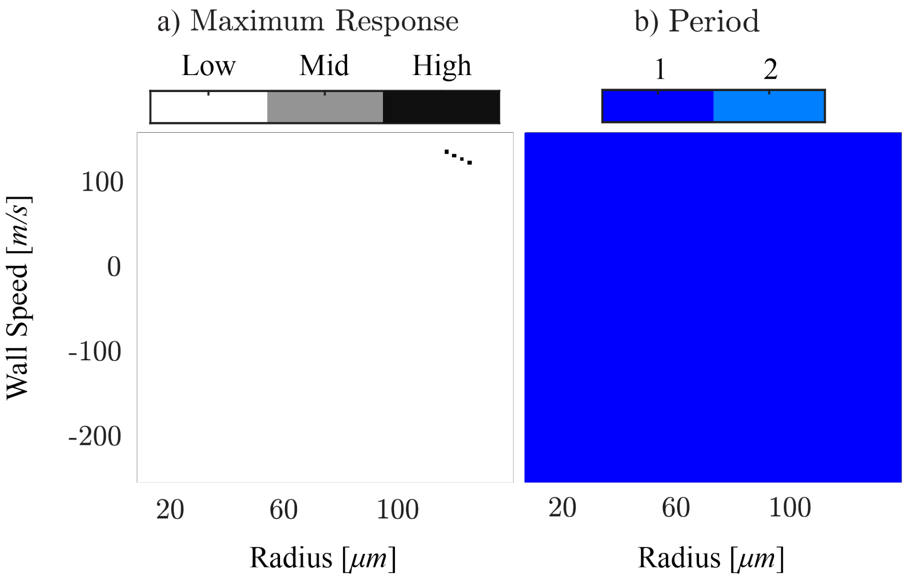

Figure S4.72: (a) the amplitude basins for bubble 12, where low-, mid- and high-amplitude attractor correspond to white, gray and black; and (b) the corresponding period basins of these attractors, as indicated by the colorbar.

Control bubble: Bubble 7

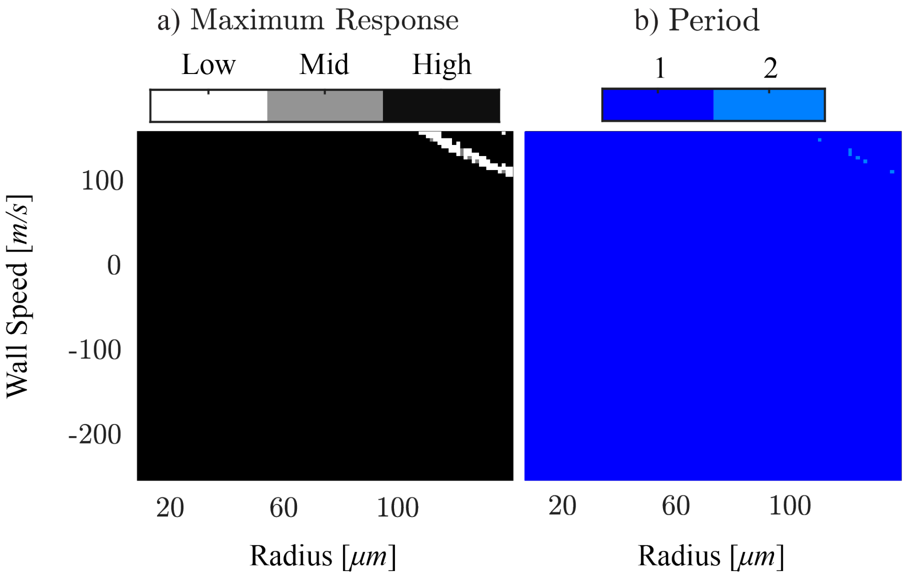

Figure S4.73: (a) the amplitude basins for bubble 1, where low-, mid- and high-amplitude attractor correspond to white, gray and black; and (b) the corresponding period basins of these attractors, as indicated by the colorbar.

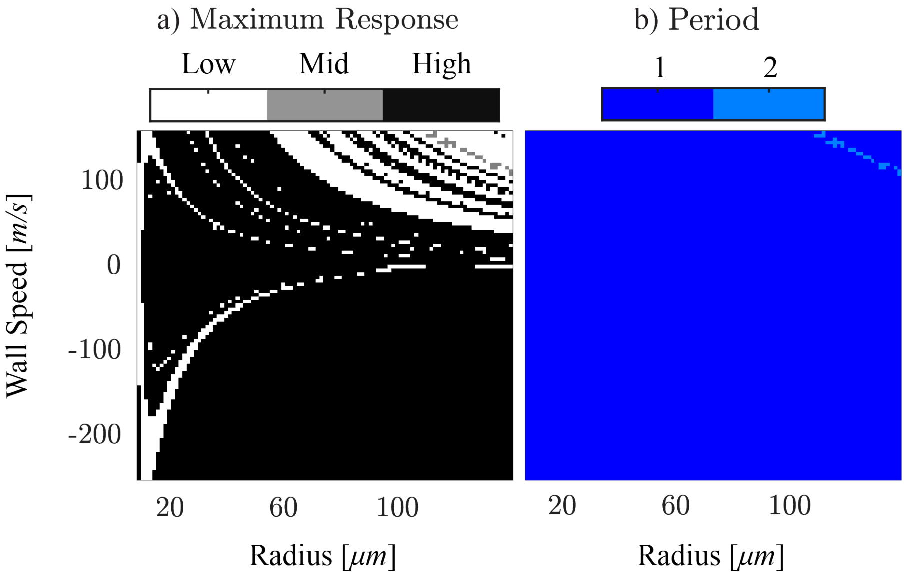

Figure S4.74: (a) the amplitude basins for bubble 2, where low-, mid- and high-amplitude attractor correspond to white, gray and black; and (b) the corresponding period basins of these attractors, as indicated by the colorbar.

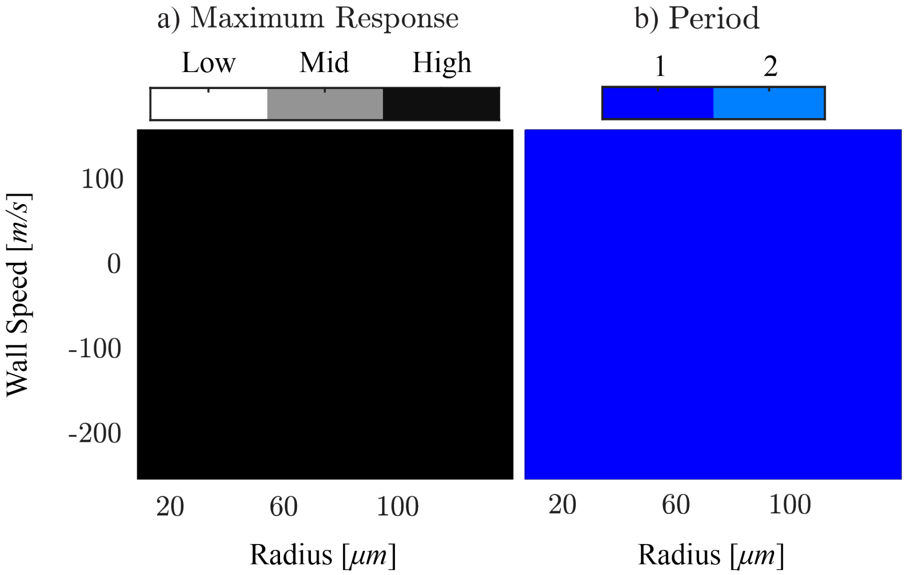

Figure S4.75: (a) the amplitude basins for bubble 3, where low-, mid- and high-amplitude attractor correspond to white, gray and black; and (b) the corresponding period basins of these attractors, as indicated by the colorbar.

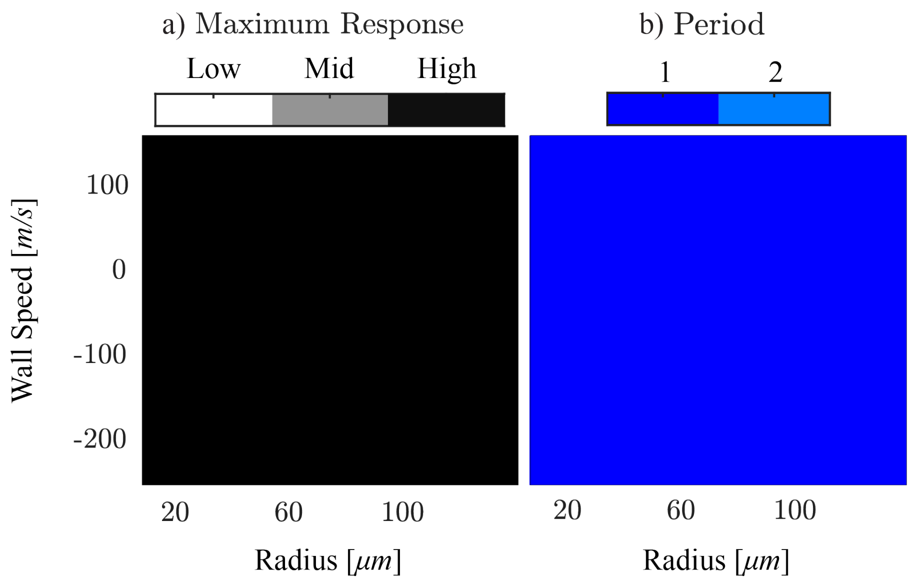

Figure S4.76: (a) the amplitude basins for bubble 4, where low-, mid- and high-amplitude attractor correspond to white, gray and black; and (b) the corresponding period basins of these attractors, as indicated by the colorbar.

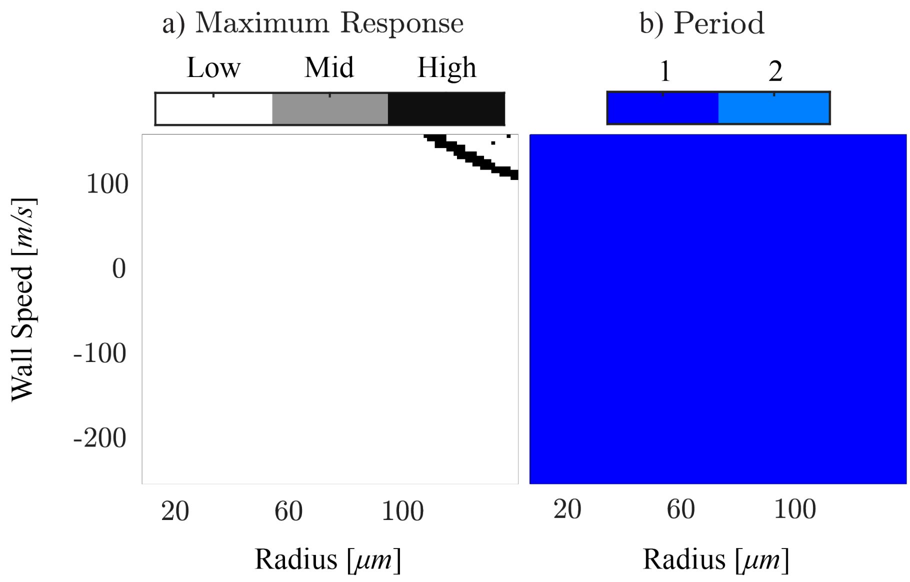

Figure S4.77: (a) the amplitude basins for bubble 5, where low-, mid- and high-amplitude attractor correspond to white, gray and black; and (b) the corresponding period basins of these attractors, as indicated by the colorbar.

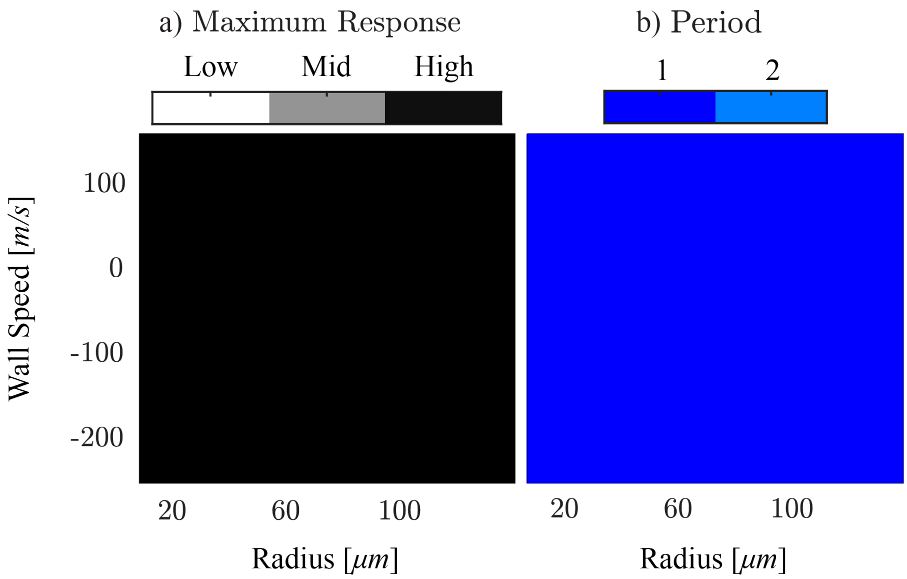

Figure S4.78: (a) the amplitude basins for bubble 6, where low-, mid- and high-amplitude attractor correspond to white, gray and black; and (b) the corresponding period basins of these attractors, as indicated by the colorbar.

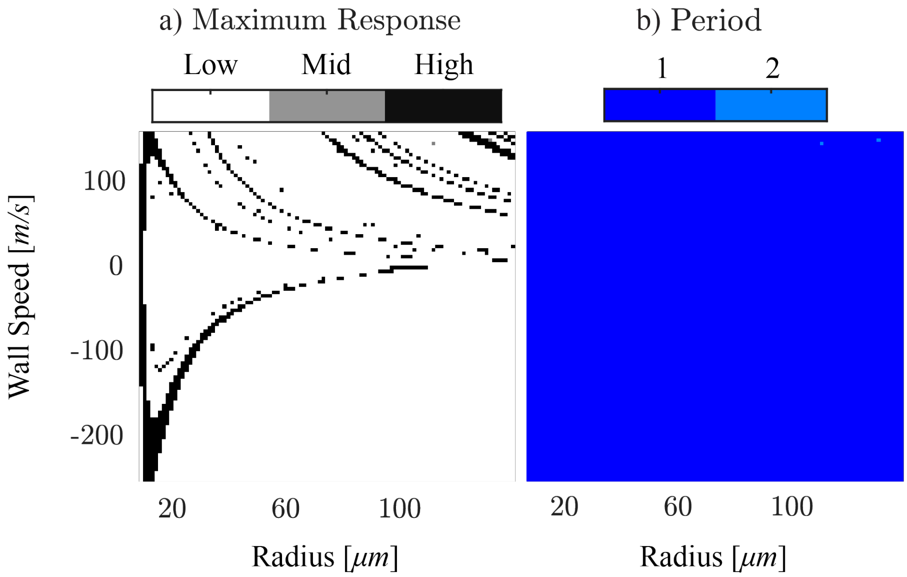

Figure S4.79: (a) the amplitude basins for bubble 7, where low-, mid- and high-amplitude attractor correspond to white, gray and black; and (b) the corresponding period basins of these attractors, as indicated by the colorbar.

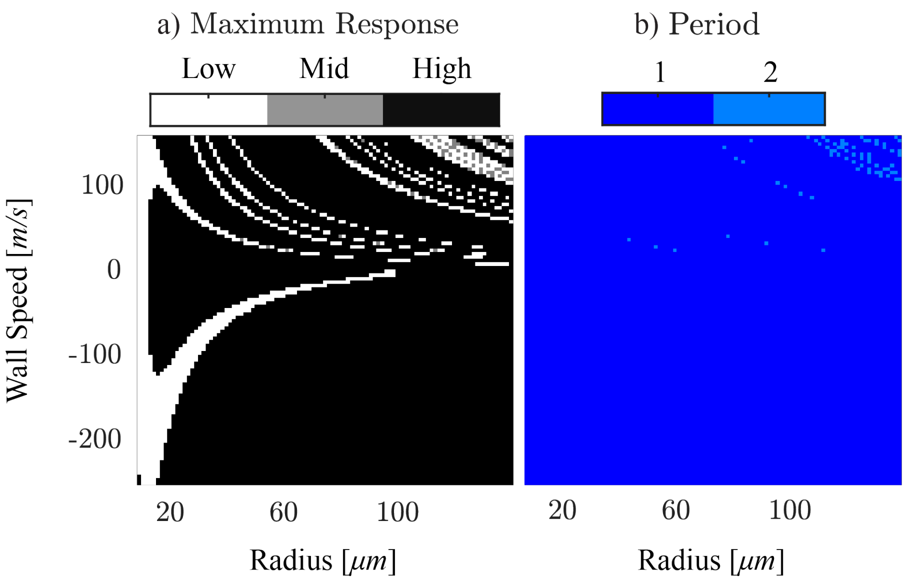

Figure S4.80: (a) the amplitude basins for bubble 8, where low-, mid- and high-amplitude attractor correspond to white, gray and black; and (b) the corresponding period basins of these attractors, as indicated by the colorbar.

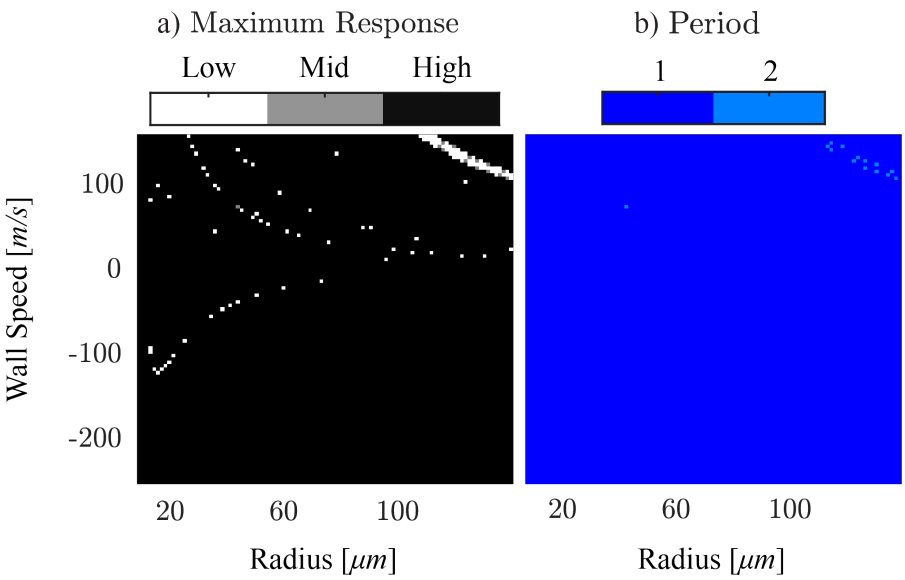

Figure S4.81: (a) the amplitude basins for bubble 9, where low-, mid- and high-amplitude attractor correspond to white, gray and black; and (b) the corresponding period basins of these attractors, as indicated by the colorbar.

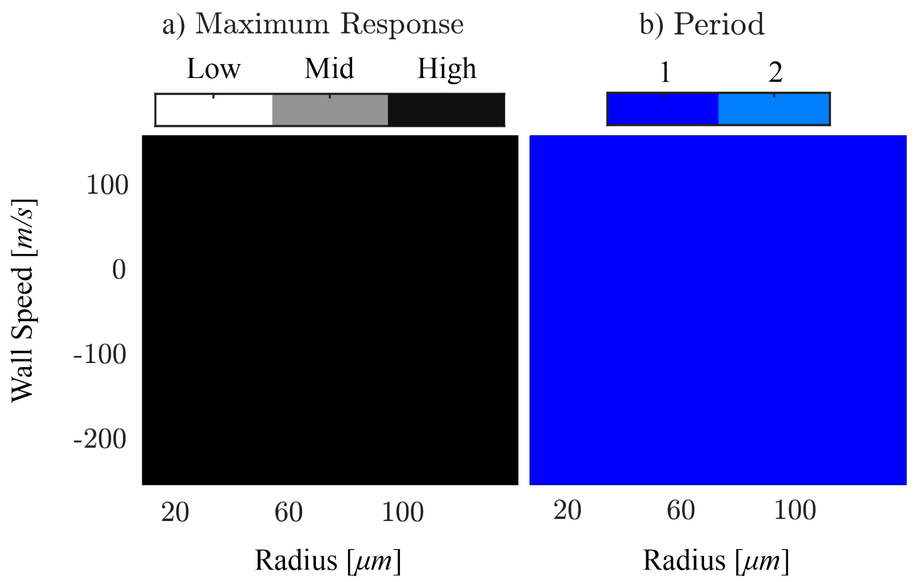

Figure S4.82: (a) the amplitude basins for bubble 10, where low-, mid- and high-amplitude attractor correspond to white, gray and black; and (b) the corresponding period basins of these attractors, as indicated by the colorbar.

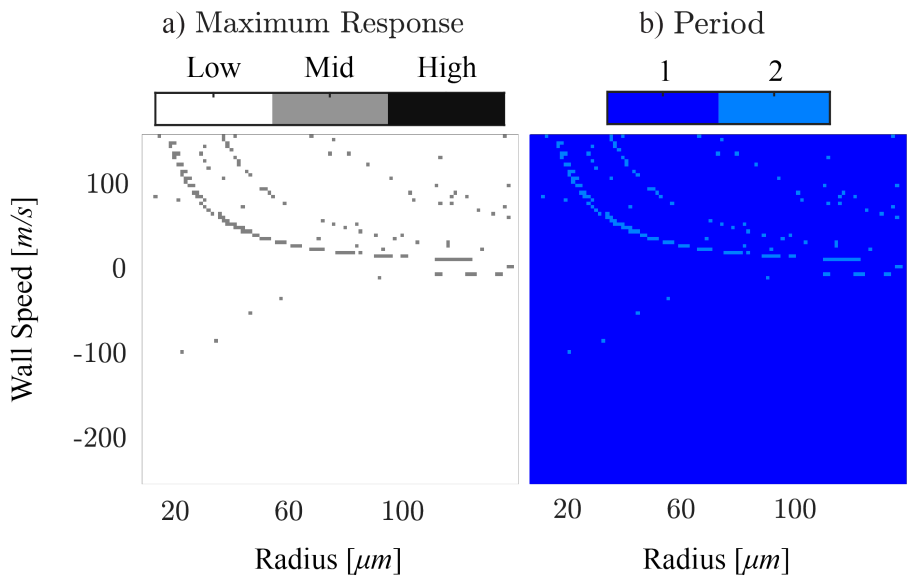

Figure S4.83: (a) the amplitude basins for bubble 11, where low-, mid- and high-amplitude attractor correspond to white, gray and black; and (b) the corresponding period basins of these attractors, as indicated by the colorbar.

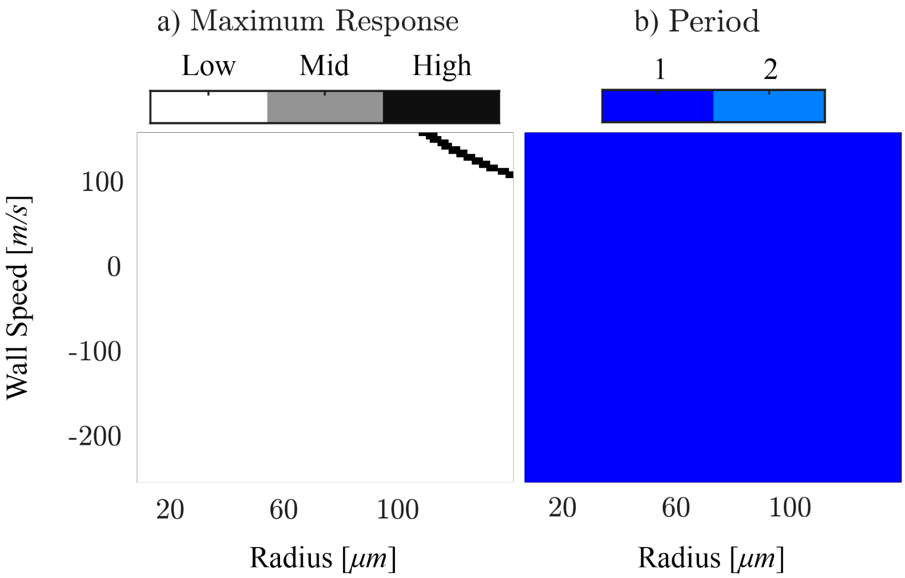

Figure S4.84: (a) the amplitude basins for bubble 12, where low-, mid- and high-amplitude attractor correspond to white, gray and black; and (b) the corresponding period basins of these attractors, as indicated by the colorbar.

Control bubble: Bubble 8

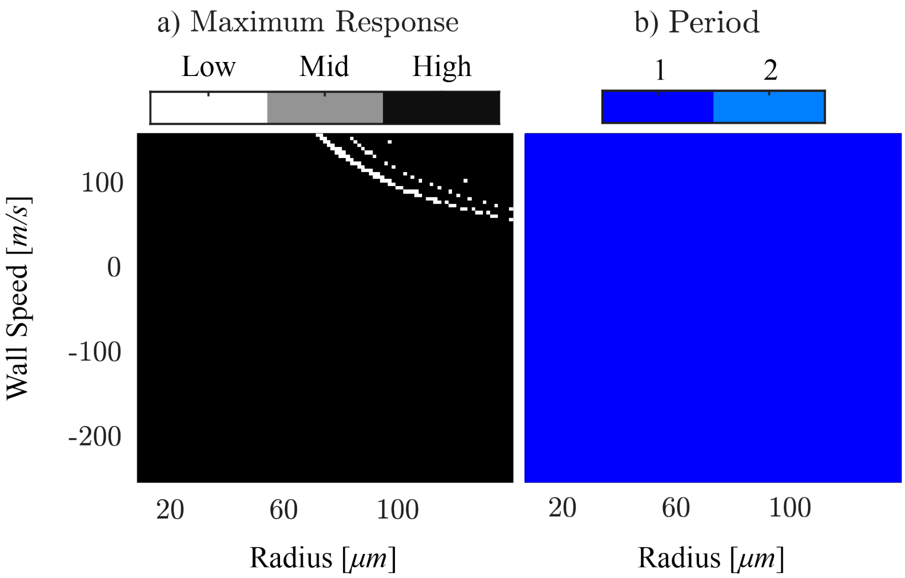

Figure S4.85: (a) the amplitude basins for bubble 1, where low-, mid- and high-amplitude attractor correspond to white, gray and black; and (b) the corresponding period basins of these attractors, as indicated by the colorbar.

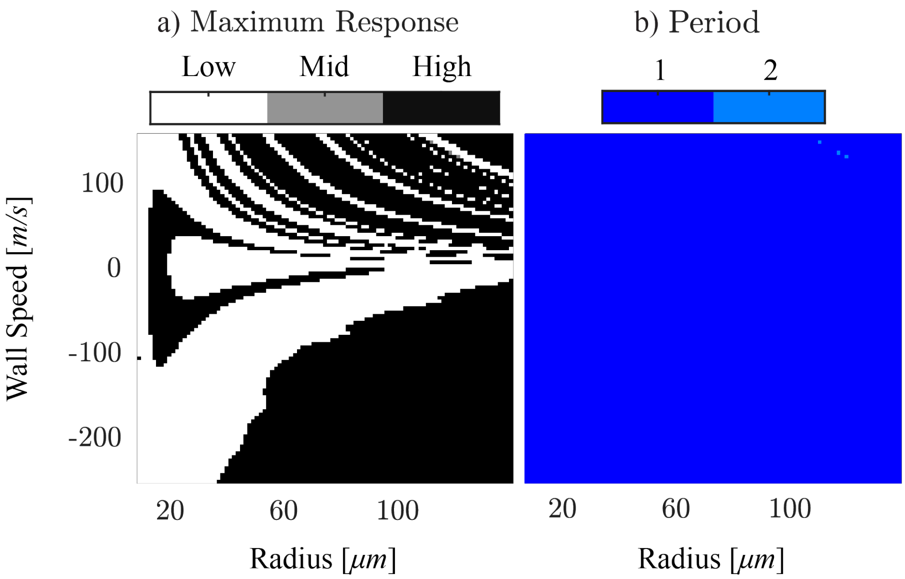

Figure S4.86: (a) the amplitude basins for bubble 2, where low-, mid- and high-amplitude attractor correspond to white, gray and black; and (b) the corresponding period basins of these attractors, as indicated by the colorbar.

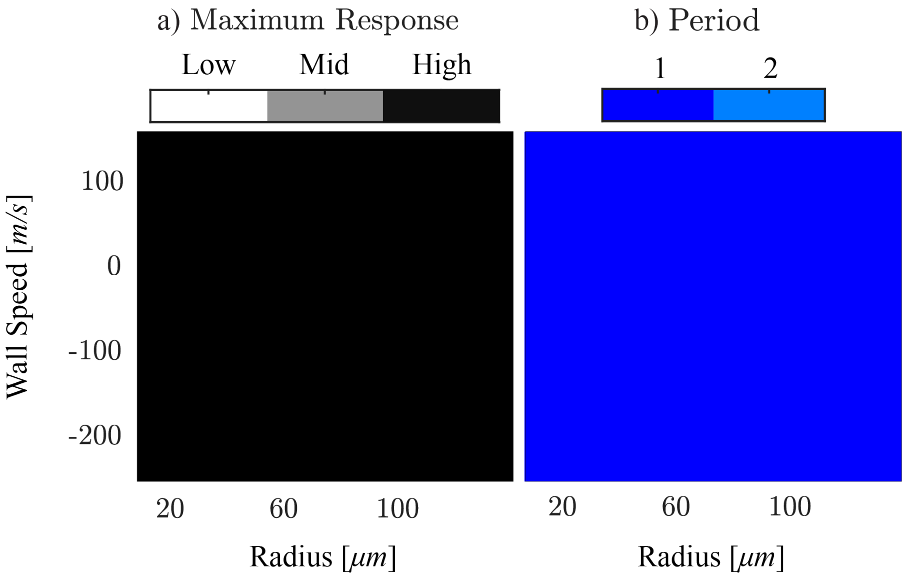

Figure S4.87: (a) the amplitude basins for bubble 3, where low-, mid- and high-amplitude attractor correspond to white, gray and black; and (b) the corresponding period basins of these attractors, as indicated by the colorbar.

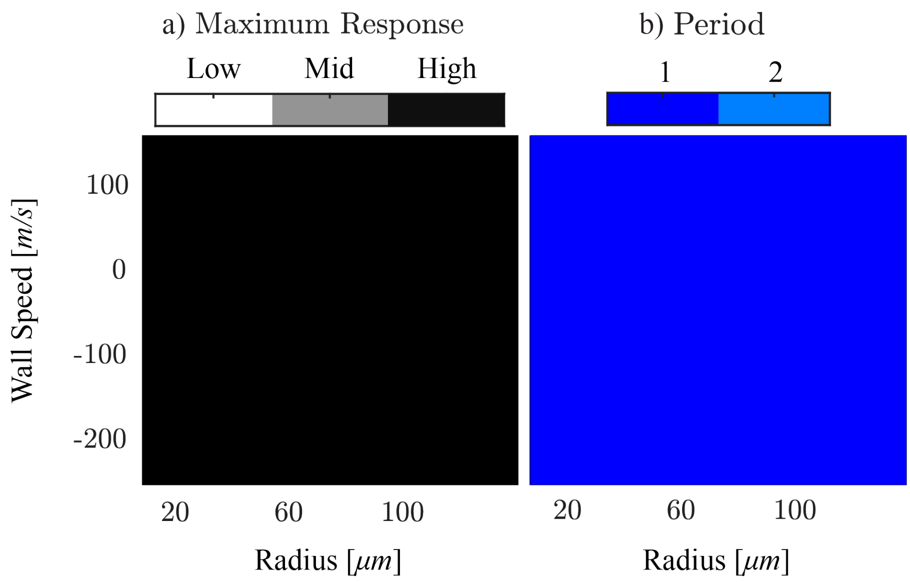

Figure S4.88: (a) the amplitude basins for bubble 4, where low-, mid- and high-amplitude attractor correspond to white, gray and black; and (b) the corresponding period basins of these attractors, as indicated by the colorbar.

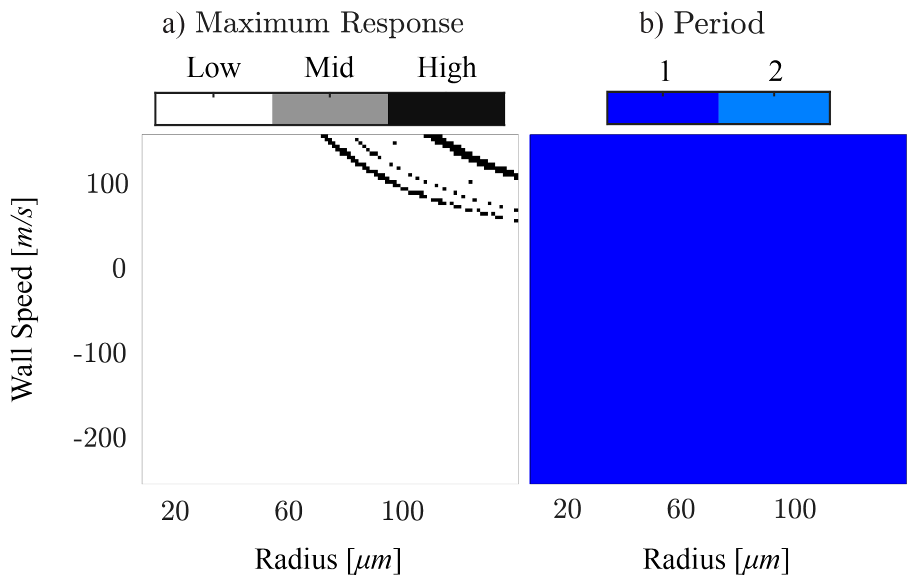

Figure S4.89: (a) the amplitude basins for bubble 5, where low-, mid- and high-amplitude attractor correspond to white, gray and black; and (b) the corresponding period basins of these attractors, as indicated by the colorbar.

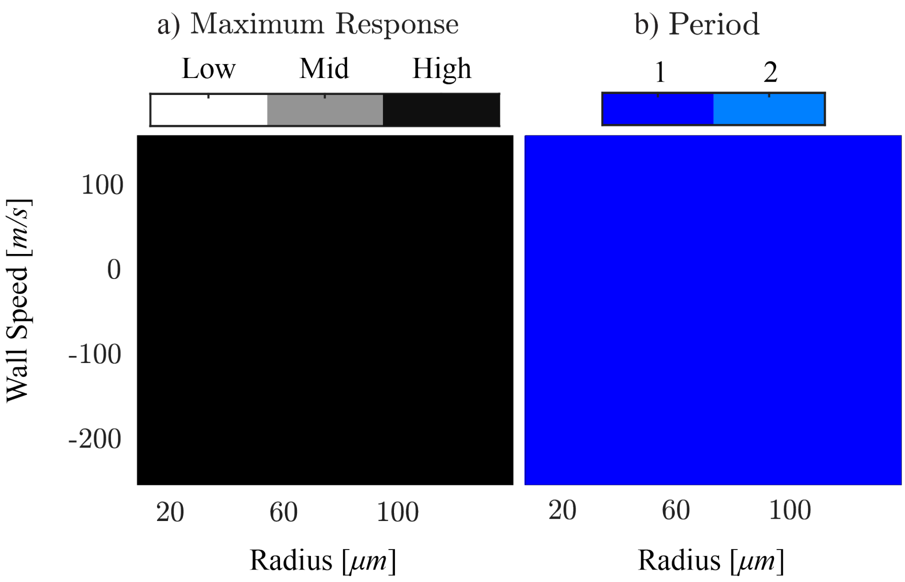

Figure S4.90: (a) the amplitude basins for bubble 6, where low-, mid- and high-amplitude attractor correspond to white, gray and black; and (b) the corresponding period basins of these attractors, as indicated by the colorbar.

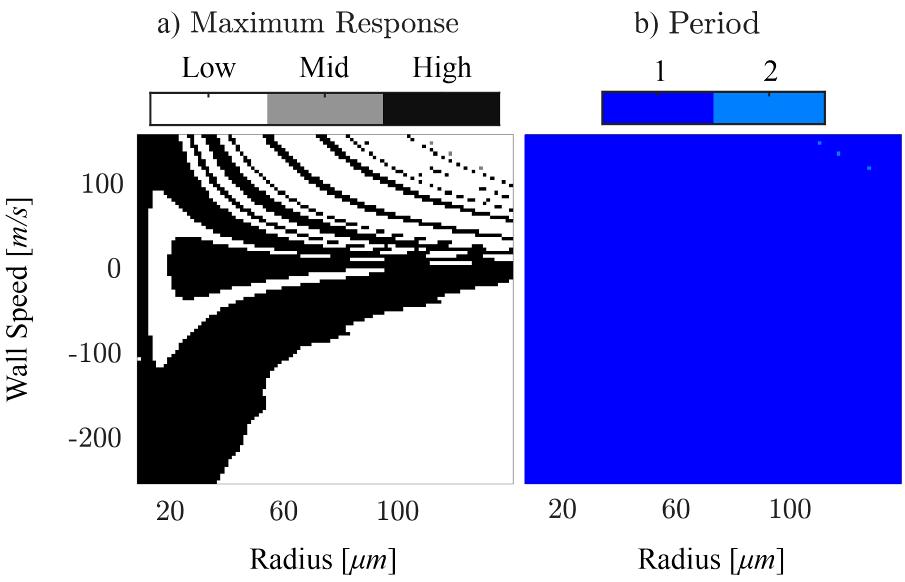

Figure S4.91: (a) the amplitude basins for bubble 7, where low-, mid- and high-amplitude attractor correspond to white, gray and black; and (b) the corresponding period basins of these attractors, as indicated by the colorbar.

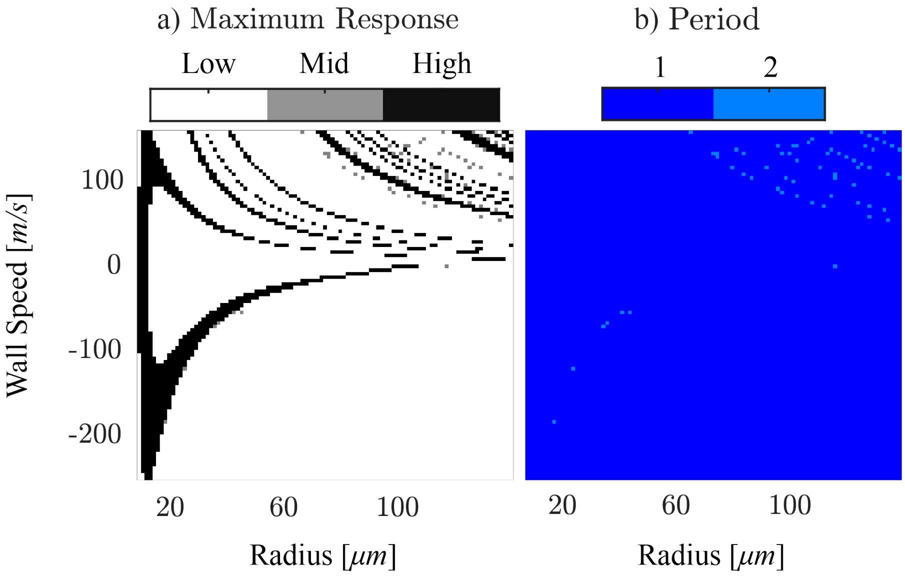

Figure S4.92: (a) the amplitude basins for bubble 8, where low-, mid- and high-amplitude attractor correspond to white, gray and black; and (b) the corresponding period basins of these attractors, as indicated by the colorbar.

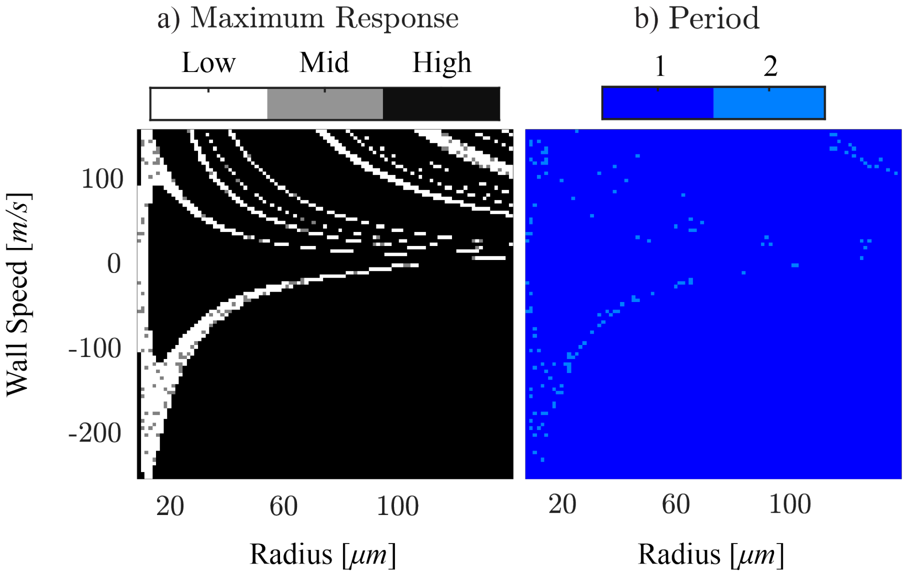

Figure S4.93: (a) the amplitude basins for bubble 9, where low-, mid- and high-amplitude attractor correspond to white, gray and black; and (b) the corresponding period basins of these attractors, as indicated by the colorbar.

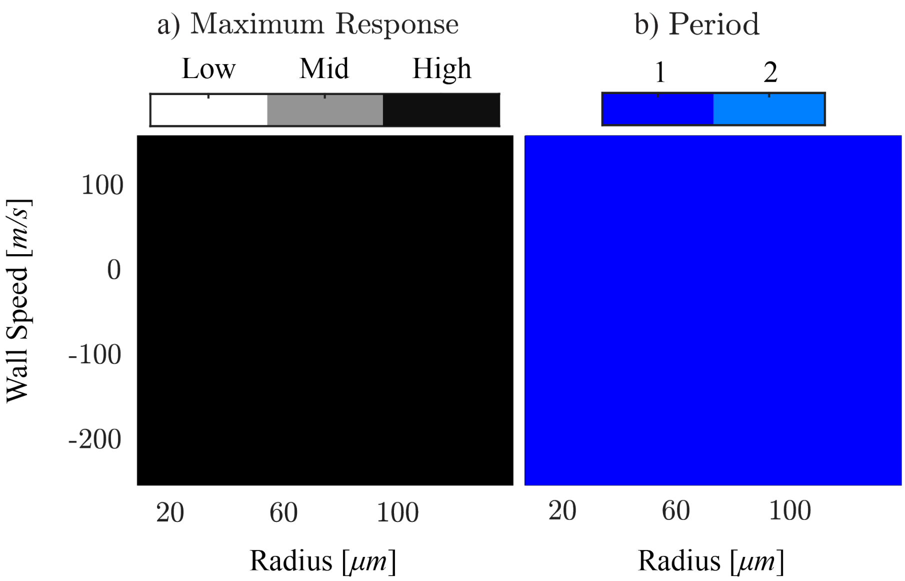

Figure S4.94: (a) the amplitude basins for bubble 10, where low-, mid- and high-amplitude attractor correspond to white, gray and black; and (b) the corresponding period basins of these attractors, as indicated by the colorbar.

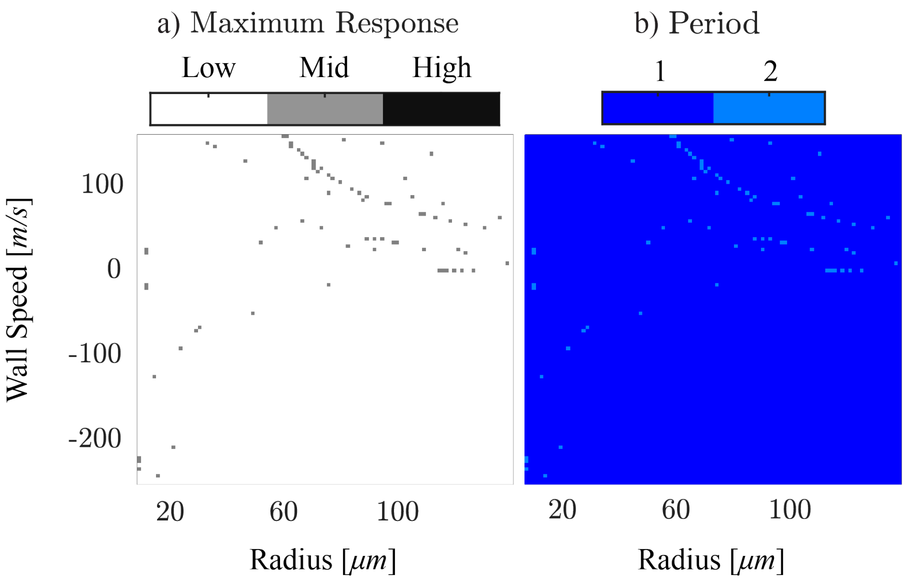

Figure S4.95: (a) the amplitude basins for bubble 11, where low-, mid- and high-amplitude attractor correspond to white, gray and black; and (b) the corresponding period basins of these attractors, as indicated by the colorbar.

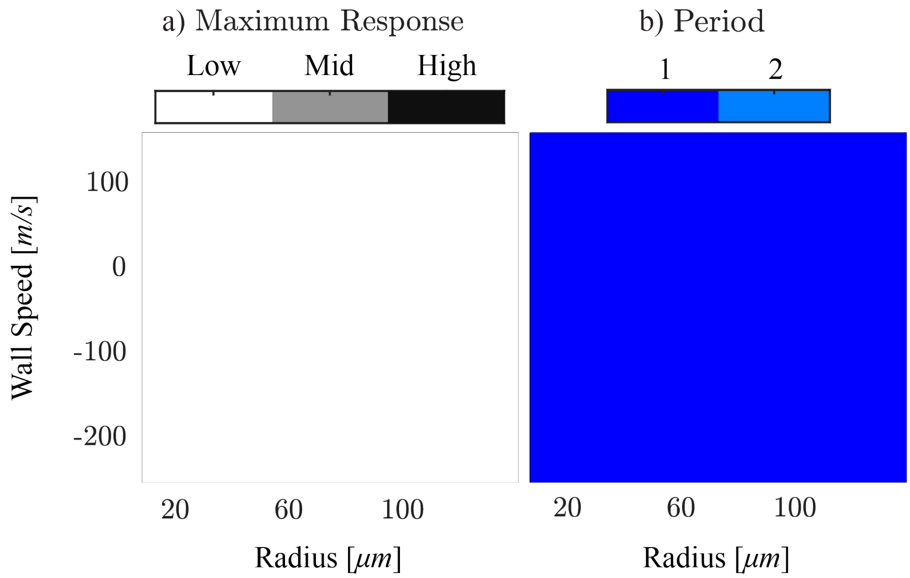

Figure S4.96: (a) the amplitude basins for bubble 12, where low-, mid- and high-amplitude attractor correspond to white, gray and black; and (b) the corresponding period basins of these attractors, as indicated by the colorbar.

Control bubble: Bubble 9

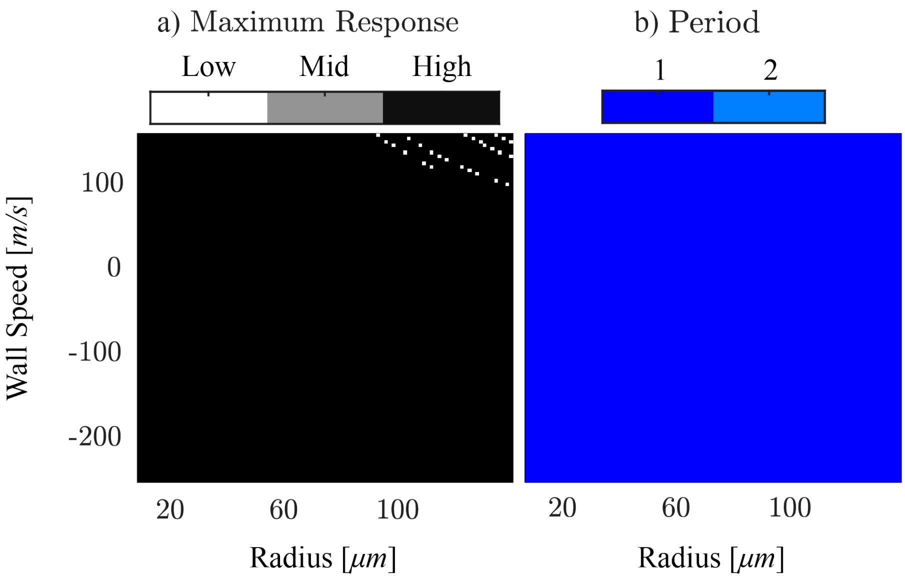

Figure S4.97: (a) the amplitude basins for bubble 1, where low-, mid- and high-amplitude attractor correspond to white, gray and black; and (b) the corresponding period basins of these attractors, as indicated by the colorbar.

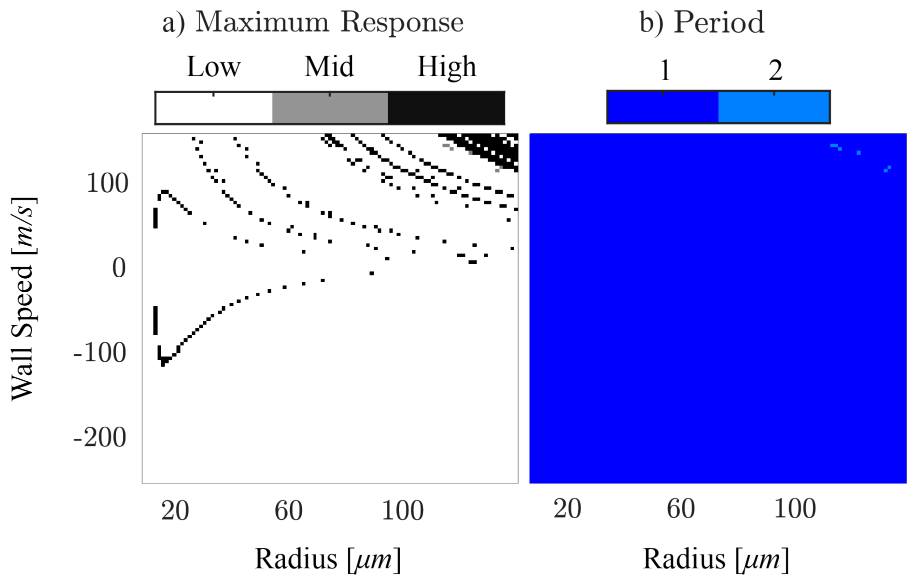

Figure S4.98: (a) the amplitude basins for bubble 2, where low-, mid- and high-amplitude attractor correspond to white, gray and black; and (b) the corresponding period basins of these attractors, as indicated by the colorbar.

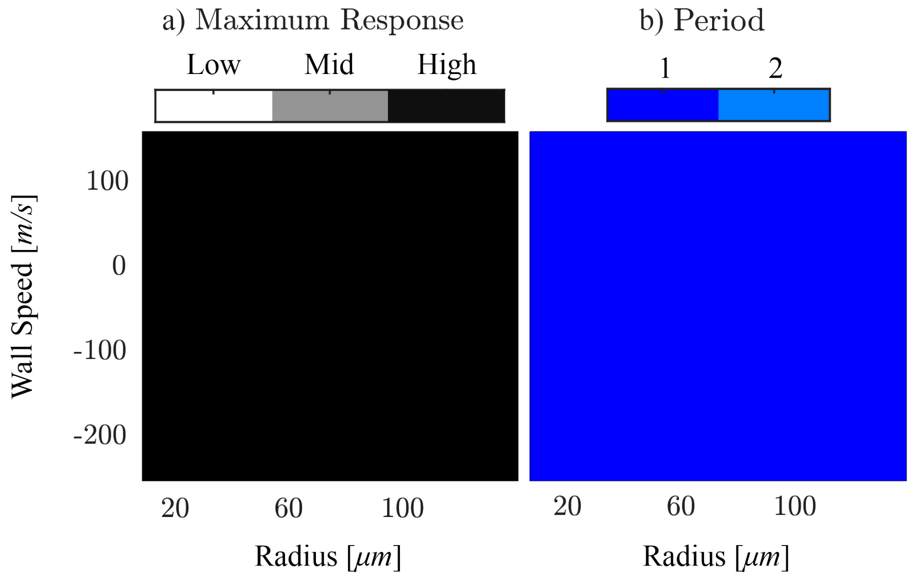

Figure S4.99: (a) the amplitude basins for bubble 3, where low-, mid- and high-amplitude attractor correspond to white, gray and black; and (b) the corresponding period basins of these attractors, as indicated by the colorbar.

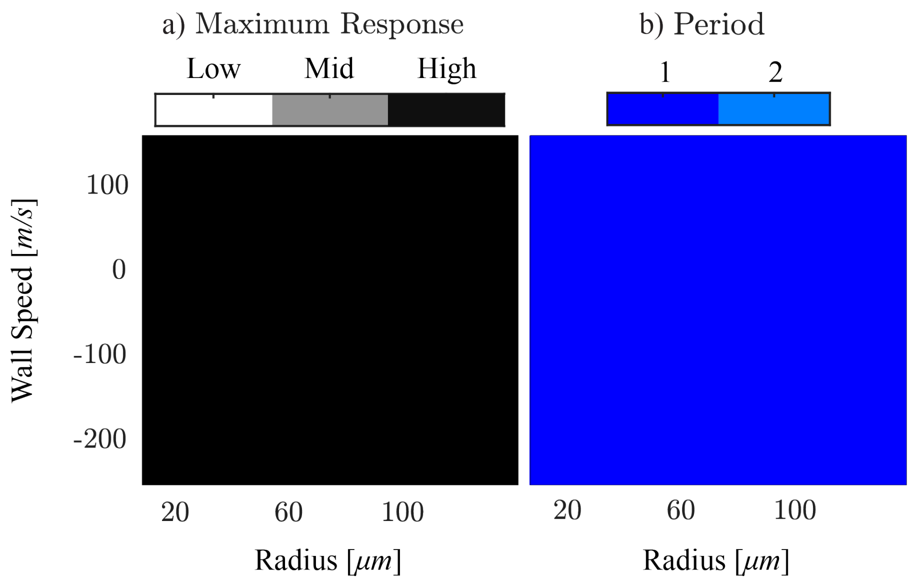

Figure S4.100: (a) the amplitude basins for bubble 4, where low-, mid- and high-amplitude attractor correspond to white, gray and black; and (b) the corresponding period basins of these attractors, as indicated by the colorbar.

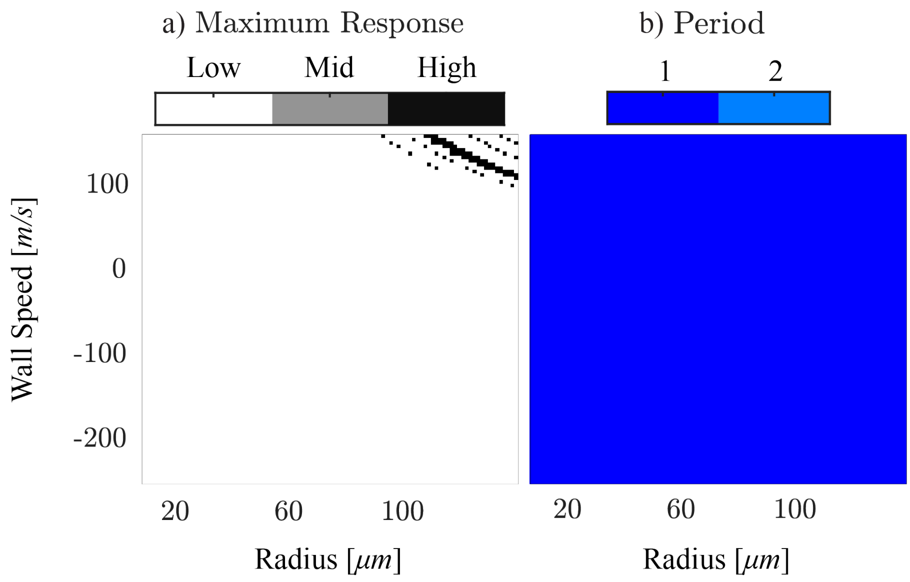

Figure S4.101: (a) the amplitude basins for bubble 5, where low-, mid- and high-amplitude attractor correspond to white, gray and black; and (b) the corresponding period basins of these attractors, as indicated by the colorbar.

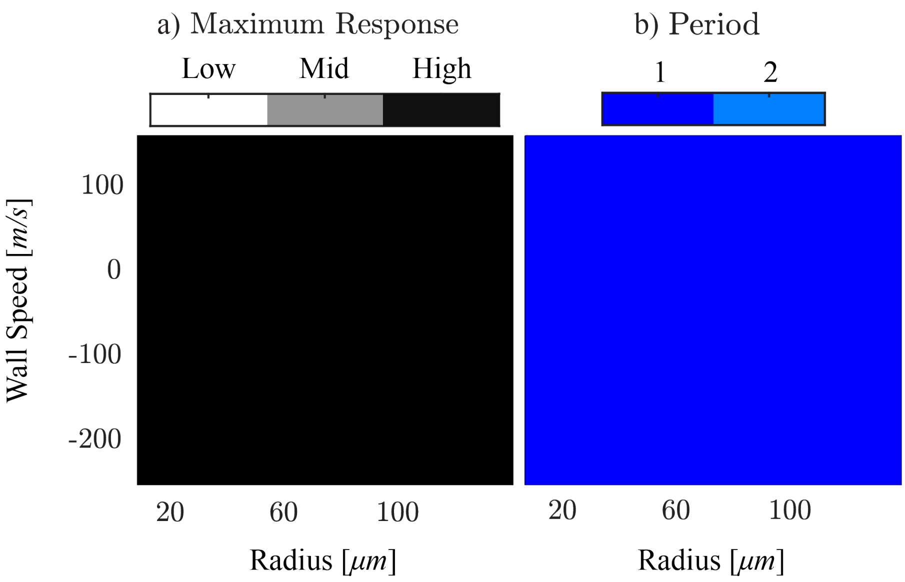

Figure S4.102: (a) the amplitude basins for bubble 6, where low-, mid- and high-amplitude attractor correspond to white, gray and black; and (b) the corresponding period basins of these attractors, as indicated by the colorbar.

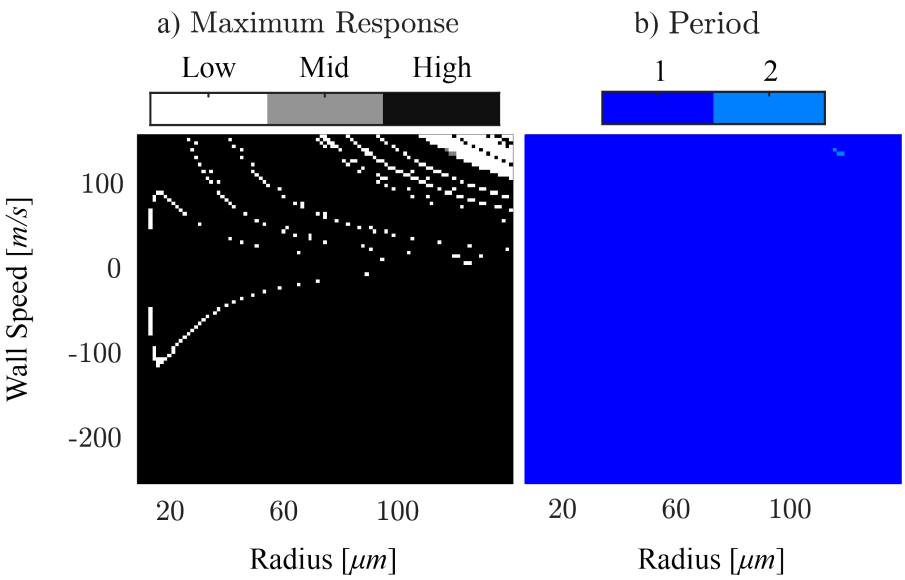

Figure S4.103: (a) the amplitude basins for bubble 7, where low-, mid- and high-amplitude attractor correspond to white, gray and black; and (b) the corresponding period basins of these attractors, as indicated by the colorbar.

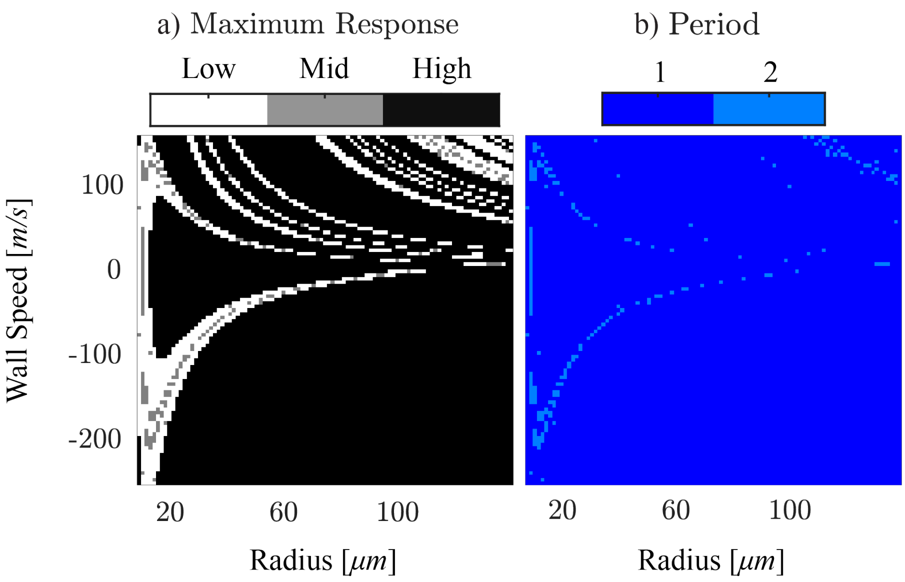

Figure S4.104: (a) the amplitude basins for bubble 8, where low-, mid- and high-amplitude attractor correspond to white, gray and black; and (b) the corresponding period basins of these attractors, as indicated by the colorbar.

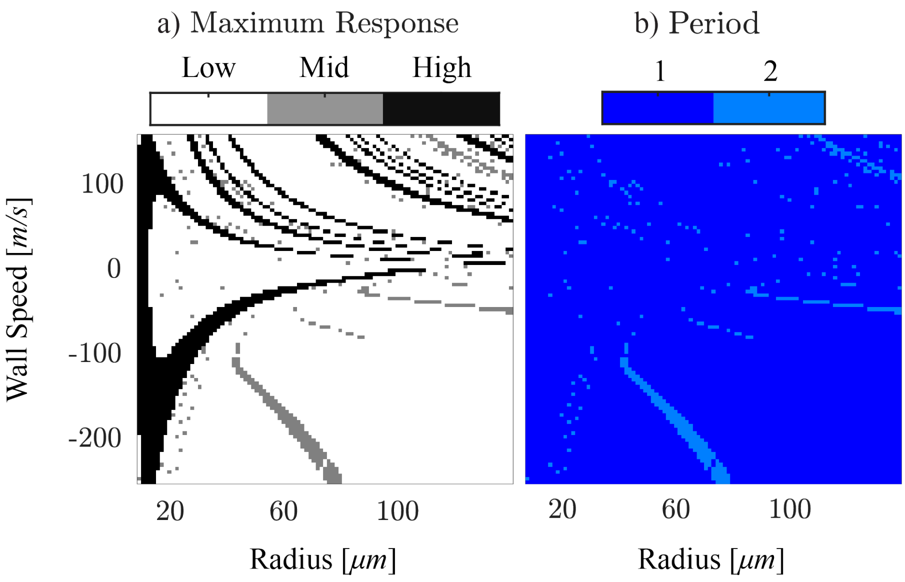

Figure S4.105: (a) the amplitude basins for bubble 9, where low-, mid- and high-amplitude attractor correspond to white, gray and black; and (b) the corresponding period basins of these attractors, as indicated by the colorbar.

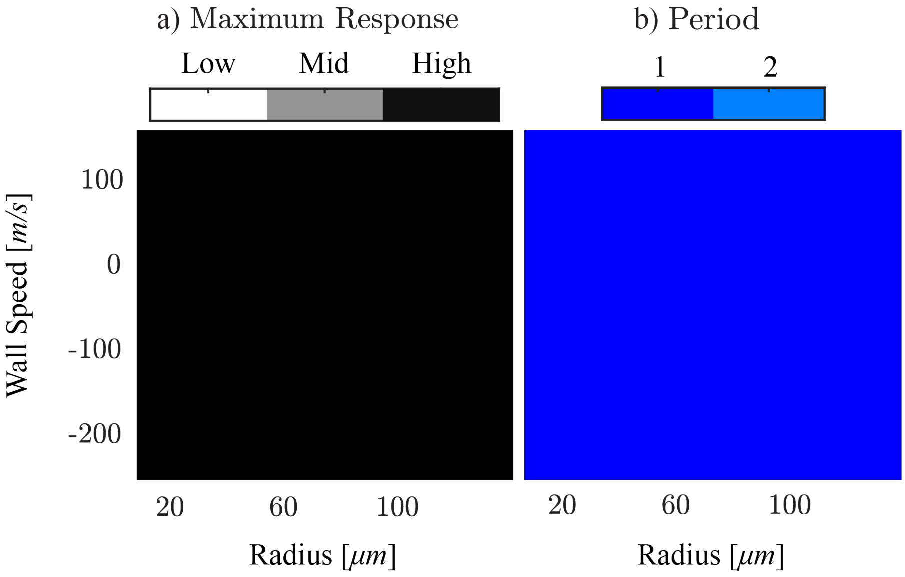

Figure S4.106: (a) the amplitude basins for bubble 10, where low-, mid- and high-amplitude attractor correspond to white, gray and black; and (b) the corresponding period basins of these attractors, as indicated by the colorbar.

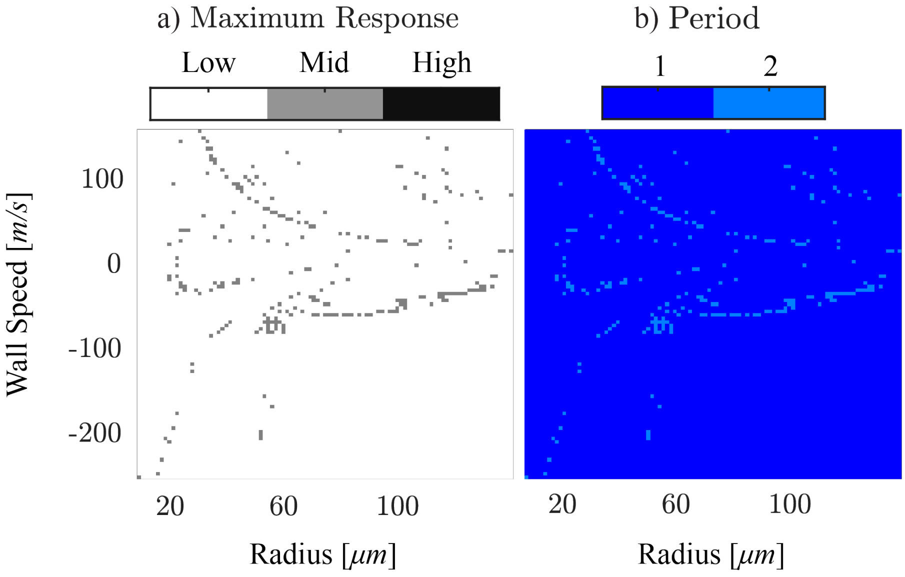

Figure S4.107: (a) the amplitude basins for bubble 11, where low-, mid- and high-amplitude attractor correspond to white, gray and black; and (b) the corresponding period basins of these attractors, as indicated by the colorbar.

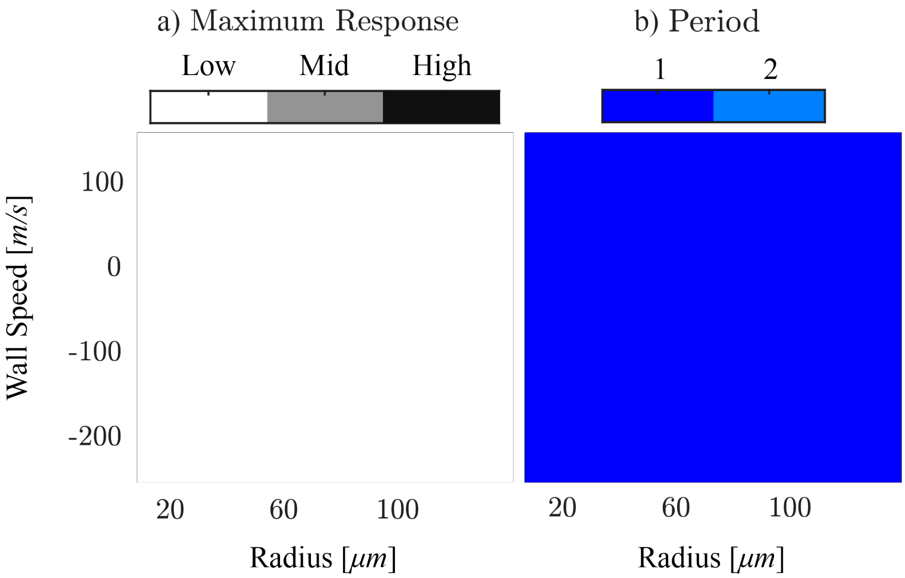

Figure S4.108: (a) the amplitude basins for bubble 12, where low-, mid- and high-amplitude attractor correspond to white, gray and black; and (b) the corresponding period basins of these attractors, as indicated by the colorbar.

Control bubble: Bubble 10

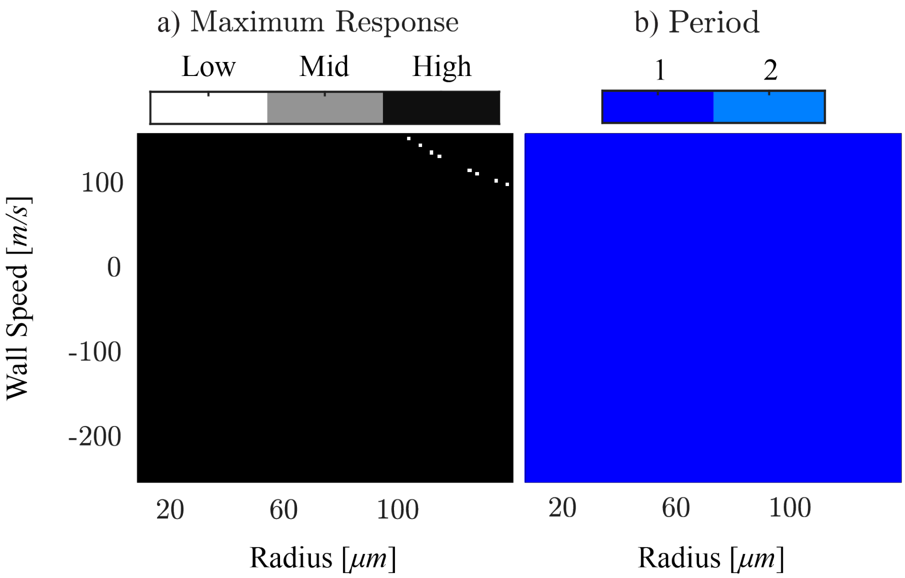

Figure S4.109: (a) the amplitude basins for bubble 1, where low-, mid- and high-amplitude attractor correspond to white, gray and black; and (b) the corresponding period basins of these attractors, as indicated by the colorbar.

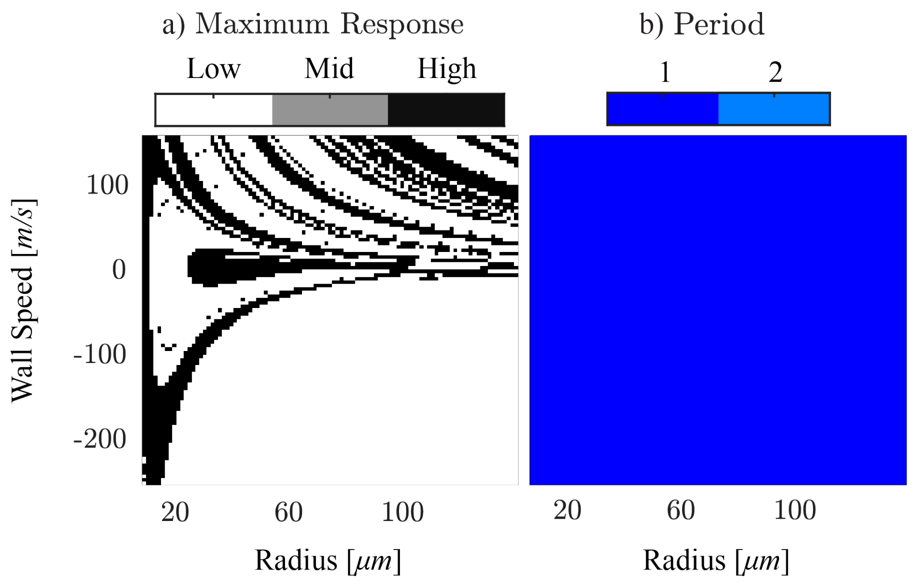

Figure S4.110: (a) the amplitude basins for bubble 2, where low-, mid- and high-amplitude attractor correspond to white, gray and black; and (b) the corresponding period basins of these attractors, as indicated by the colorbar.

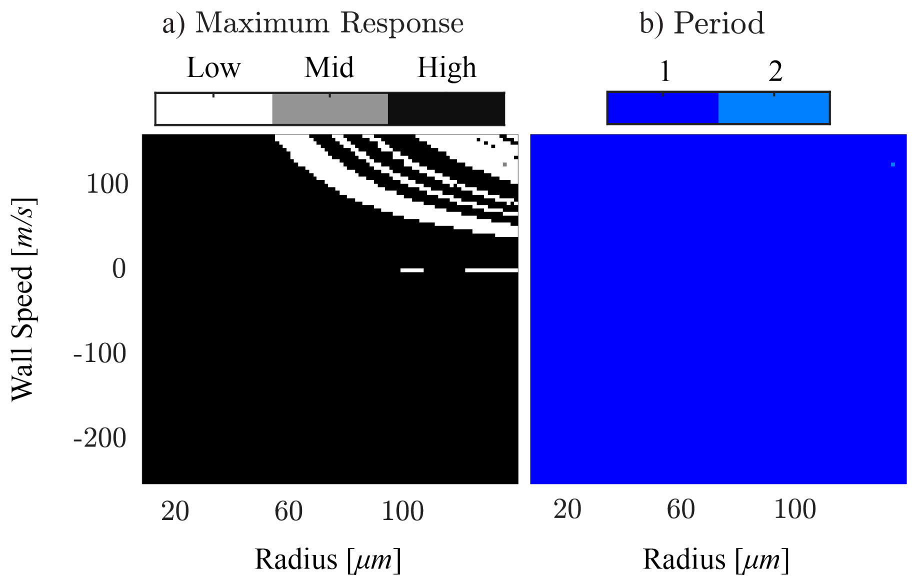

Figure S4.111: (a) the amplitude basins for bubble 3, where low-, mid- and high-amplitude attractor correspond to white, gray and black; and (b) the corresponding period basins of these attractors, as indicated by the colorbar.

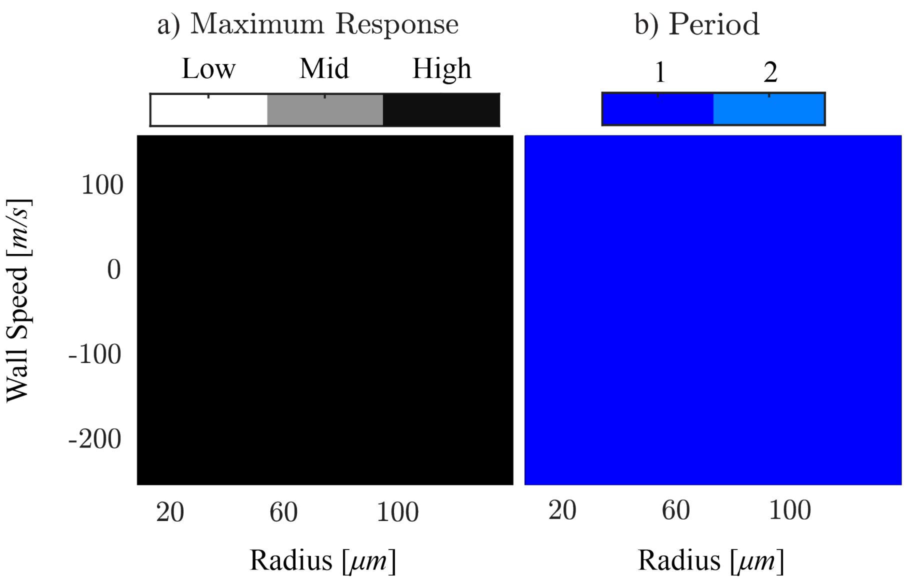

Figure S4.112: (a) the amplitude basins for bubble 4, where low-, mid- and high-amplitude attractor correspond to white, gray and black; and (b) the corresponding period basins of these attractors, as indicated by the colorbar.

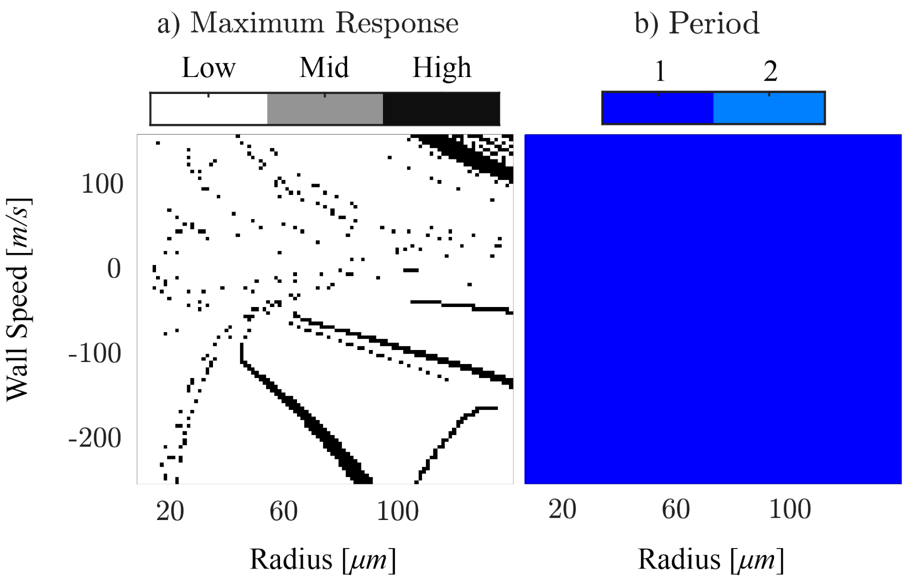

Figure S4.113: (a) the amplitude basins for bubble 5, where low-, mid- and high-amplitude attractor correspond to white, gray and black; and (b) the corresponding period basins of these attractors, as indicated by the colorbar.

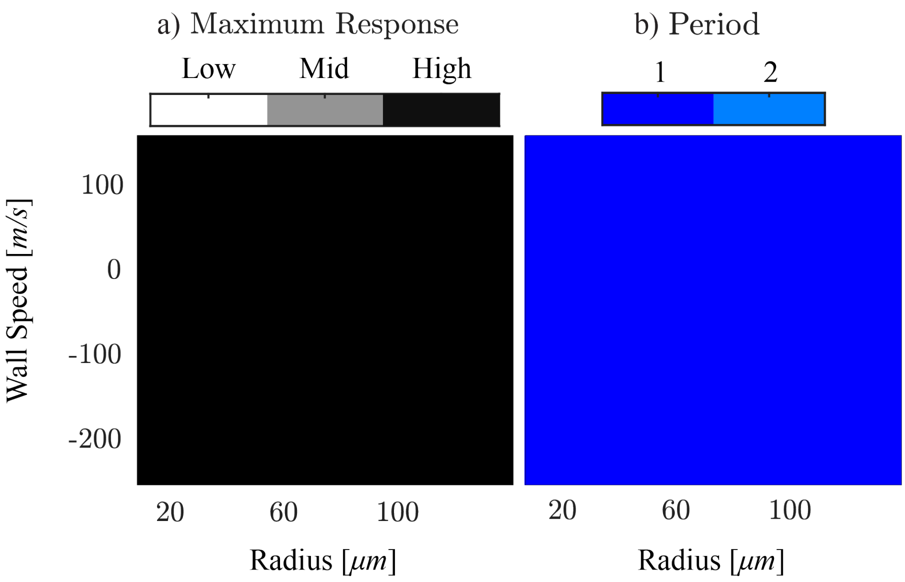

Figure S4.114: (a) the amplitude basins for bubble 6, where low-, mid- and high-amplitude attractor correspond to white, gray and black; and (b) the corresponding period basins of these attractors, as indicated by the colorbar.

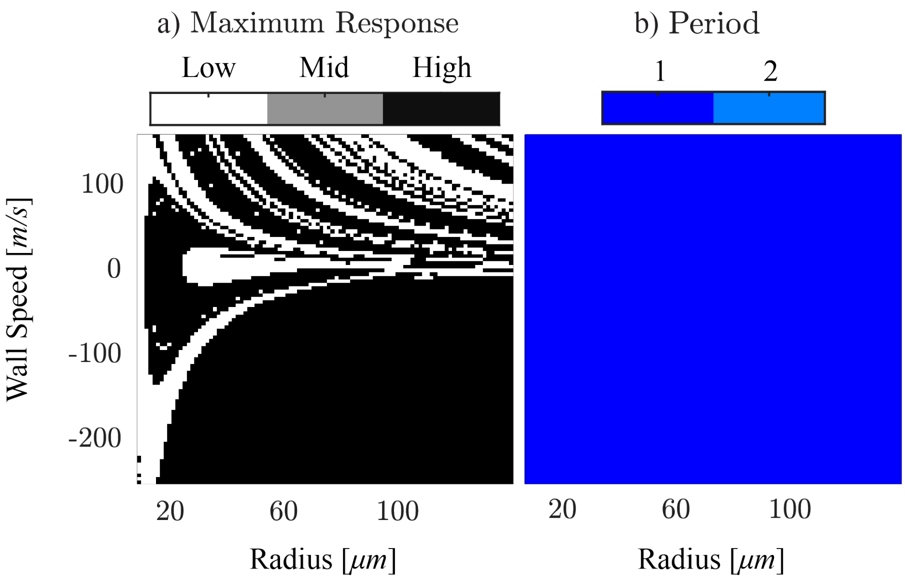

Figure S4.115: (a) the amplitude basins for bubble 7, where low-, mid- and high-amplitude attractor correspond to white, gray and black; and (b) the corresponding period basins of these attractors, as indicated by the colorbar.

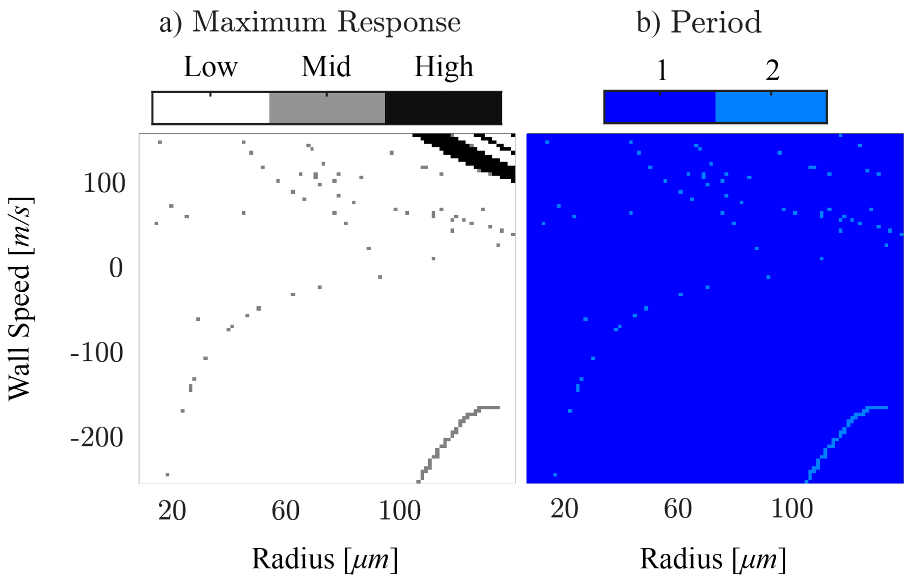

Figure S4.116: (a) the amplitude basins for bubble 8, where low-, mid- and high-amplitude attractor correspond to white, gray and black; and (b) the corresponding period basins of these attractors, as indicated by the colorbar.

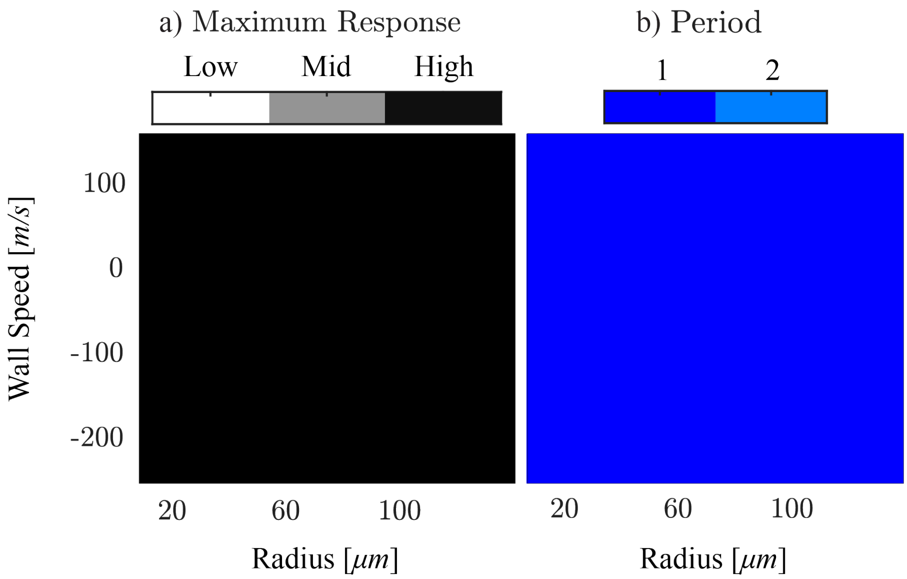

Figure S4.117: (a) the amplitude basins for bubble 9, where low-, mid- and high-amplitude attractor correspond to white, gray and black; and (b) the corresponding period basins of these attractors, as indicated by the colorbar.

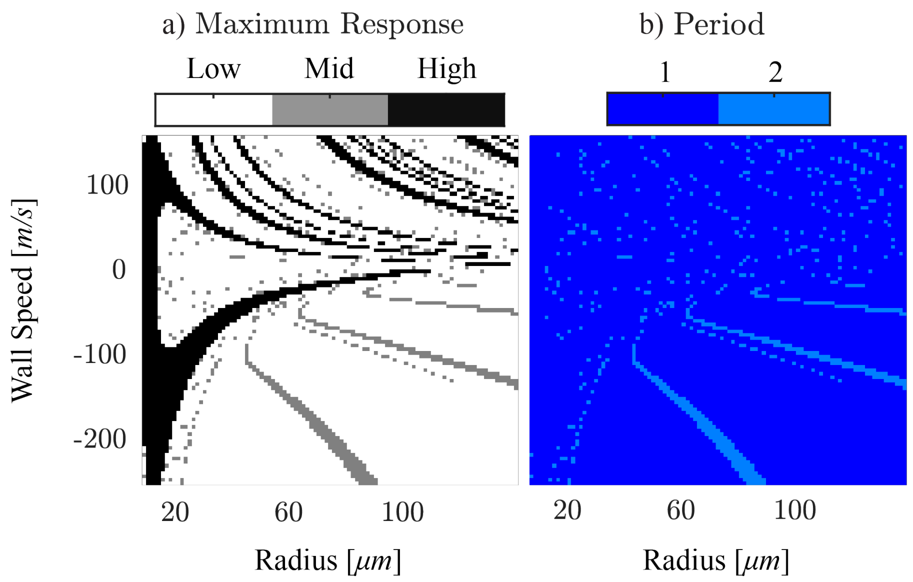

Figure S4.118: (a) the amplitude basins for bubble 10, where low-, mid- and high-amplitude attractor correspond to white, gray and black; and (b) the corresponding period basins of these attractors, as indicated by the colorbar.

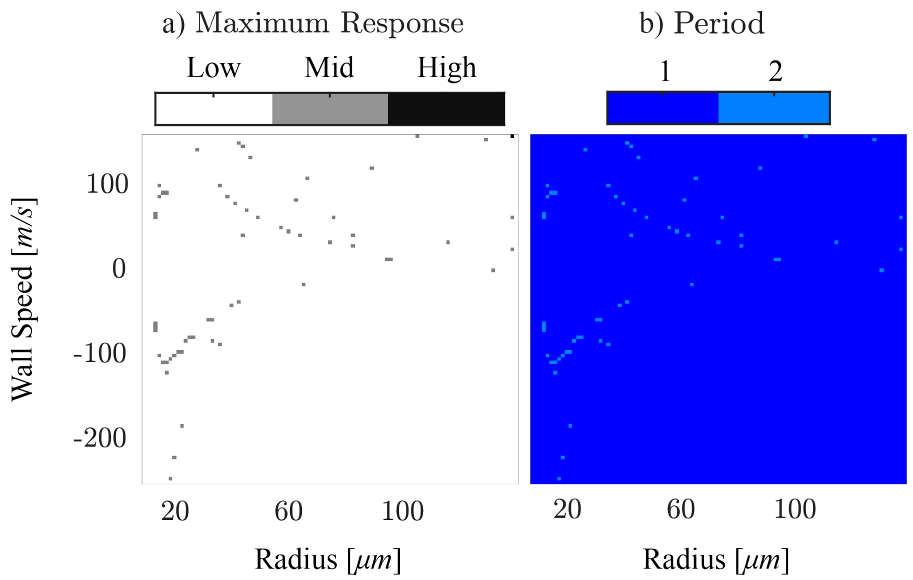

Figure S4.119: (a) the amplitude basins for bubble 11, where low-, mid- and high-amplitude attractor correspond to white, gray and black; and (b) the corresponding period basins of these attractors, as indicated by the colorbar.

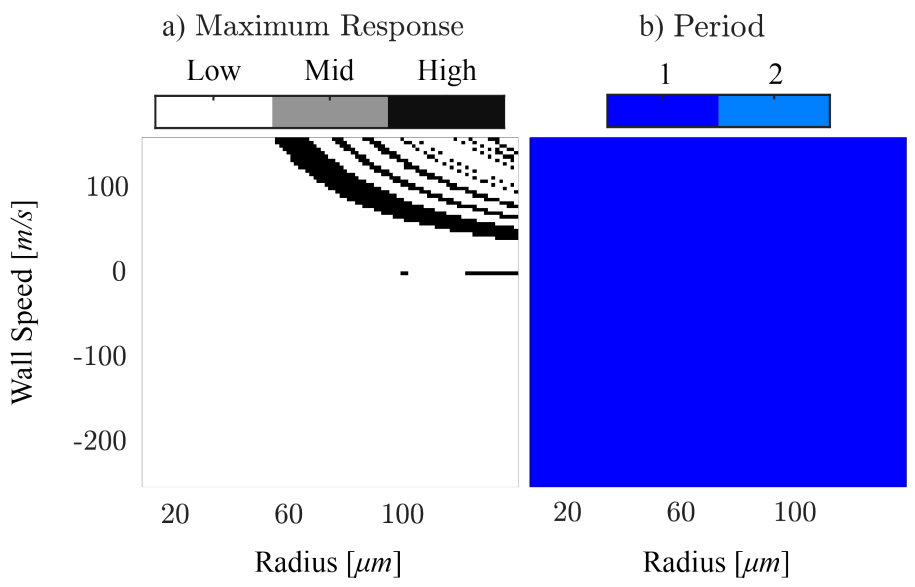

Figure S4.120: (a) the amplitude basins for bubble 12, where low-, mid- and high-amplitude attractor correspond to white, gray and black; and (b) the corresponding period basins of these attractors, as indicated by the colorbar.

Control bubble: Bubble 11

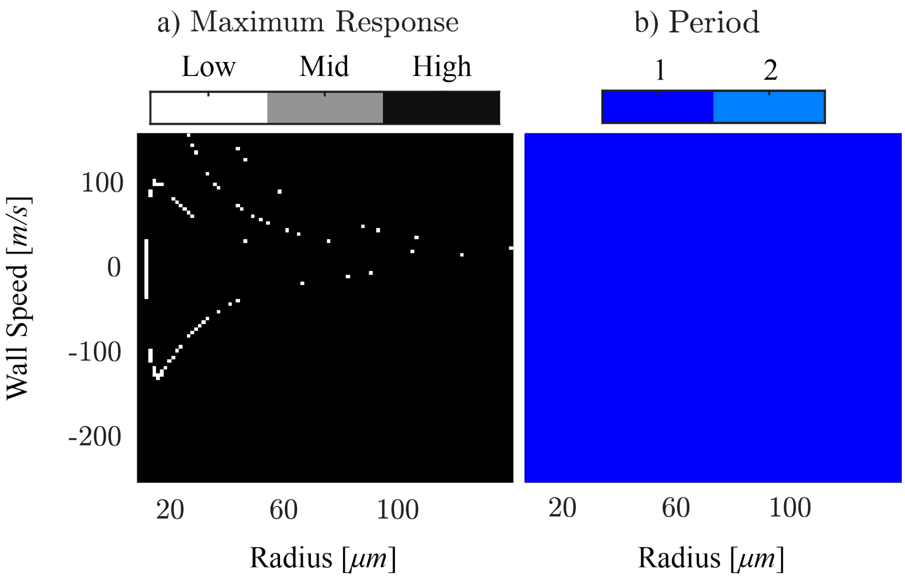

Figure S4.121: (a) the amplitude basins for bubble 1, where low-, mid- and high-amplitude attractor correspond to white, gray and black; and (b) the corresponding period basins of these attractors, as indicated by the colorbar.

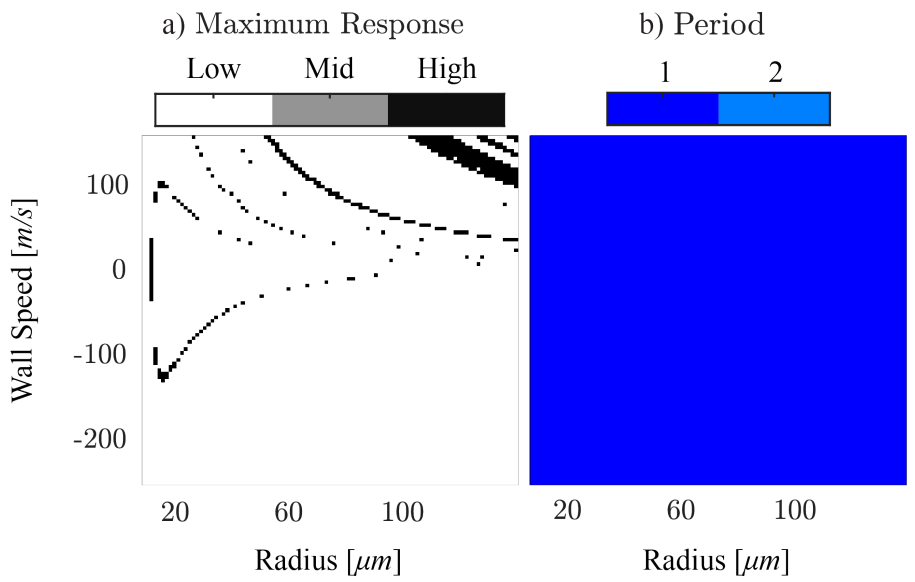

Figure S4.122: (a) the amplitude basins for bubble 2, where low-, mid- and high-amplitude attractor correspond to white, gray and black; and (b) the corresponding period basins of these attractors, as indicated by the colorbar.

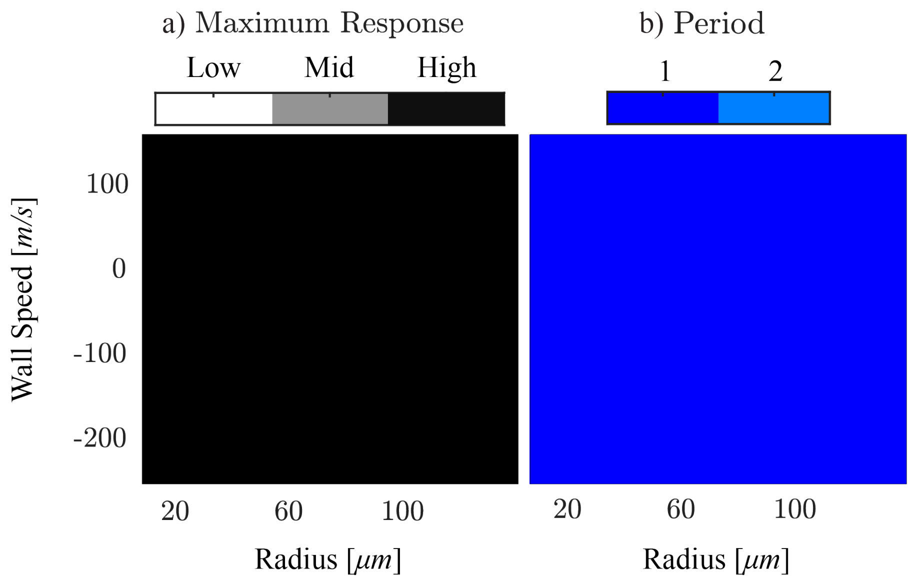

Figure S4.123: (a) the amplitude basins for bubble 3, where low-, mid- and high-amplitude attractor correspond to white, gray and black; and (b) the corresponding period basins of these attractors, as indicated by the colorbar.

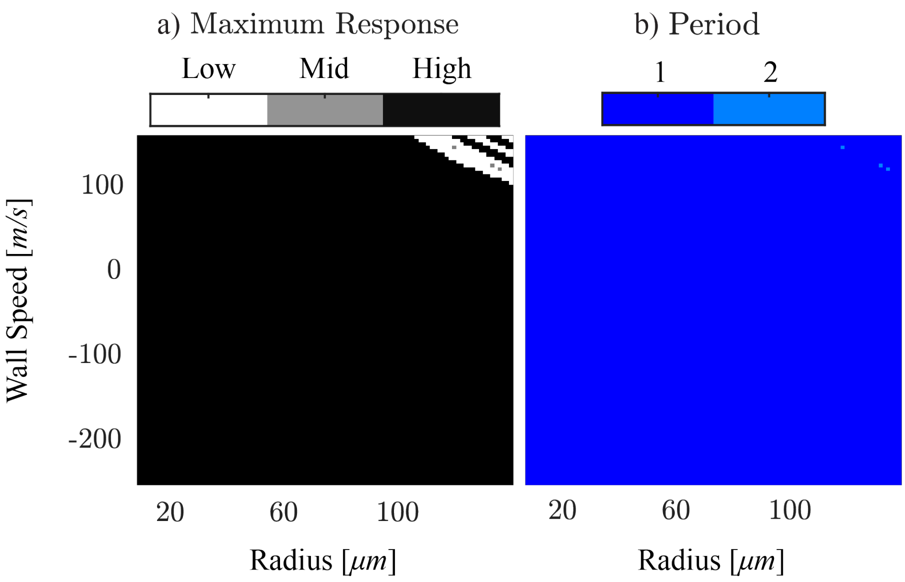

Figure S4.124: (a) the amplitude basins for bubble 4, where low-, mid- and high-amplitude attractor correspond to white, gray and black; and (b) the corresponding period basins of these attractors, as indicated by the colorbar.

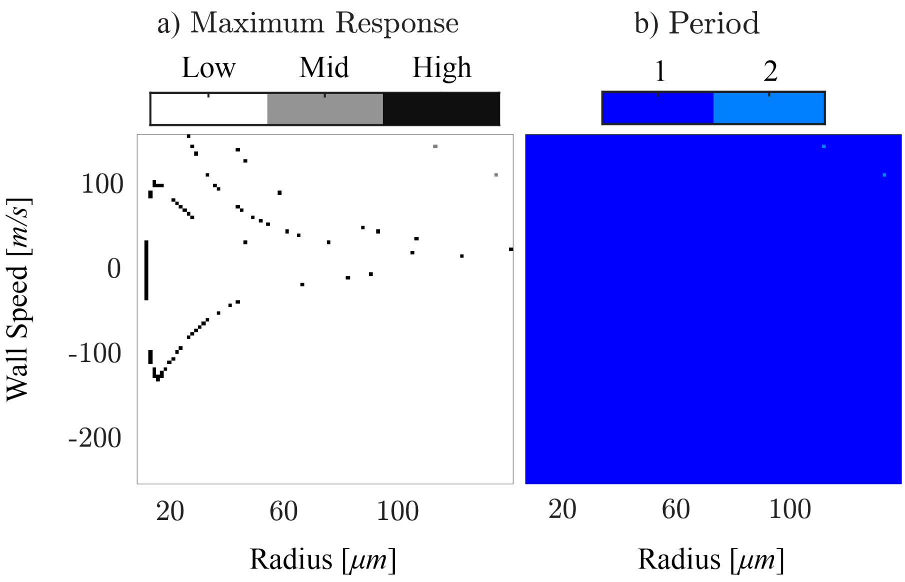

Figure S4.125: (a) the amplitude basins for bubble 5, where low-, mid- and high-amplitude attractor correspond to white, gray and black; and (b) the corresponding period basins of these attractors, as indicated by the colorbar.

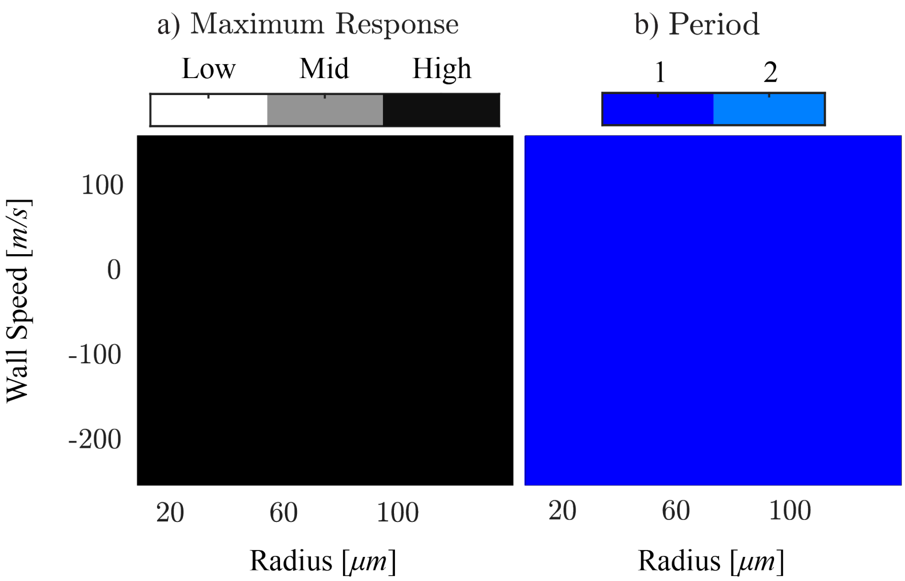

Figure S4.126: (a) the amplitude basins for bubble 6, where low-, mid- and high-amplitude attractor correspond to white, gray and black; and (b) the corresponding period basins of these attractors, as indicated by the colorbar.

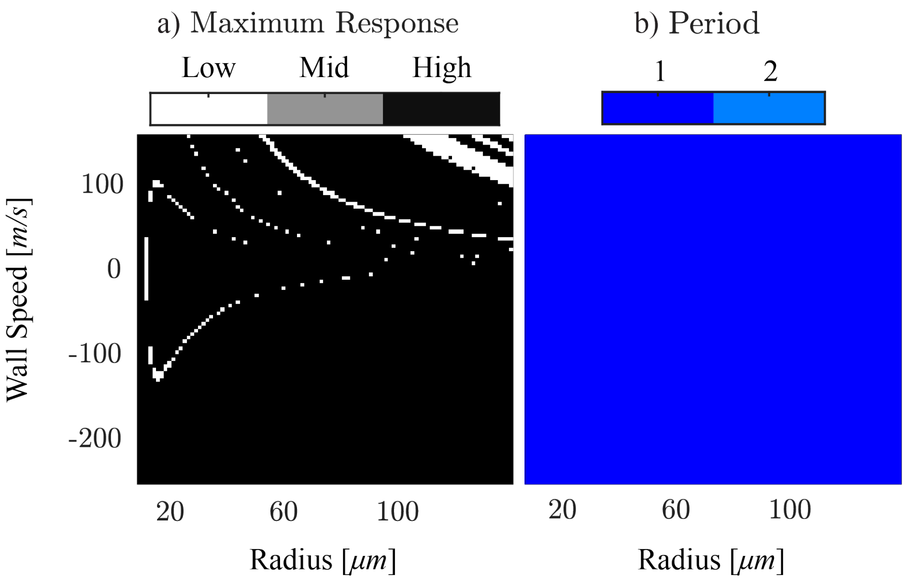

Figure S4.127: (a) the amplitude basins for bubble 7, where low-, mid- and high-amplitude attractor correspond to white, gray and black; and (b) the corresponding period basins of these attractors, as indicated by the colorbar.

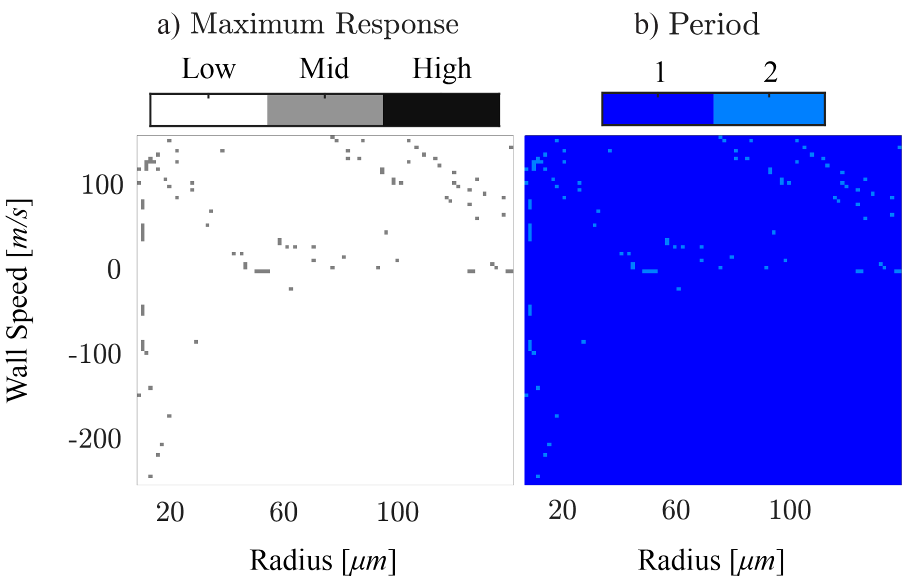

Figure S4.128: (a) the amplitude basins for bubble 8, where low-, mid- and high-amplitude attractor correspond to white, gray and black; and (b) the corresponding period basins of these attractors, as indicated by the colorbar.

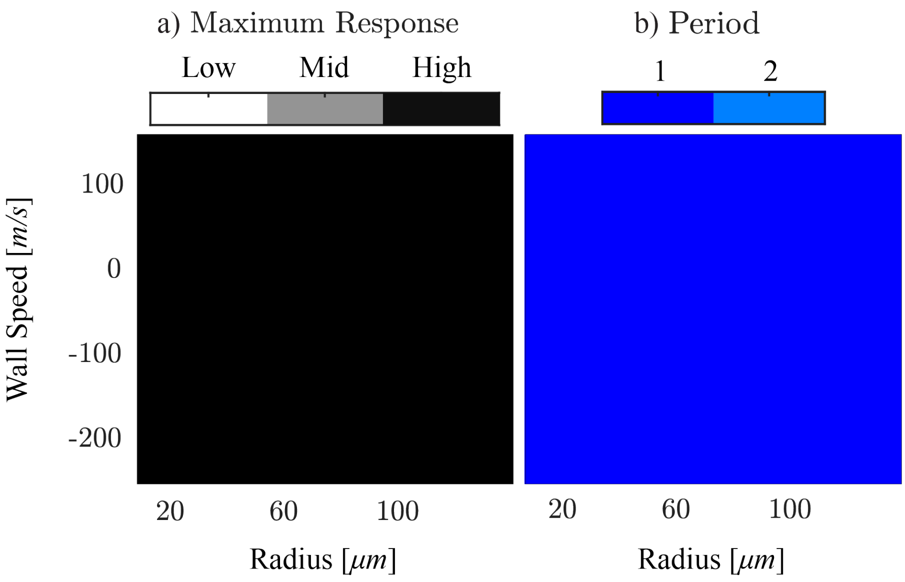

Figure S4.129: (a) the amplitude basins for bubble 9, where low-, mid- and high-amplitude attractor correspond to white, gray and black; and (b) the corresponding period basins of these attractors, as indicated by the colorbar.

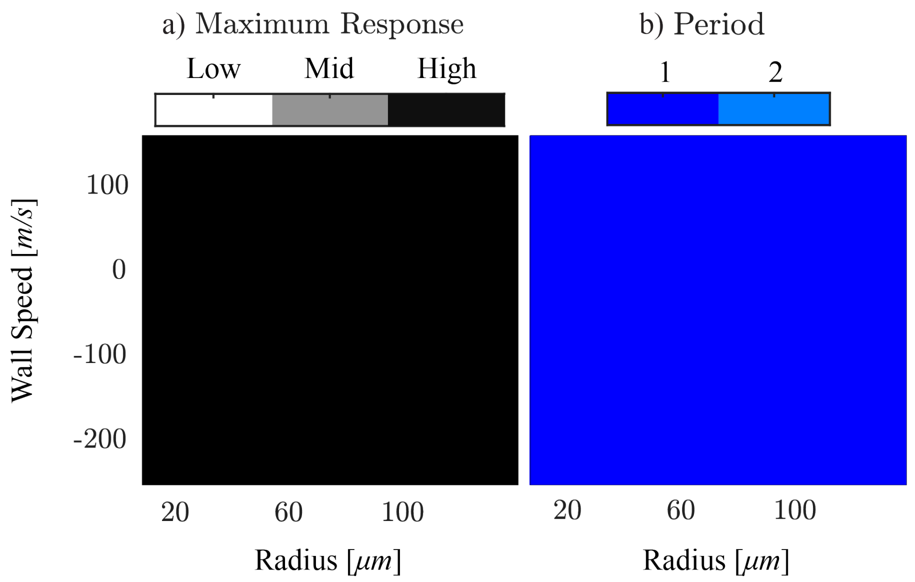

Figure S4.130: (a) the amplitude basins for bubble 10, where low-, mid- and high-amplitude attractor correspond to white, gray and black; and (b) the corresponding period basins of these attractors, as indicated by the colorbar.

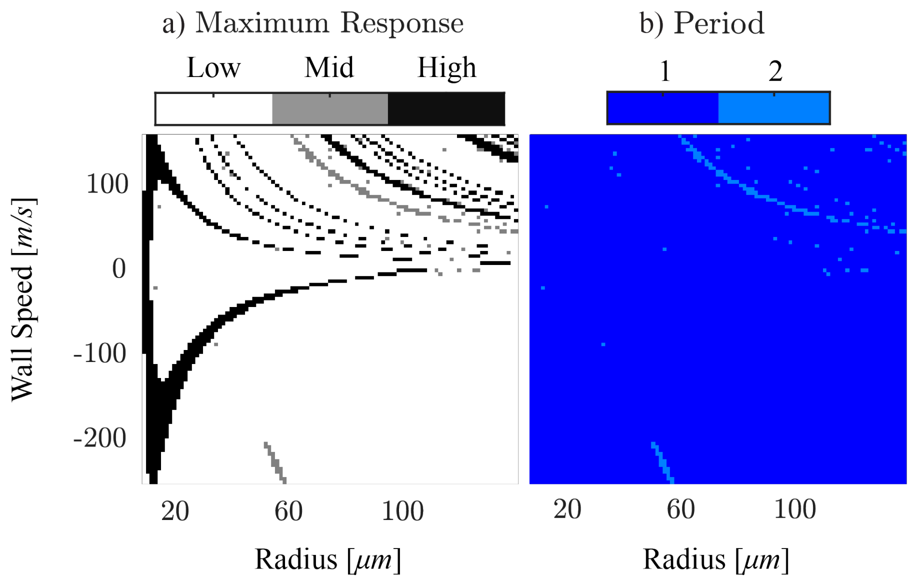

Figure S4.131: (a) the amplitude basins for bubble 11, where low-, mid- and high-amplitude attractor correspond to white, gray and black; and (b) the corresponding period basins of these attractors, as indicated by the colorbar.

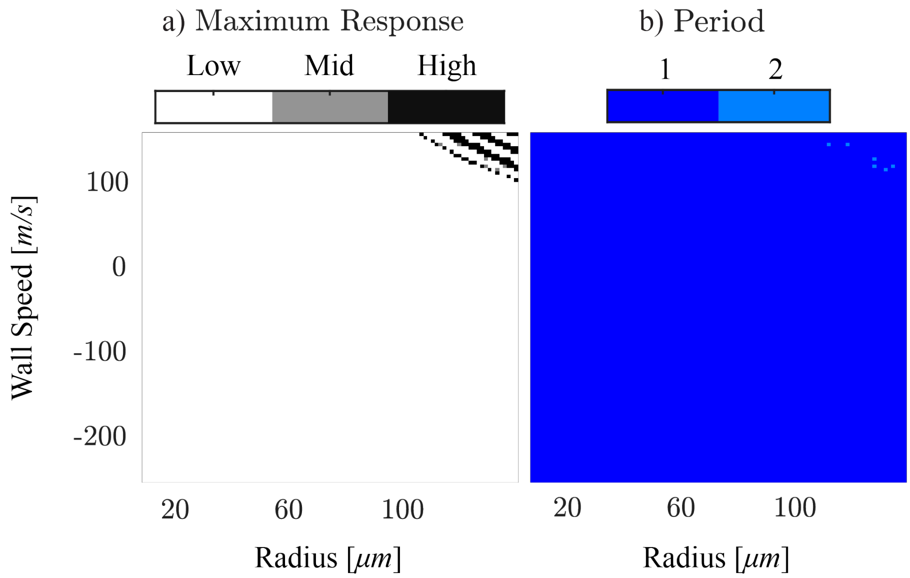

Figure S4.132: (a) the amplitude basins for bubble 12, where low-, mid- and high-amplitude attractor correspond to white, gray and black; and (b) the corresponding period basins of these attractors, as indicated by the colorbar.

Control bubble: Bubble 12

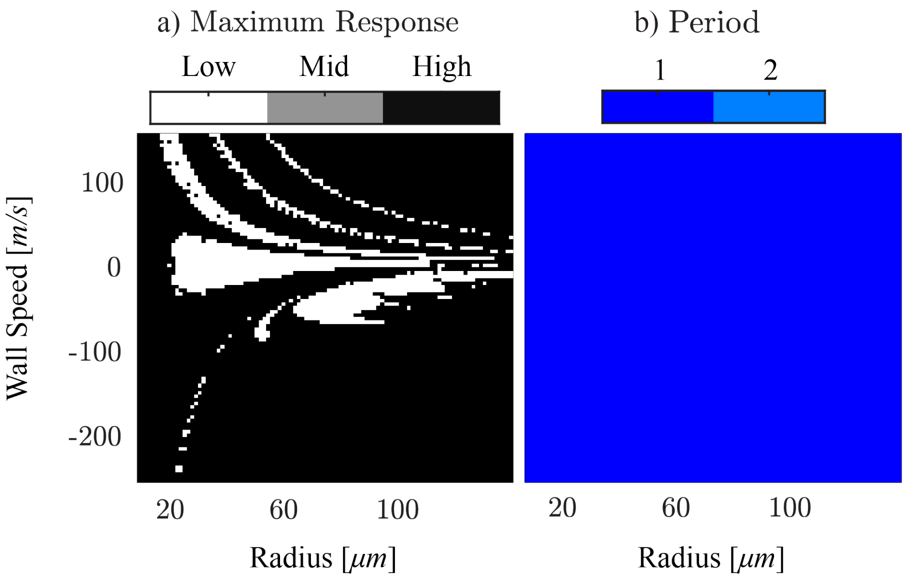

Figure S4.133: (a) the amplitude basins for bubble 1, where low-, mid- and high-amplitude attractor correspond to white, gray and black; and (b) the corresponding period basins of these attractors, as indicated by the colorbar.

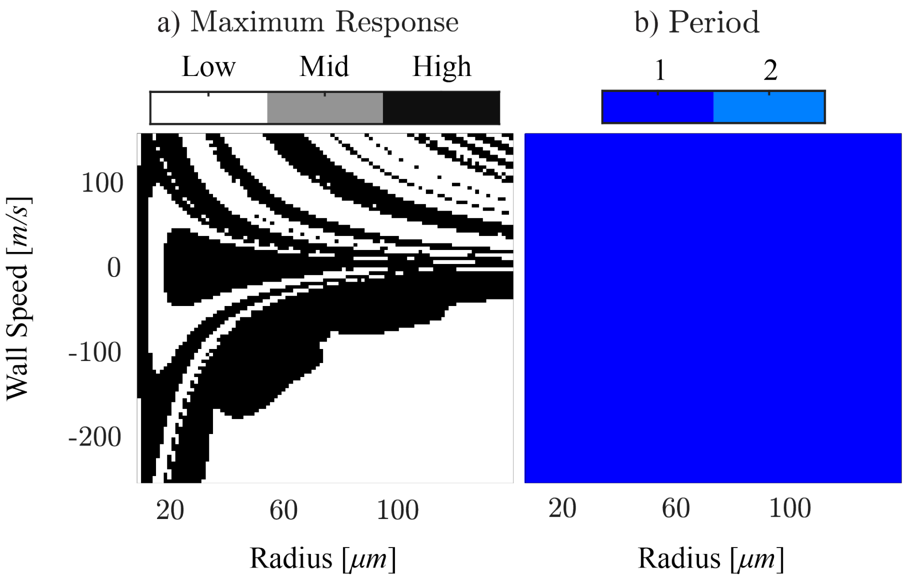

Figure S4.134: (a) the amplitude basins for bubble 2, where low-, mid- and high-amplitude attractor correspond to white, gray and black; and (b) the corresponding period basins of these attractors, as indicated by the colorbar.

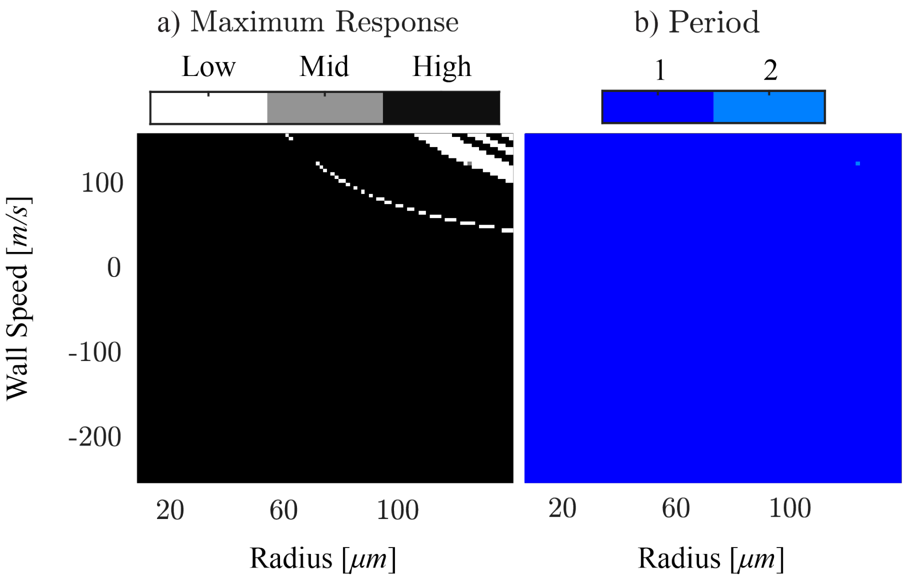

Figure S4.135: (a) the amplitude basins for bubble 3, where low-, mid- and high-amplitude attractor correspond to white, gray and black; and (b) the corresponding period basins of these attractors, as indicated by the colorbar.

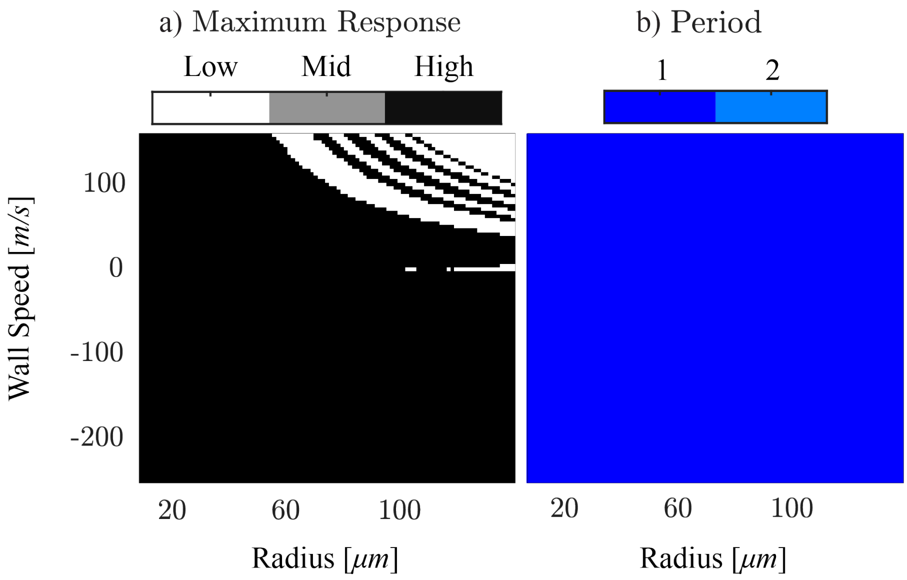

Figure S4.136: (a) the amplitude basins for bubble 4, where low-, mid- and high-amplitude attractor correspond to white, gray and black; and (b) the corresponding period basins of these attractors, as indicated by the colorbar.

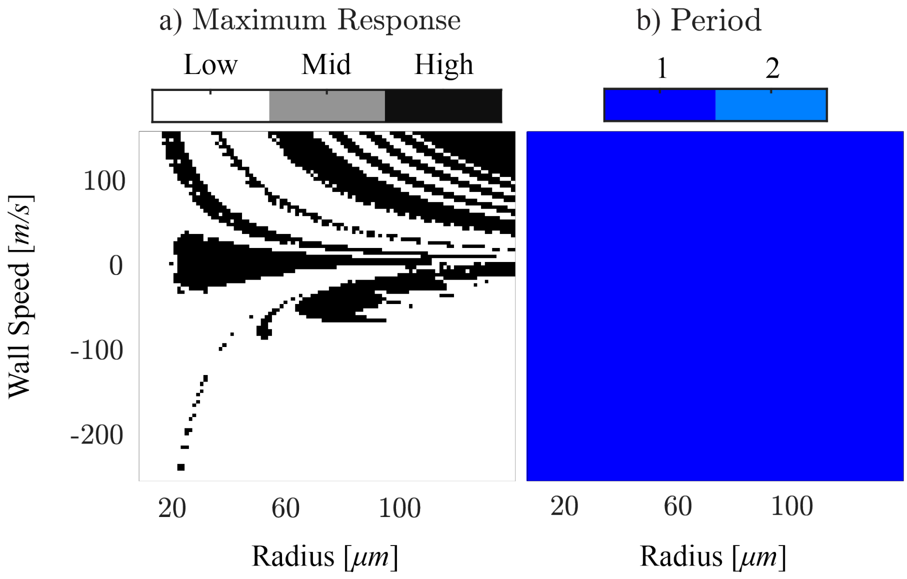

Figure S4.137: (a) the amplitude basins for bubble 5, where low-, mid- and high-amplitude attractor correspond to white, gray and black; and (b) the corresponding period basins of these attractors, as indicated by the colorbar.

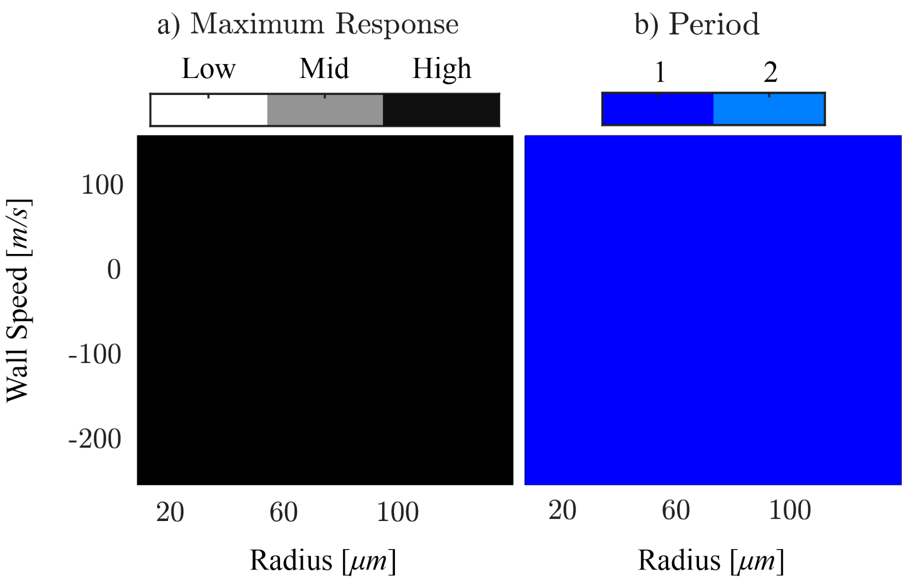

Figure S4.138: (a) the amplitude basins for bubble 6, where low-, mid- and high-amplitude attractor correspond to white, gray and black; and (b) the corresponding period basins of these attractors, as indicated by the colorbar.

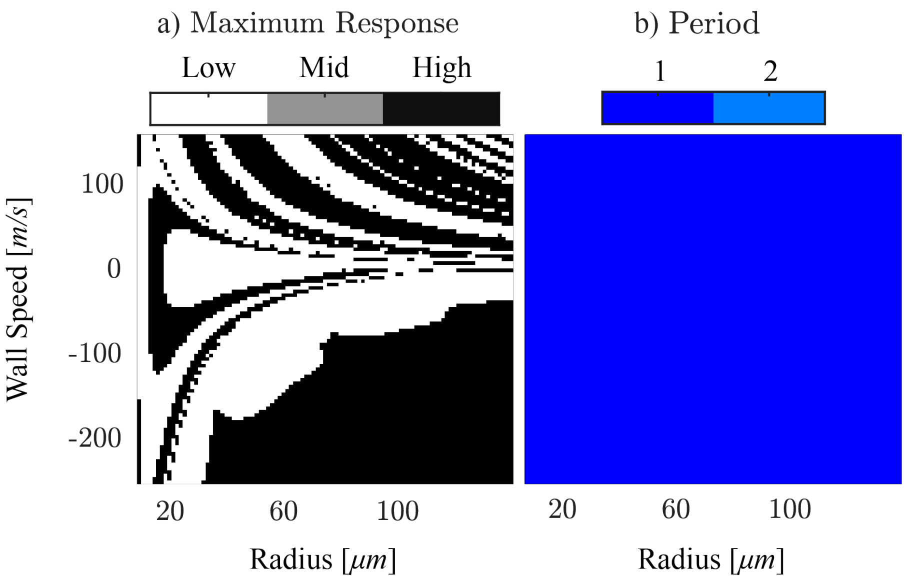

Figure S4.139: (a) the amplitude basins for bubble 7, where low-, mid- and high-amplitude attractor correspond to white, gray and black; and (b) the corresponding period basins of these attractors, as indicated by the colorbar.

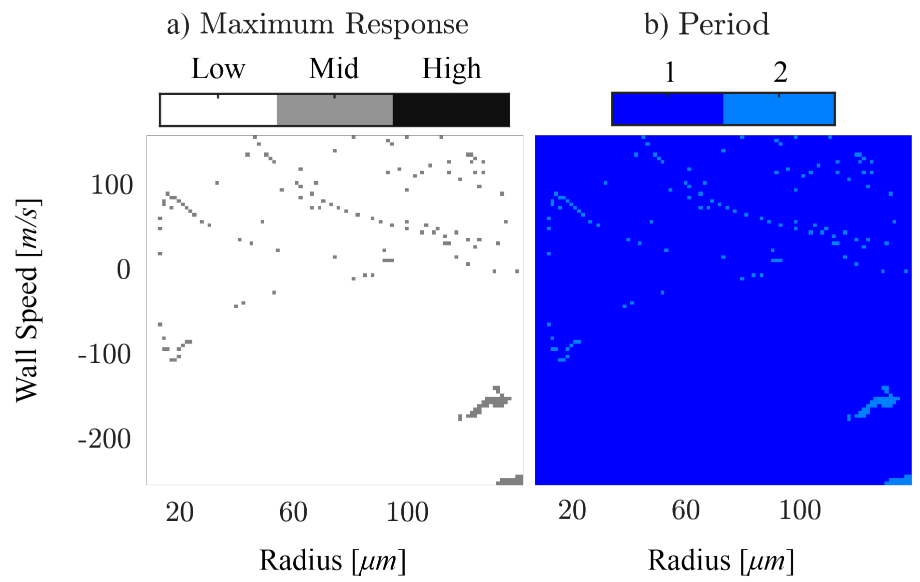

Figure S4.140: (a) the amplitude basins for bubble 8, where low-, mid- and high-amplitude attractor correspond to white, gray and black; and (b) the corresponding period basins of these attractors, as indicated by the colorbar.

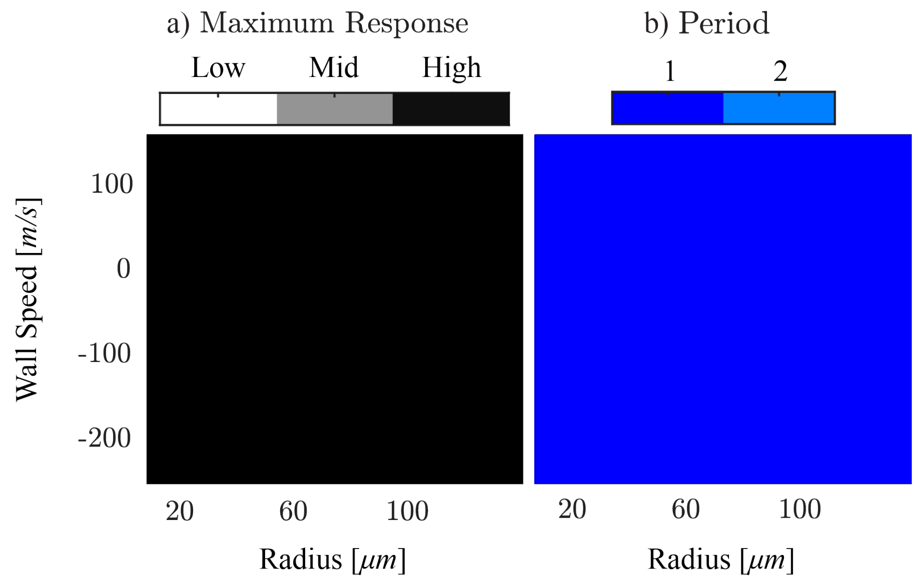

Figure S4.141: (a) the amplitude basins for bubble 9, where low-, mid- and high-amplitude attractor correspond to white, gray and black; and (b) the corresponding period basins of these attractors, as indicated by the colorbar.

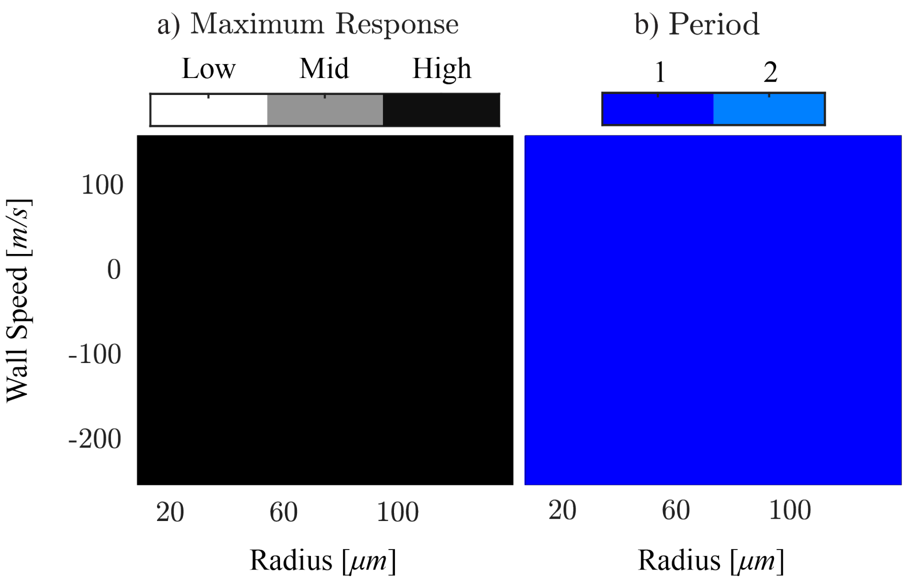

Figure S4.142: (a) the amplitude basins for bubble 10, where low-, mid- and high-amplitude attractor correspond to white, gray and black; and (b) the corresponding period basins of these attractors, as indicated by the colorbar.

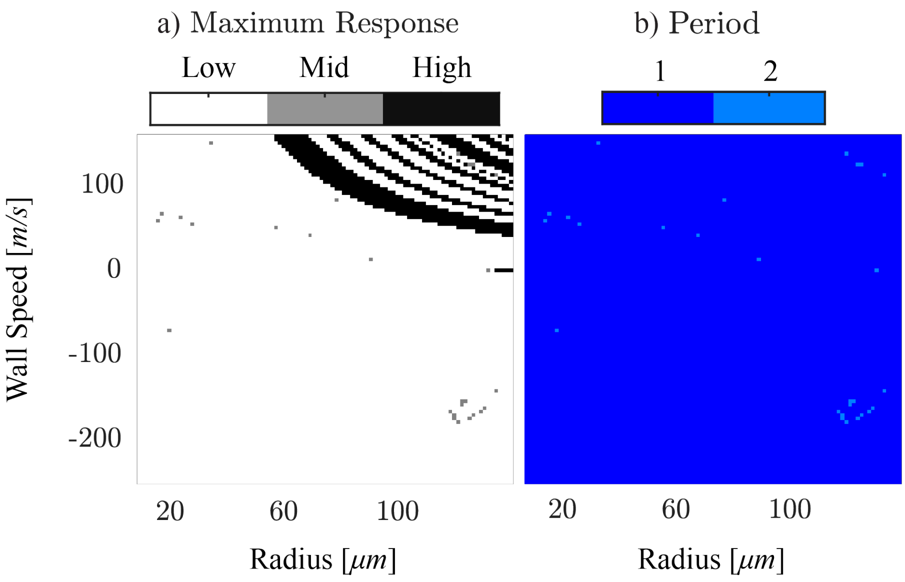

Figure S4.143: (a) the amplitude basins for bubble 11, where low-, mid- and high-amplitude attractor correspond to white, gray and black; and (b) the corresponding period basins of these attractors, as indicated by the colorbar.

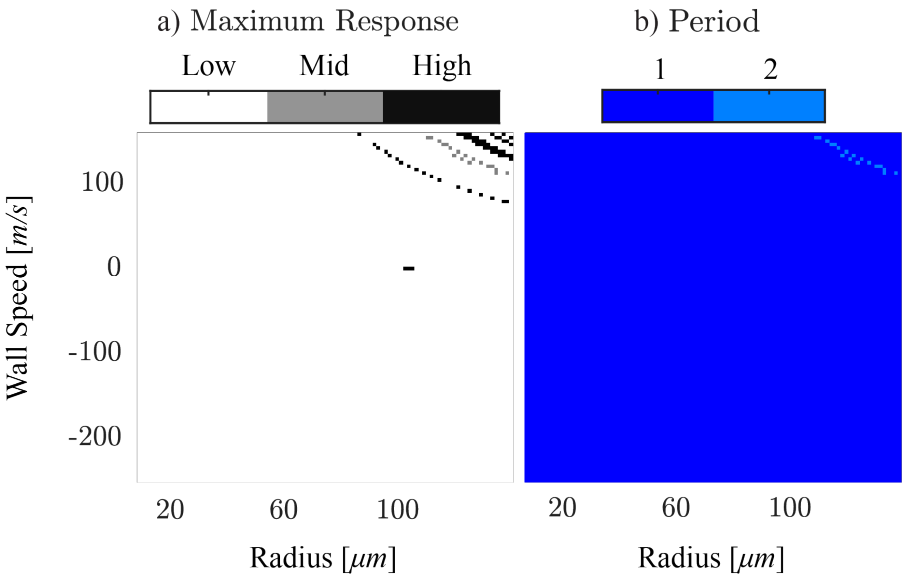

Figure S4.144: (a) the amplitude basins for bubble 12, where low-, mid- and high-amplitude attractor correspond to white, gray and black; and (b) the corresponding period basins of these attractors, as indicated by the colorbar.
