# Supplementary material for: Hysteresis in cavitation emissions during a ramped-then-deramped amplitude sonication: A theoretical and experimental investigation
Source: Nonlinear Dyn. 2026 Apr 21;114(8):591. doi: 10.1007/s11071-026-12462-3 (PMC13100018; doi:10.1007/s11071-026-12462-3)
Supplement: Supplementary file 5 — (pdf 1374 KB) [file 11071_2026_12462_MOESM5_ESM.pdf]

Hysteresis in cavitation emissions during a ramped-then-deramped amplitude sonication
A theoretical and experimental investigation

Supplementary Material 5: Additional Basin-of-attraction Analysis

Y. Zhang[1], S. Li[1], P. Prentice[1] and A. Cammarano[2]
[1]Cavitation Laboratory, Centre for Medical and Industrial Ultrasonics,
University of Glasgow, University Avenue, Glasgow, G12 8QQ, UK
[2]Department of Aeronautics and Astronautics,
University of Southampton, Burgess Road, Southampton, SO16 7QF, UK
email: andrea.cammarano@soton.ac.uk

Journal: Nonlinear Dynamics

1 Basin-of-attraction Analysis on Scaling Factor

To investigate how the strength of bubble–bubble interactions affects attractor selection, additional simulations are performed by varying the spatial scaling factor of the bubble distribution. The scaling factor modifies the inter-bubble distance while keeping all other parameters constant, thereby controlling the effective coupling strength between bubbles. Consistent with the main manuscript, bubble 2 is designated as the control bubble. Three representative cases are considered: (i) a scaling factor of 100, corresponding to negligible interactions where bubbles are positioned one hundred times farther apart than in the original configuration (see Fig. S5.1-S5.2); (ii) the original configuration with a scaling factor of 1, representing the baseline interaction strength (see Online Resource 3); and (iii) a scaling factor of 0.1, corresponding to a strong interaction regime where bubbles are positioned ten times closer together (see Fig. S5.3-S5.14).

1.1 Scaling Factor = 100

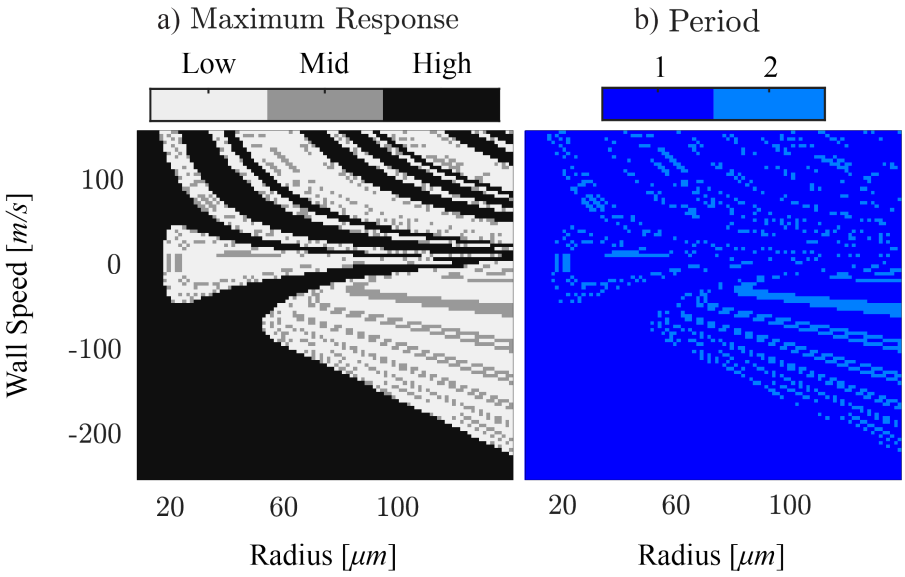

Figure S5.1: (a) the amplitude basin of bubble 2, where low-, mid- and high-amplitude attractor correspond to white, gray and black; and (b) the corresponding period basin of these attractors, as indicated by the colorbar.

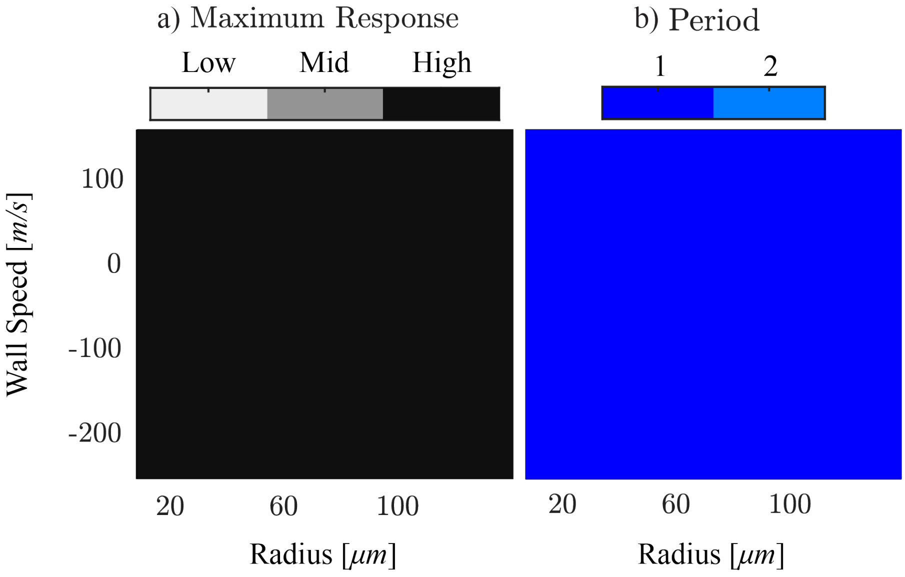

Figure S5.2: (a) the amplitude basin for bubbles, except bubble 2, where low-, mid- and high-amplitude attractor correspond to white, gray and black; and (b) the corresponding period basin of these attractors, as indicated by the colorbar.

1.2 Scaling Factor = 0.1

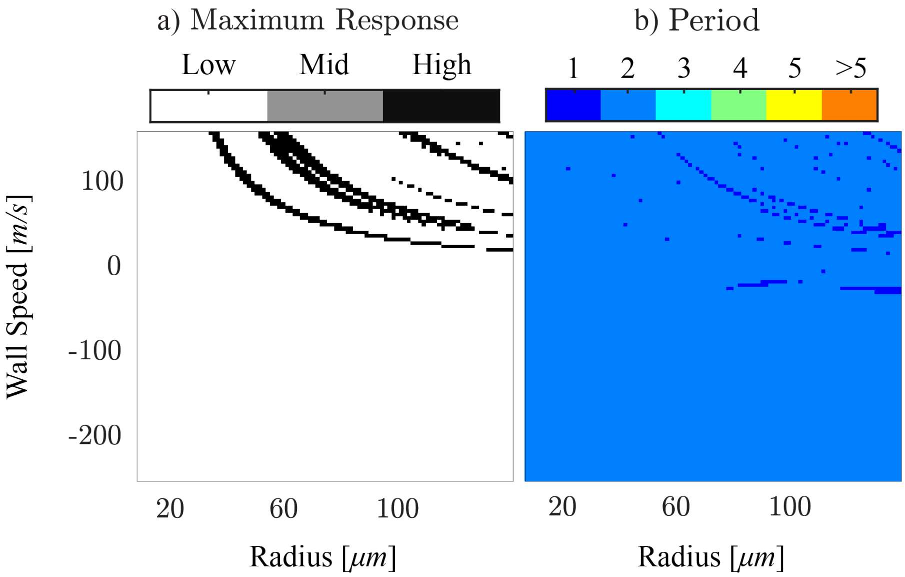

Figure S5.3: (a) the amplitude basins of bubble 1, where low-, mid- and high-amplitude attractor correspond to white, gray and black; and (b) the corresponding period basins of these attractors, as indicated by the colorbar.

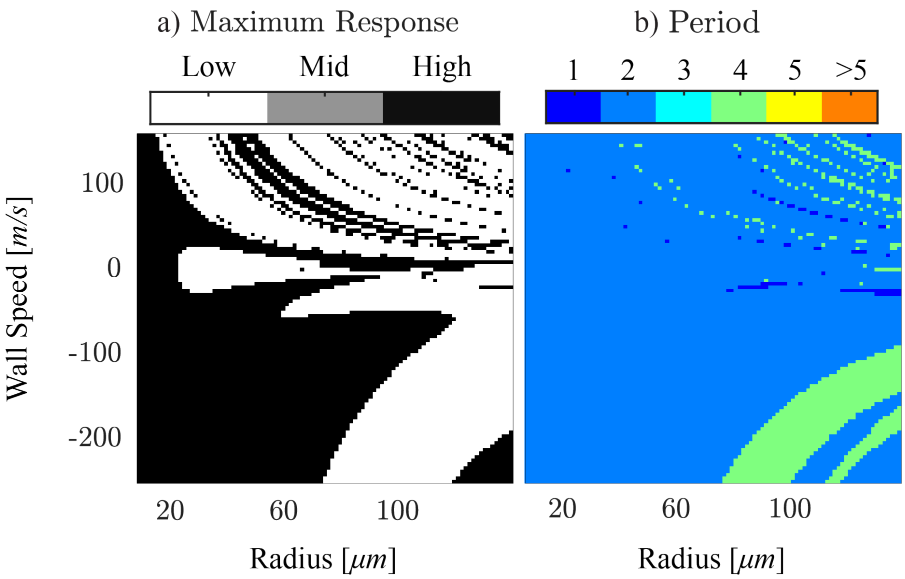

Figure S5.4: (a) the amplitude basins for bubble 2, where low-, mid- and high-amplitude attractor correspond to white, gray and black; and (b) the corresponding period basins of these attractors, as indicated by the colorbar.

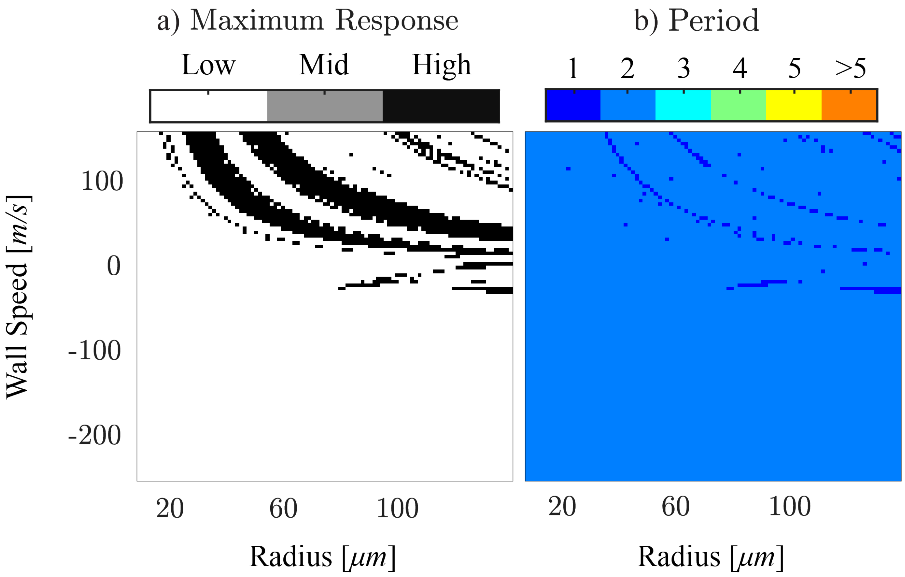

Figure S5.5: (a) the amplitude basins for bubble 3, where low-, mid- and high-amplitude attractor correspond to white, gray and black; and (b) the corresponding period basins of these attractors, as indicated by the colorbar.

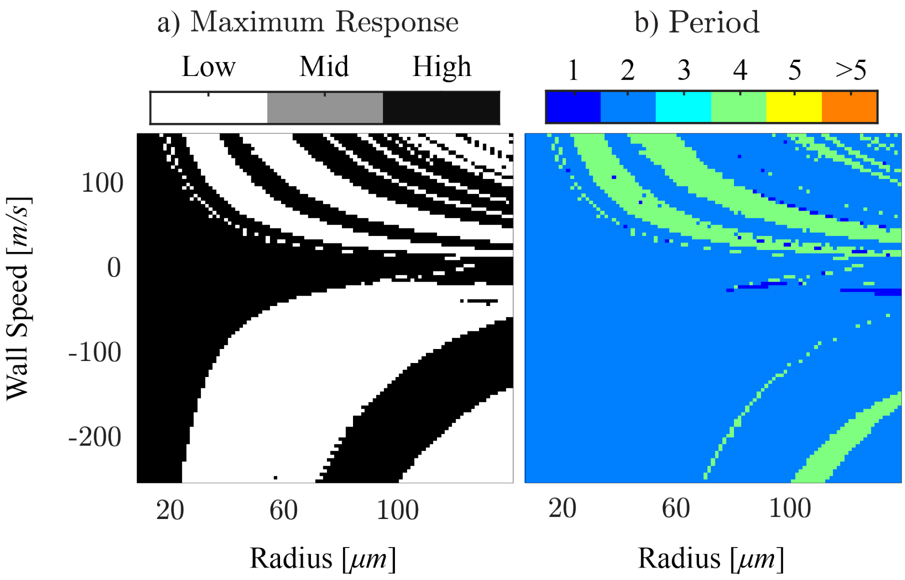

Figure S5.6: (a) the amplitude basins for bubble 4, where low-, mid- and high-amplitude attractor correspond to white, gray and black; and (b) the corresponding period basins of these attractors, as indicated by the colorbar.

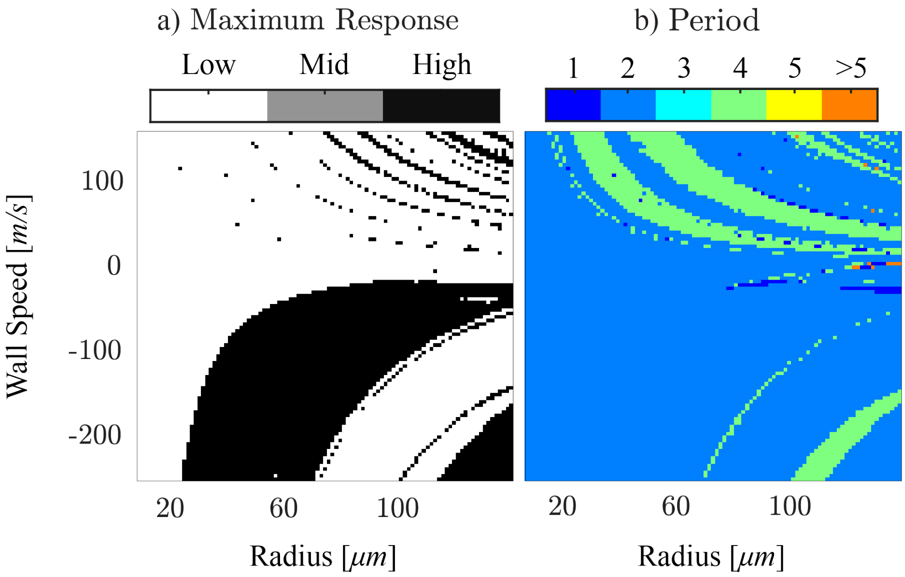

Figure S5.7: (a) the amplitude basins for bubble 5, where low-, mid- and high-amplitude attractor correspond to white, gray and black; and (b) the corresponding period basins of these attractors, as indicated by the colorbar.

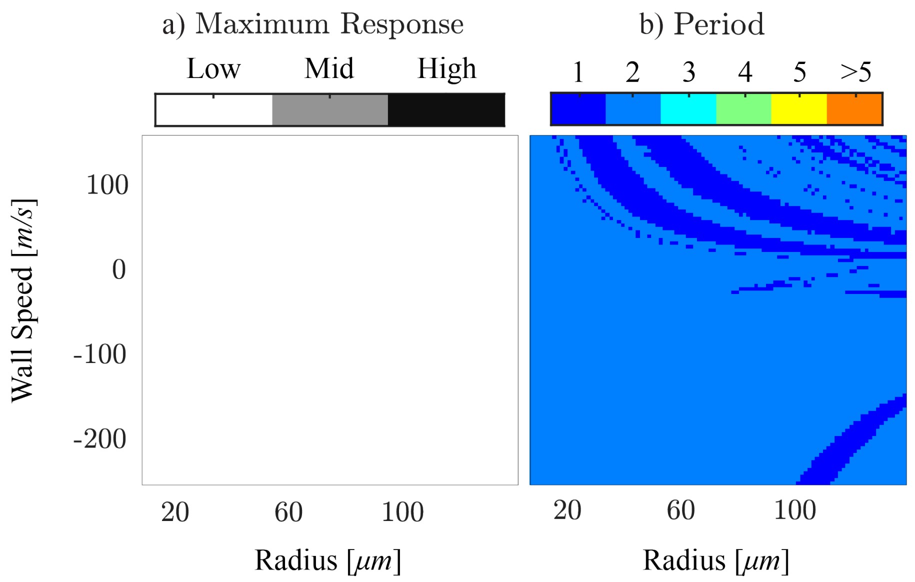

Figure S5.8: (a) the amplitude basins for bubble 6, where low-, mid- and high-amplitude attractor correspond to white, gray and black; and (b) the corresponding period basins of these attractors, as indicated by the colorbar.

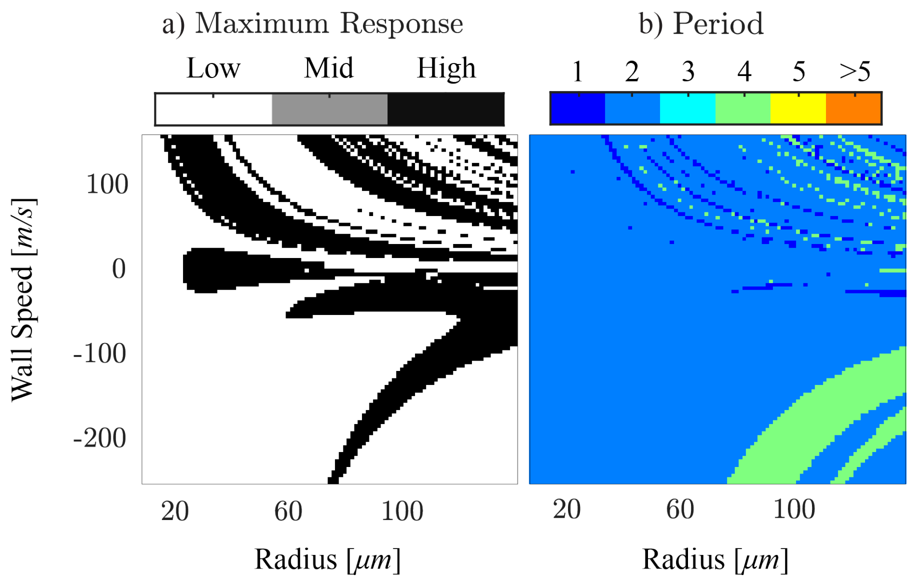

Figure S5.9: (a) the amplitude basins for bubble 7, where low-, mid- and high-amplitude attractor correspond to white, gray and black; and (b) the corresponding period basins of these attractors, as indicated by the colorbar.

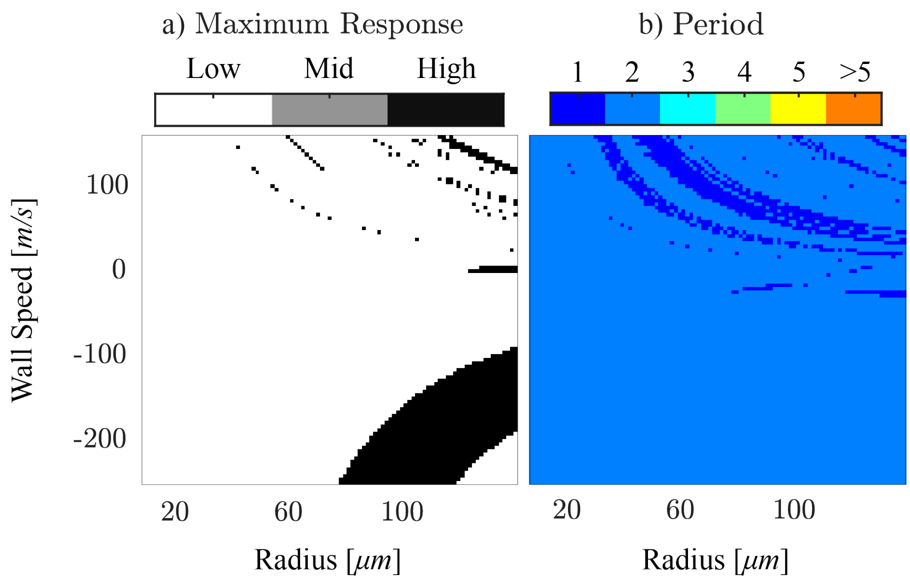

Figure S5.10: (a) the amplitude basins for bubble 8, where low-, mid- and high-amplitude attractor correspond to white, gray and black; and (b) the corresponding period basins of these attractors, as indicated by the colorbar.

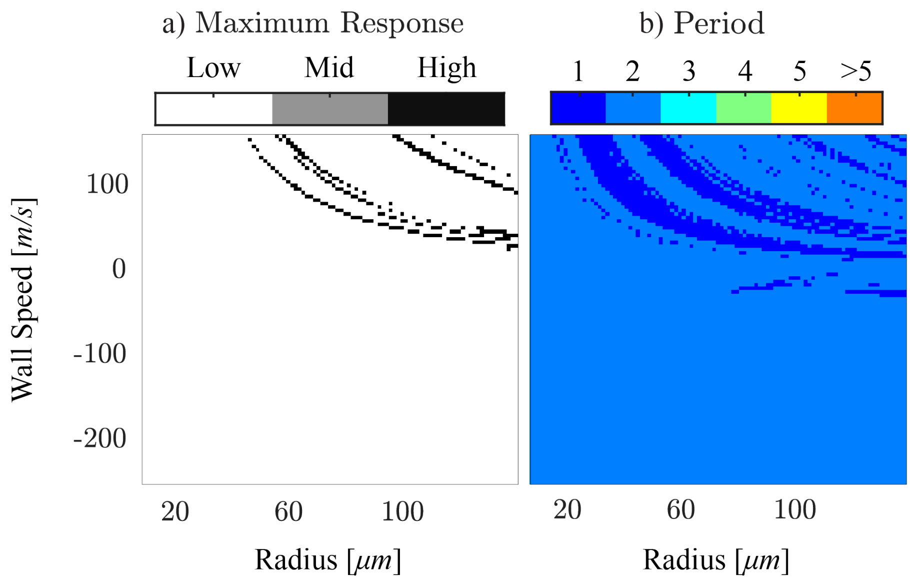

Figure S5.11: (a) the amplitude basins for bubble 9, where low-, mid- and high-amplitude attractor correspond to white, gray and black; and (b) the corresponding period basins of these attractors, as indicated by the colorbar.

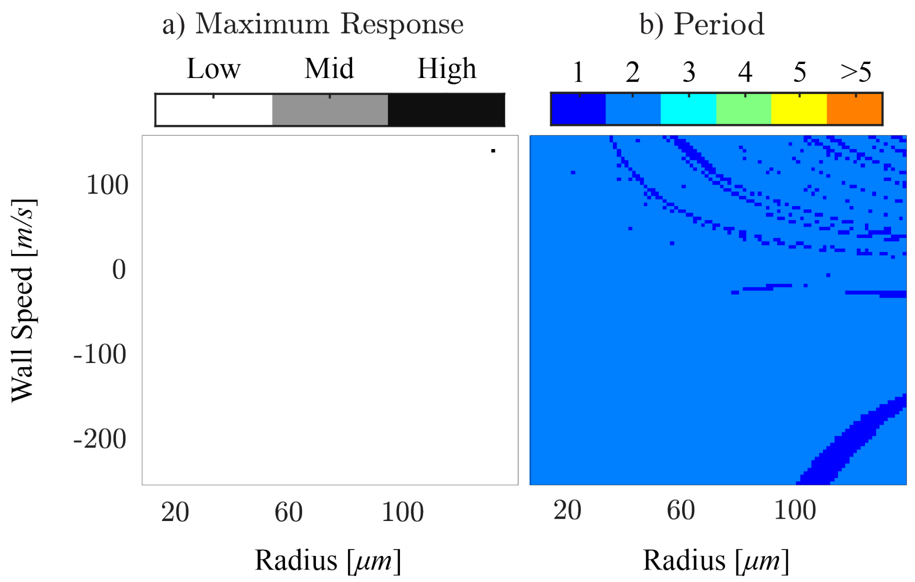

Figure S5.12: (a) the amplitude basins for bubble 10, where low-, mid- and high-amplitude attractor correspond to white, gray and black; and (b) the corresponding period basins of these attractors, as indicated by the colorbar.

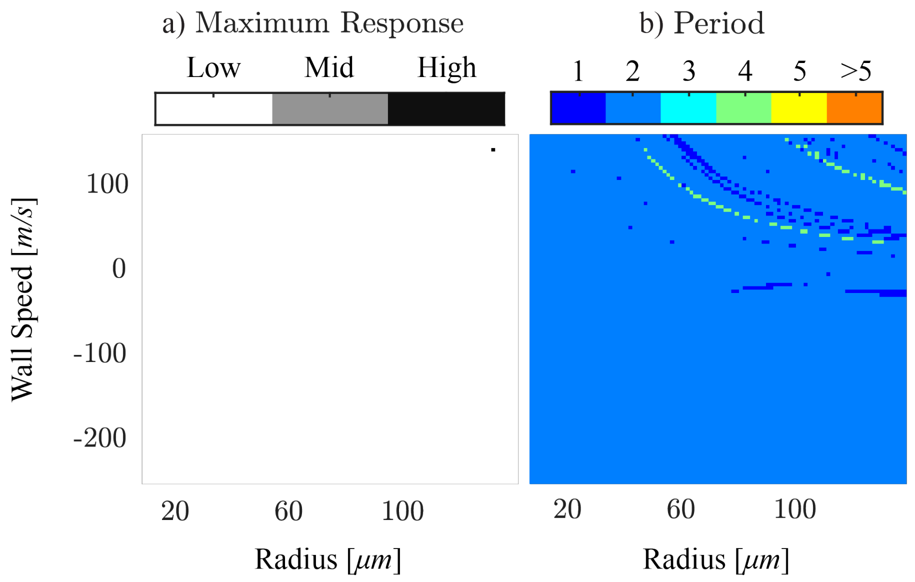

Figure S5.13: (a) the amplitude basins for bubble 11, where low-, mid- and high-amplitude attractor correspond to white, gray and black; and (b) the corresponding period basins of these attractors, as indicated by the colorbar.

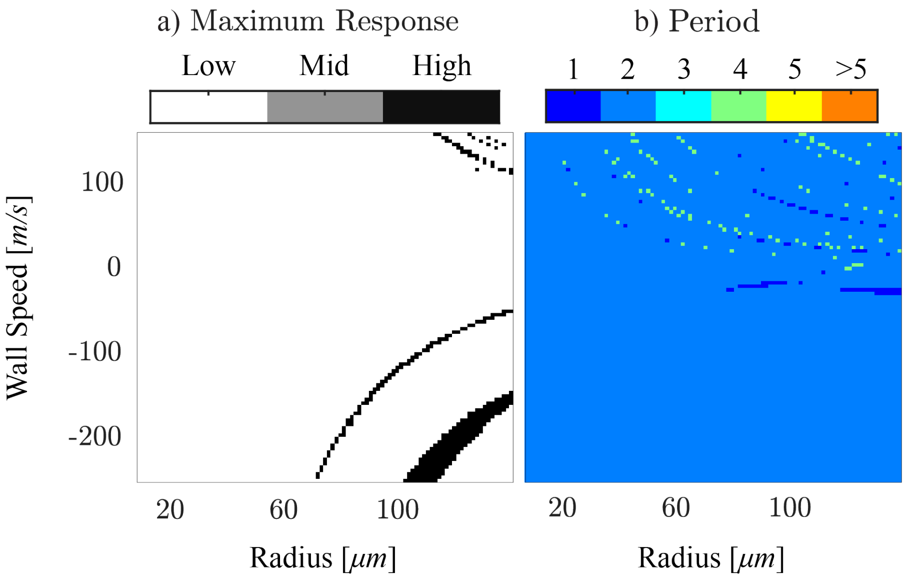

Figure S5.14: (a) the amplitude basins for bubble 12, where low-, mid- and high-amplitude attractor correspond to white, gray and black; and (b) the corresponding period basins of these attractors, as indicated by the colorbar.

2 Basin of Attraction under Direct Interaction

This supplementary figure presents the basins of attraction for all bubbles in the system when only direct interactions with the control bubble (bubble 2) are considered, and the scaling factor is restored to 1. Under these conditions, all bubbles, except the control bubble (bubble 2, see Fig. S5.15) and the closest neighbor (bubble 7, see Fig. S5.16), converge to high-amplitude, period-1 attractors, as shown in Fig. S5.17.

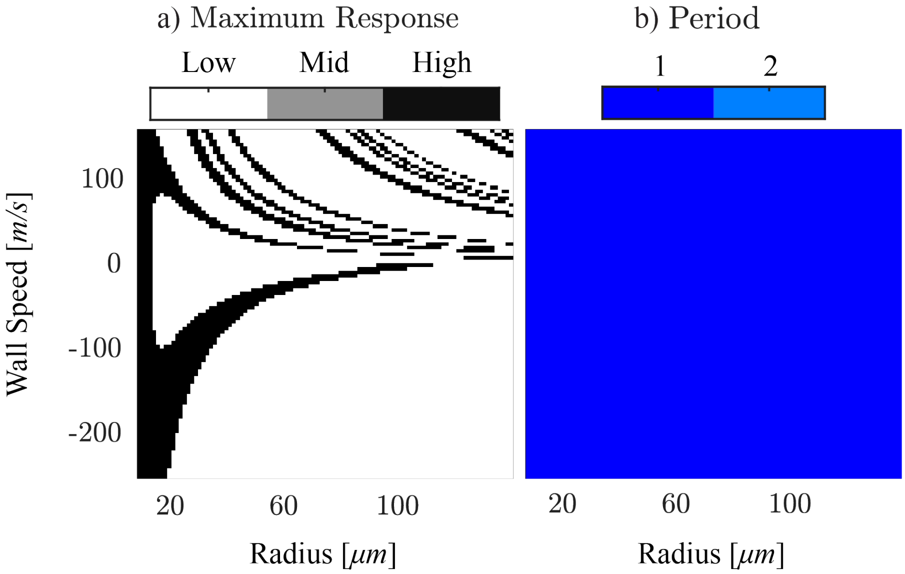

Figure S5.15: (a) the amplitude basin of bubble 2, where low-, mid- and high-amplitude attractor correspond to white, gray and black; and (b) the corresponding period basin of these attractors, as indicated by the colorbar.

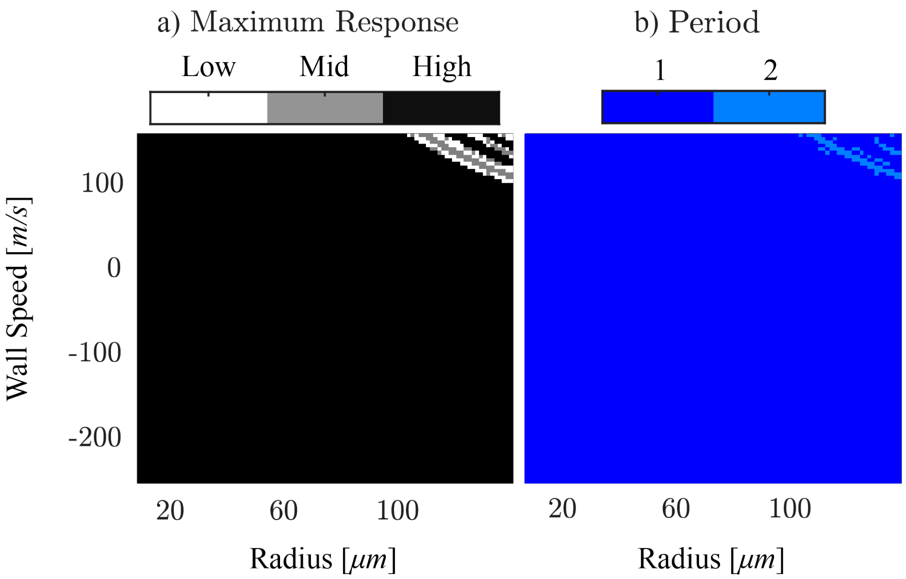

Figure S5.16: (a) the amplitude basin for bubble 7, where low-, mid- and high-amplitude attractor correspond to white, gray and black; and (b) the corresponding period basin of these attractors, as indicated by the colorbar.

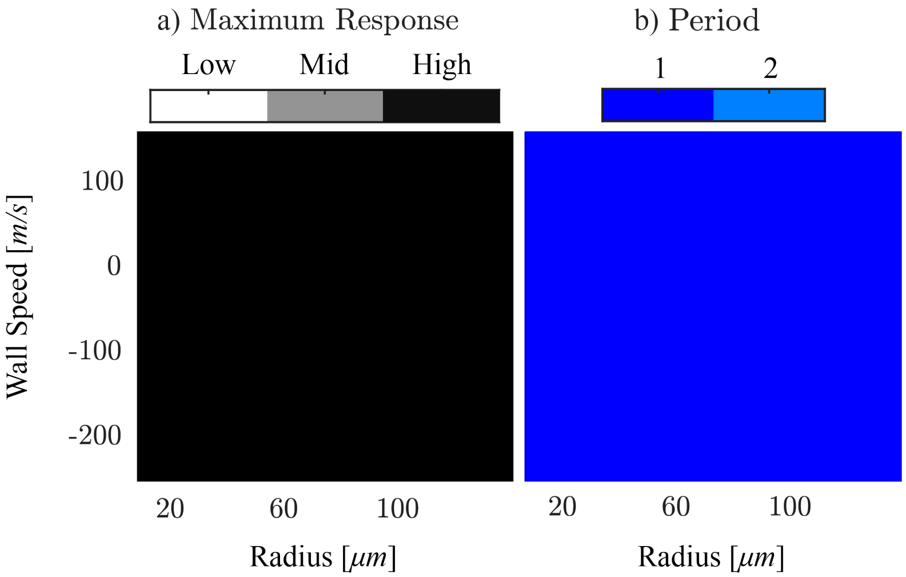

Figure S5.17: (a) the amplitude basins of bubbles excluding bubble 2 and 7, where low-, mid- and high-amplitude attractor correspond to white, gray and black; and (b) the corresponding period basins of these attractors, as indicated by the colorbar.
