# Supplementary material for: Hysteresis in cavitation emissions during a ramped-then-deramped amplitude sonication: A theoretical and experimental investigation
Source: Nonlinear Dyn. 2026 Apr 21;114(8):591. doi: 10.1007/s11071-026-12462-3 (PMC13100018; doi:10.1007/s11071-026-12462-3)
Supplement: Supplementary file 6 — (pdf 1257 KB) [file 11071_2026_12462_MOESM6_ESM.pdf]

Hysteresis in cavitation emissions during a ramped-then-deramped amplitude sonication

A theoretical and experimental investigation

Supplementary Material 6: Repeatability Analysis

Y. Zhang[1], S. Li[1], P. Prentice[1] and A. Cammarano[2]
[1]Cavitation Laboratory, Centre for Medical and Industrial Ultrasonics,
University of Glasgow, University Avenue, Glasgow, G12 8QQ, UK
[2]Department of Aeronautics and Astronautics,
University of Southampton, Burgess Road, Southampton, SO16 7QF, UK
email: andrea.cammarano@soton.ac.uk

Journal: Nonlinear Dynamics

A selection of spectrograms obtained under the same excitation parameters as the ramped–then–deramped amplitude experiment is presented in Fig. 1. Despite some variability, the main features discussed in this study remain consistent across all measurements. In particular, panels (a–d) demonstrate that the broadband regions appear asymmetrically with respect to excitation amplitude: the initial emergence of broadband noise during the ramp requires a higher excitation amplitude than the re-emergence observed during the deramp.

In the regions from 0-69 ms and from 131-114.4 ms, there are some variability in the occurrence and duration of broadband noise and the prominence of $nf_0/4$ emissions, but this is explained by the fact that we do not have full control on the initial conditions of cavitation within the tube transducer bore and therefore different initial states can lead to different responses especially in neighbourhoods of the bifurcation points.

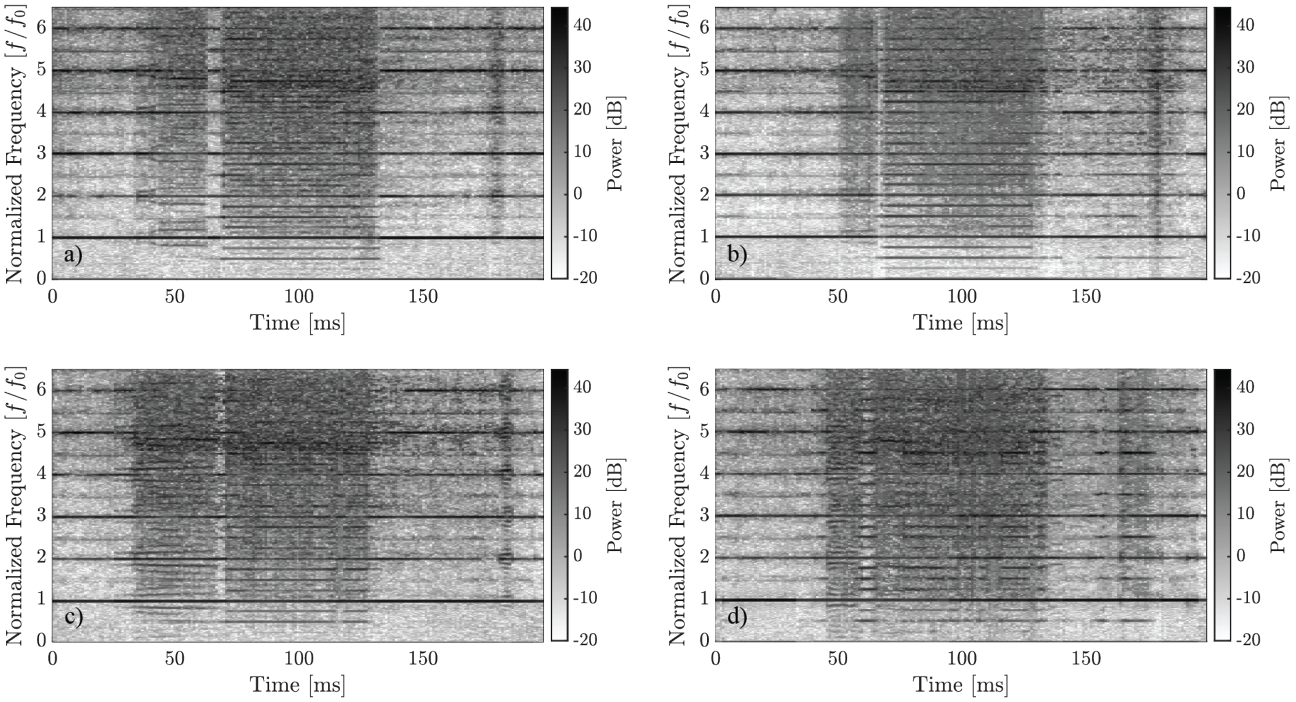

Figure 1: A selection of spectrograms with the same sonication parameters as the main manuscript.
