# Supplementary material for: Hysteresis in cavitation emissions during a ramped-then-deramped amplitude sonication: A theoretical and experimental investigation
Source: Nonlinear Dyn. 2026 Apr 21;114(8):591. doi: 10.1007/s11071-026-12462-3 (PMC13100018; doi:10.1007/s11071-026-12462-3)
Supplement: Supplementary file 7 — (pdf 733 KB) [file 11071_2026_12462_MOESM7_ESM.pdf]

Hysteresis in cavitation emissions during a ramped-then-deramped amplitude sonication
A theoretical and experimental investigation
Supplementary Material 7: Polydispersity

Y. Zhang[1], S. Li[1], P. Prentice[1] and A. Cammarano[2]
[1]Cavitation Laboratory, Centre for Medical and Industrial Ultrasonics,
University of Glasgow, University Avenue, Glasgow, G12 8QQ, UK
[2]Department of Aeronautics and Astronautics,
University of Southampton, Burgess Road, Southampton, SO16 7QF, UK
email: andrea.cammarano@soton.ac.uk

Journal: Nonlinear Dynamics

The synchronization of polydisperse systems has been studied in detail by Nasibullaeva and Akhatov [1] and Tervo *et al.* [2]. They showed that, although isolated bubbles in a polydisperse population collapse at different times, coupling via pressure emissions can shift collapse timing and reduce phase offsets, ultimately producing synchronized oscillations across the population. Our recent work [3] further linked broadband noise to desynchronization and broadband-noise clearing to synchronization. On this basis, the hysteresis of the broadband-noise strength reported in the main manuscript is expected to exhibit a similar trend in both polydisperse and monodisperse systems.

For completeness, we applied the ramped–then-deramped excitation in Fig. 4(a) of the main manuscript to two different polydisperse systems (A and B) with the same bubble configuration as in Fig. 3 of the main manuscript. In polydisperse system A, the equilibrium radius of bubble 1 was set to 54.3 μm, while bubbles 2-12 were set to 52.5 μm. In polydisperse system B, each bubble is assigned a distinct equilibrium radius, specifically, $R_{01} = 54.3\ \mu m$, $R_{02} = 54.14\ \mu m$, $R_{03} = 53.97\ \mu m$, $R_{04} = 53.81\ \mu m$, $R_{05} = 53.65\ \mu m$, $R_{06} = 53.48\ \mu m$, $R_{07} = 53.32\ \mu m$, $R_{08} = 53.15\ \mu m$, $R_{09} = 52.99\ \mu m$, $R_{010} = 52.83\ \mu m$, $R_{011} = 52.66\ \mu m$, $R_{012} = 52.5\ \mu m$. The resulting numerical spectrograms (Fig. 1 and Fig. 2) show the same qualitative hysteresis displayed in Fig. 4(b) of the main manuscript.

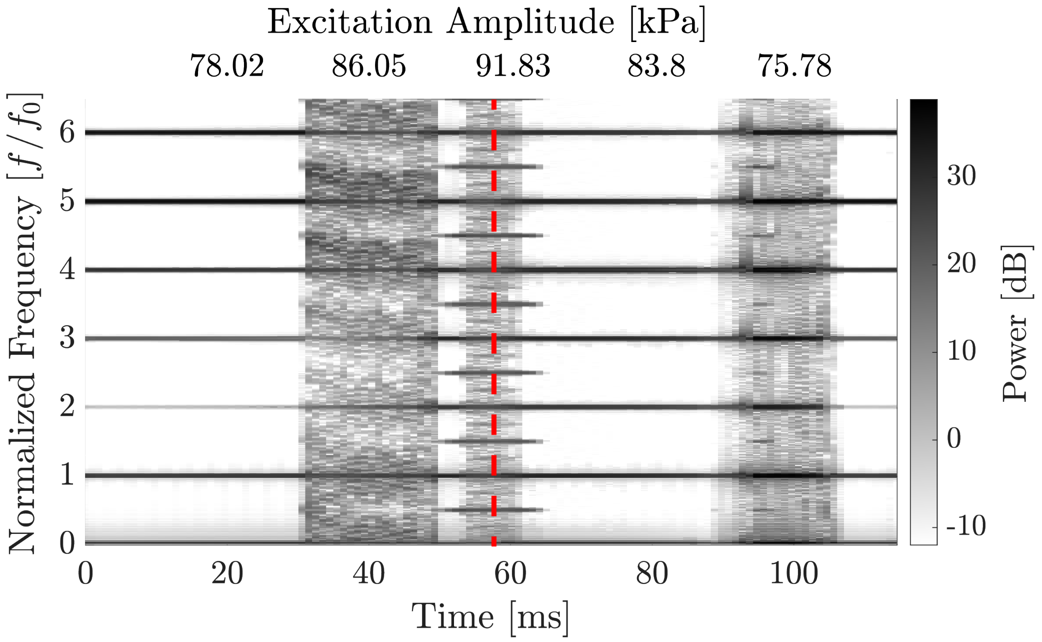

Figure 1: Numerical spectrogram of the polydisperse system A, where R_{01} = 54.3 μm and R_{0q} = 54.3 μm (q = 2,3, ..., 12). The vertical red dashed line denotes the symmetrical fold of the excitation amplitude.

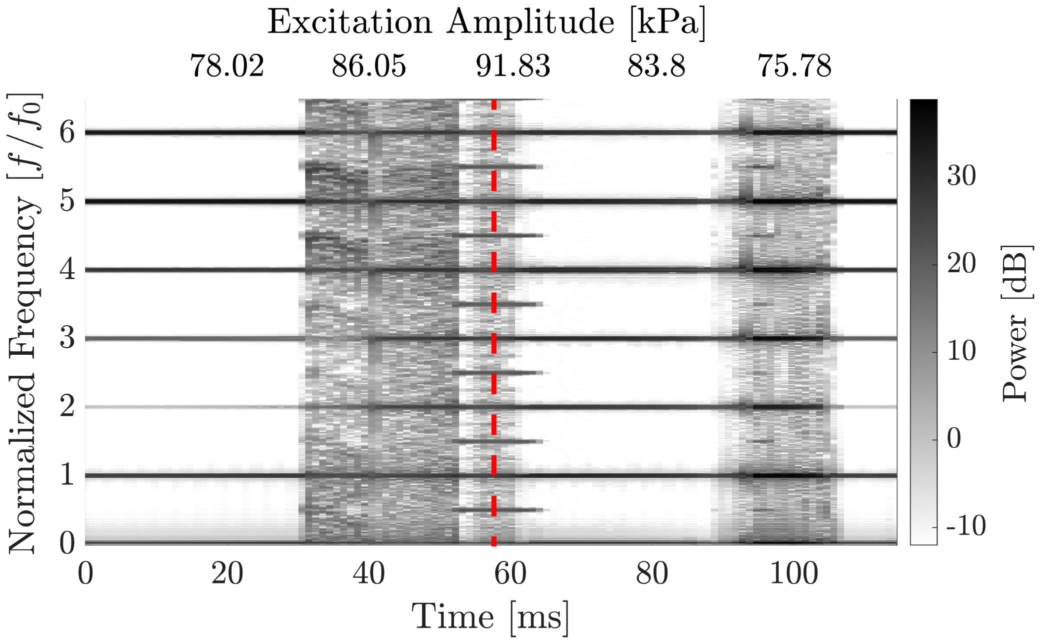

Figure 2: Numerical spectrogram of the polydisperse system B, where R_{01} = 54.3 μm, R_{02} = 54.14 μm, R_{03} = 53.97 μm, R_{04} = 53.81 μm, R_{05} = 53.65 μm, R_{06} = 53.48 μm, R_{07} = 53.32 μm, R_{08} = 53.15 μm, R_{09} = 52.99 μm, R_{010} = 52.83 μm, R_{011} = 52.66 μm, R_{012} = 52.5 μm. The vertical red dashed line denotes the symmetrical fold of the excitation amplitude.

References

[1] Elvira S Nasibullaeva and IS Akhatov. Bubble cluster dynamics in an acoustic field. *The Journal of the Acoustical Society of America*, 133(6):3727–3738, 2013.

[2] Jan Topi Tervo, Robert Mettin, and Werner Lauterborn. Bubble cluster dynamics in acoustic cavitation. *Acta acustica united with acustica*, 92(1):178–180, 2006.

[3] Hilde Metzger, Yikai Zhang, Andrea Cammarano, and Paul Prentice. Revisiting the subharmonic route to acoustic chaos: broadband noise clearing via cavitation bubble synchronization. *Proceedings of the Royal Society A*, 481(2317):20250048, 2025.
